# Supplementary material for: Structural analyses uncover protease-adhesin interactions and c-di-GMP receptor regulation in sulfate-reducing bacteria
Source: Nat Commun. 2026 Apr 17;17:3564. doi: 10.1038/s41467-026-71936-5 (PMC13090378; doi:10.1038/s41467-026-71936-5)
Supplement: Supplementary file 1 — Supplementary Information [file 41467_2026_71936_MOESM1_ESM.pdf]

Structural analyses uncover protease-adhesin interactions and c-di-GMP receptor regulation in sulfate-reducing bacteria

Maria E. Font, Amruta A. Karbelkar, Justin D. Lormand, Sofia Mortensen, Maria J. Garcia-Garcia, George A. O’Toole, Holger Sondermann

Supplementary Information

Supplementary Fig. 1-8
Supplementary Tables 1-9
Supplementary References

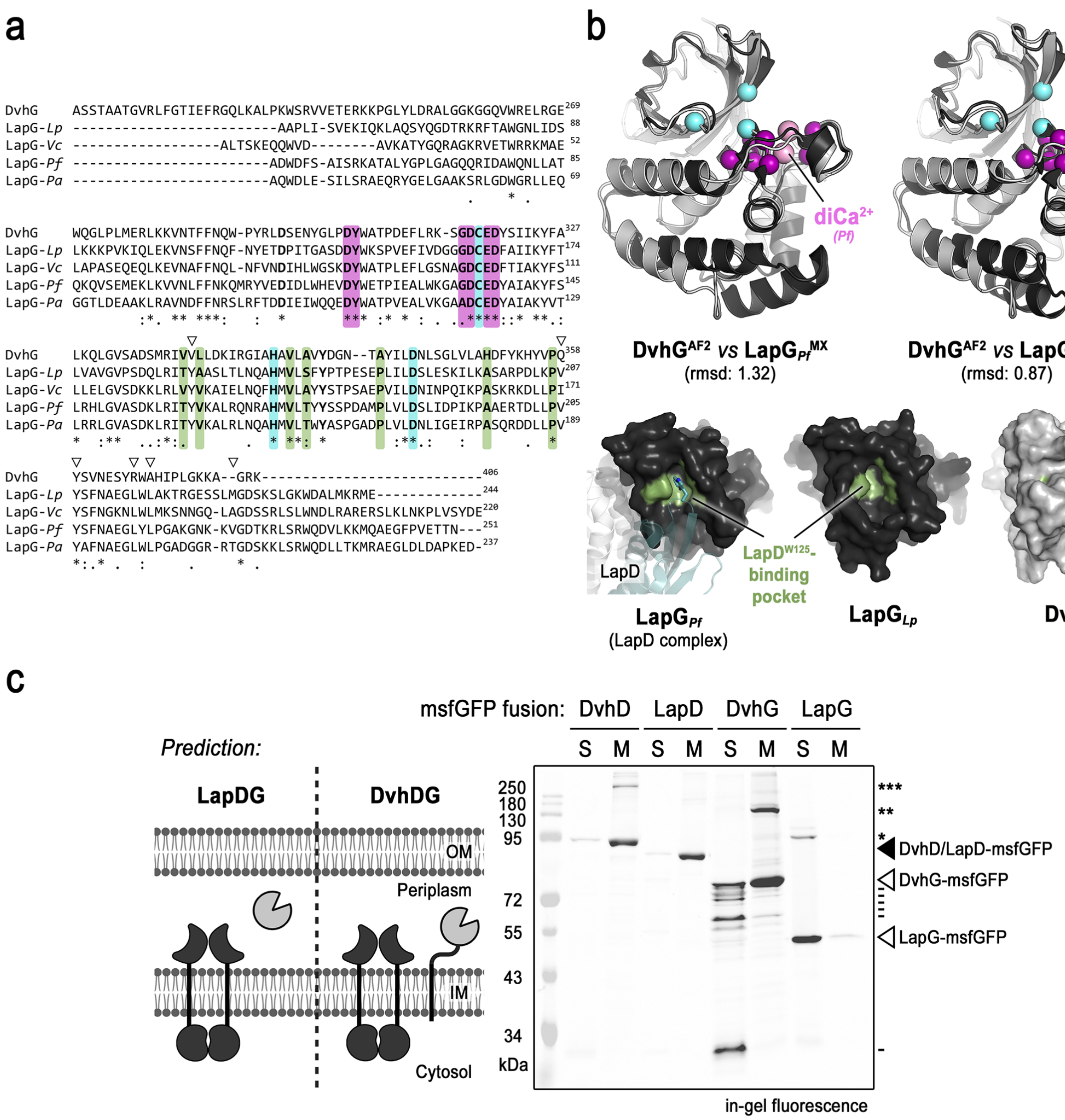

Supplementary Fig. 1. **Comparison between LapG orthologs and DvhG. a** Protein sequence alignment of DvhG and gammaproteobacterial LapG orthologs including *Legionella pneumophila* (*Lp*), *Vibrio cholerae* (*Vc*), *Pseudomonas fluorescens* (*Pf*), and *Pseudomonas aeruginosa* (*Pa*). The location of the catalytic triad (light blue), calcium binding motif (purple), and LapD-binding pocket (green) are indicated. **b** Comparison between gammaproteobacterial LapG crystal structures and DvhG. In lieu of an experimentally determined structure of DvhG, we used AF2 to generate high-confidence structural models. The model contains a folded C-terminal domain that superimposes well with experimentally determined LapG structures from *P. fluorescens* and *L. pneumophila* with root-mean-square deviation (rmsd) of 1.32 Å and 0.87 Å, respectively[1,2] . Accordingly, the putative catalytic triad (light blue spheres) and calcium-binding motif (purple spheres) of the DvhG three-dimensional AF2 model align with the corresponding residues in the crystal structures of gammaproteobacterial LapG orthologs. The predicted conformation around the calcium-binding site of DvhG suggests coordination of a di-calcium, as observed in *P. fluorescens* LapG. Surface renditions (bottom panel) focus on the Trp-dependent docking site for LapD on LapG (green surface). This region appears to be occluded in the DvhG model. **c** Subcellular localization of DvhG, LapG, DvhD, and LapD. The proteins were expressed fused to monomeric super-folder GFP (msfGFP) in *E. coli* BL21 cells. Soluble and membrane fractions were separated, resolved on an SDS-PAGE and imaged for in-gel fluorescence. LapG and DvhG show a second, fluorescent band about twice the size of the monomeric protein, which may present homodimers (indicated by *, **, ***). In the unboiled samples, proteins migrate as partially folded entities, retaining the folded state of the msfGFP moiety, rendering size estimations based on migratory behavior unreliable. Predicted topologies are shown in the diagram (left panel). Schematic in panel c: Created in BioRender. Sondermann, H. (2026) https://BioRender.com/2hdwdgf.

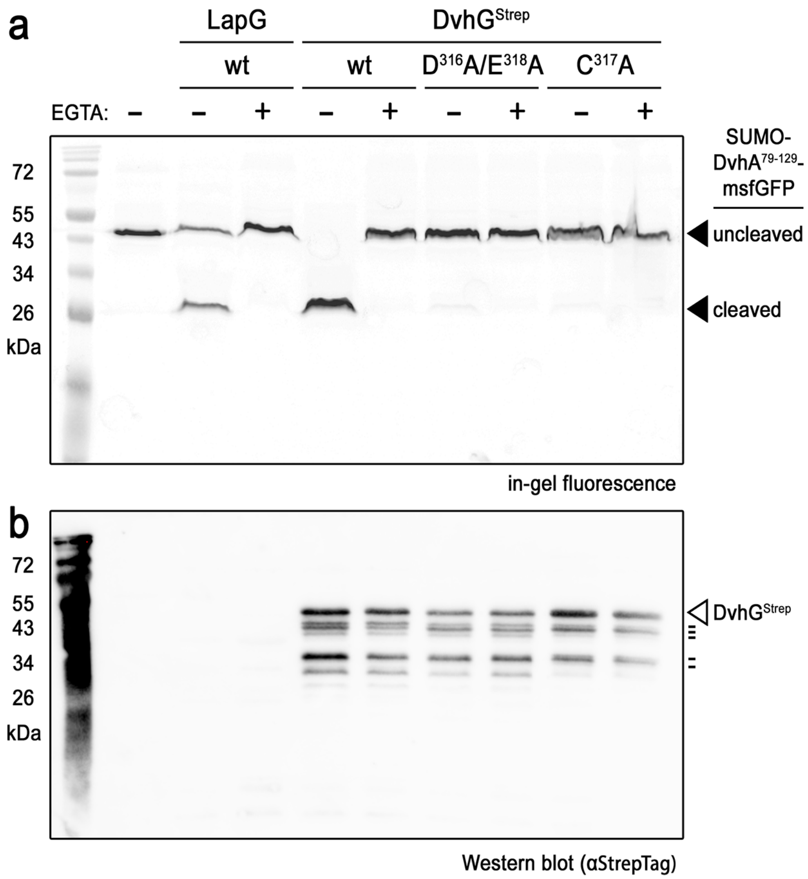

Supplementary Fig. 2. **Proteolysis of DvhA by specific DvhG mutant variants. a** Cleavage reactions were comprised of purified DvhA-based substrate reporter and cell lysates containing either DvhG wild-type or mutant variants with disrupted calcium-binding site or catalytic triad. Cleavage was assessed through in-gel fluorescence of reaction products after SDS-PAGE. **b** Samples identical to panel **a** were diluted 1:10, followed by SDS-PAGE and Western blotting with detection of the StrepII-tag at the C-terminus of full-length DvhG to assess mutant protein stability. Notably, wild-type and inactive protease variants showed similar proteolytic patterns in lysates, ruling out autoproteolysis as a source of DvhG processing.

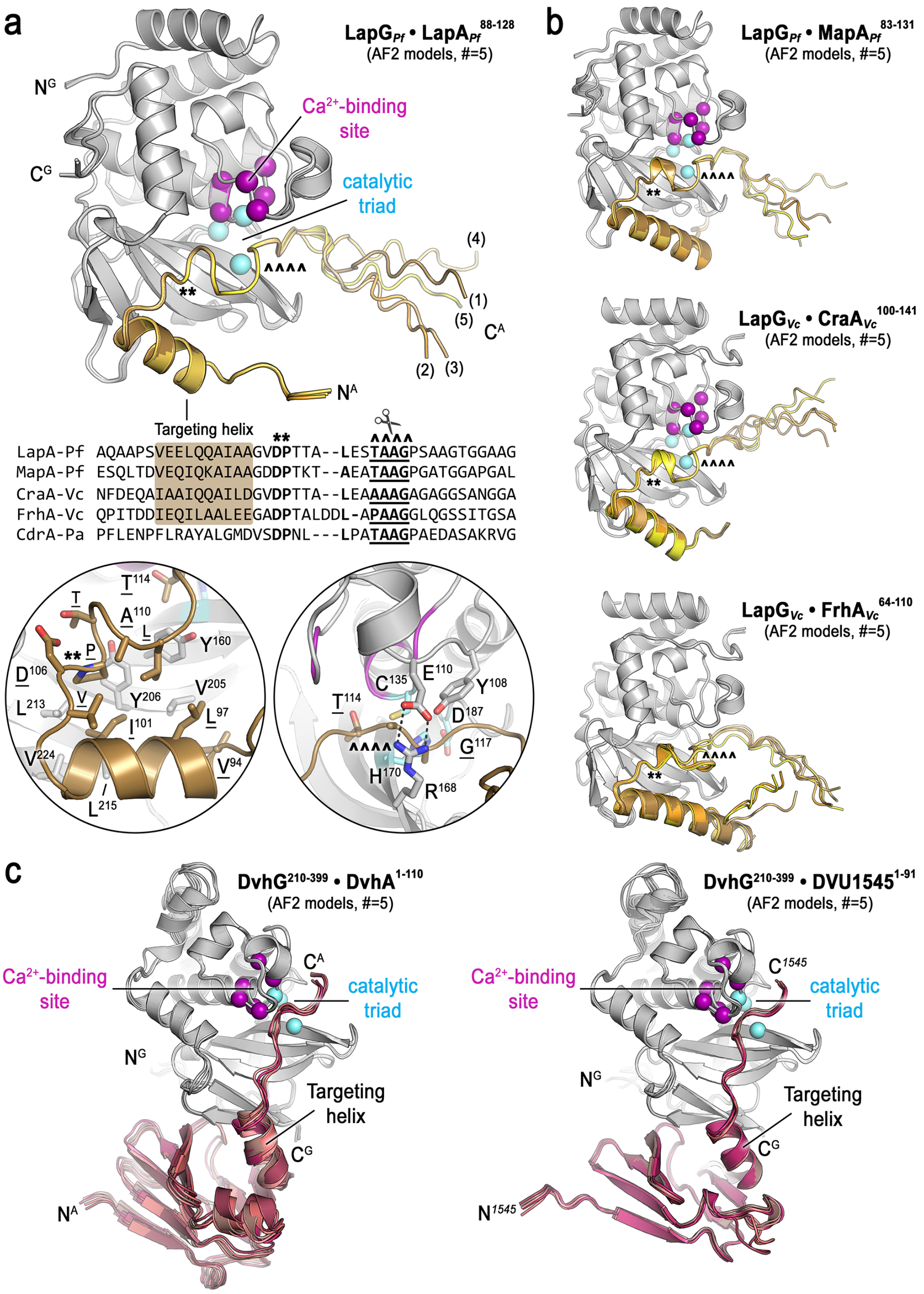

Supplementary Fig. 3. **Modeling of protease-substrate complexes.** For all predictions, five models were generated using default parameters and shown superimposed. Signal peptides or transmembrane and flexible regions were omitted from LapG or DvhG prior to modeling. For LapG orthologs, the substrate sequences span the targeting helix, conserved DP motif (**), and consensus cleavage site (^^^^), flanked by six and ten residues N- and C-terminally, respectively; see sequence alignment in panel **a**. Adhesin residues are underlined. DvhG was modeled in complex with substrates spanning the entire N-terminal retention module up to the consensus cleavage site. The following complexes are shown: **a** LapG$_{Pf}$•LapA$_{Pf}$; **b** LapG$_{Pf}$•MapA$_{Pf}$, LapG$_{Vc}$•CraA$_{Vc}$, and LapG$_{Vc}$•FrhA$_{Vc}$; and **c** DvhG•DvhA and DvhG•DVU1545. Insets in panel **a** show representative detailed views of the targeting helix-LapG and cleavage site-catalytic triad interfaces of the LapG$_{Pf}$•LapA$_{Pf}$ complex model. Proteases are colored grey throughout.

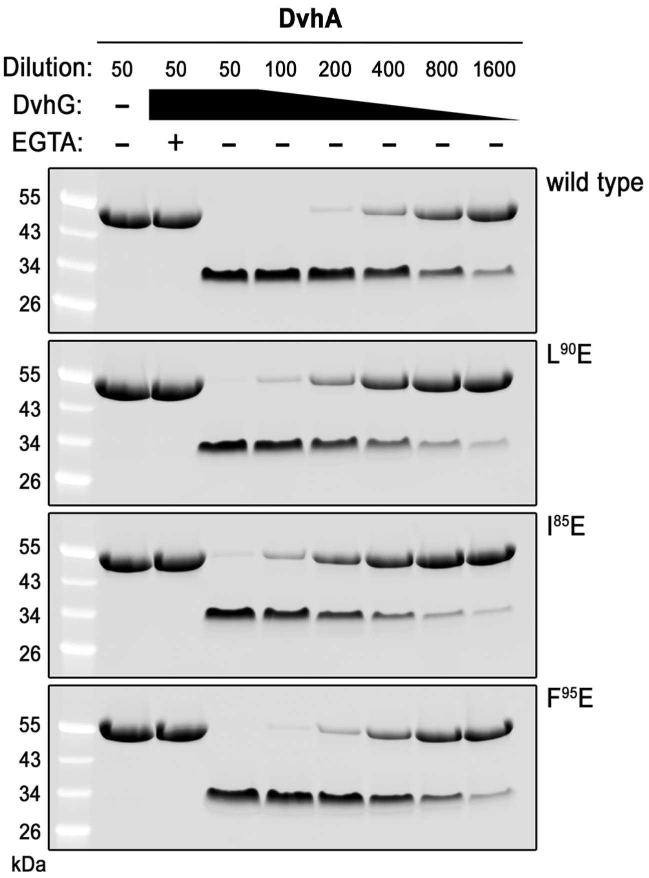

Supplementary Fig. 4. **Effect of DvhA mutations at the DvhA-DvhG interface.** Retention domain-containing substrate reporters with wild-type sequence or harboring single point mutations were purified and incubated with serially diluted lysates from *E. coli* cultures that expressed full-length DvhG. A parallel reaction at the highest lysate concentration was supplemented with EGTA. Reactions containing *E. coli* lysates harboring an empty plasmid were added at the highest concentration only.

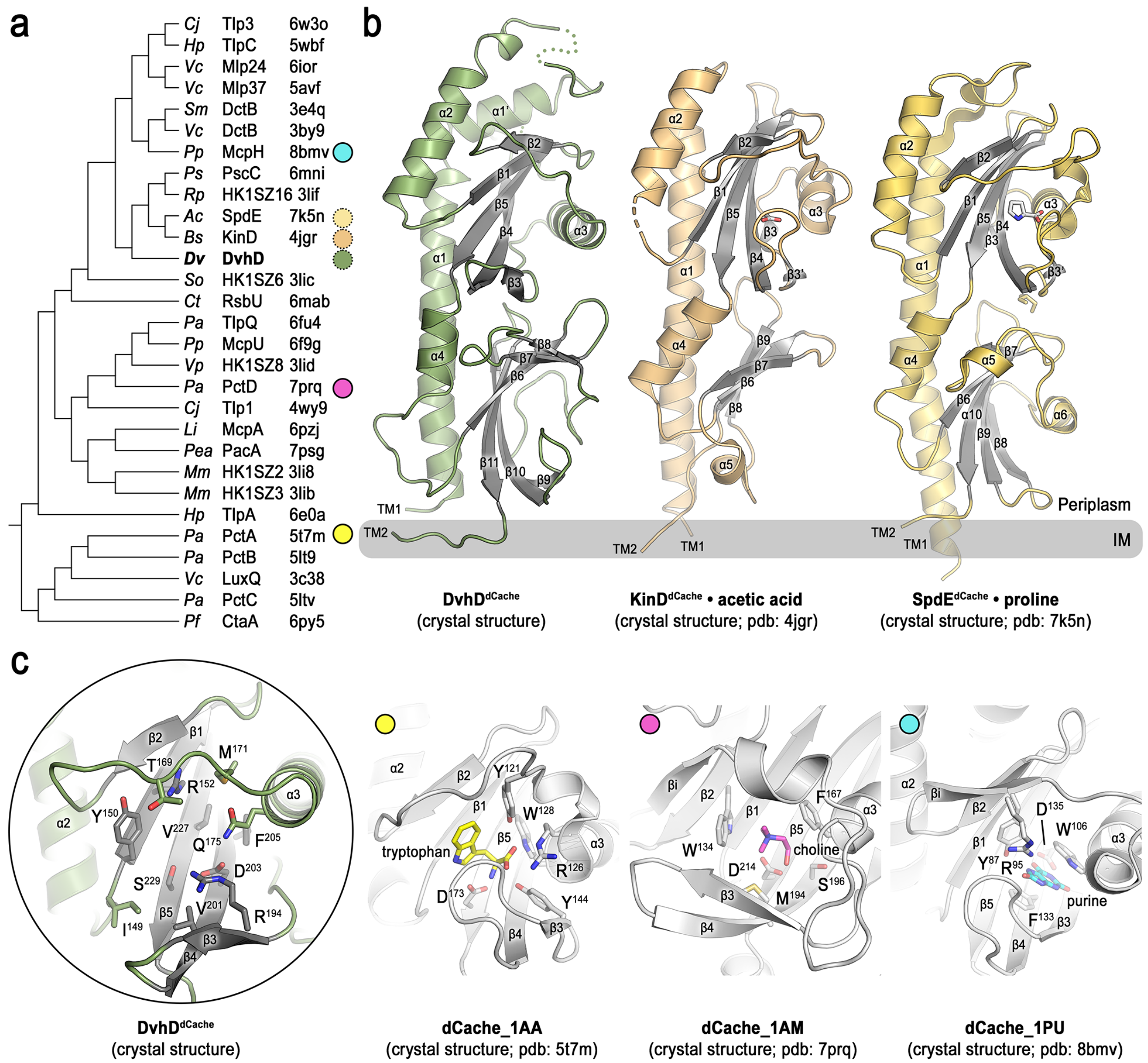

Supplementary Fig. 5. **Comparison of dCache domains.** **a** Identification of structural similarity between DvhD's periplasmic domain and dCache domains using Foldseek and FoldMason. Nonredundant representatives identified using a Foldseek search (hits had a probability score = 1 and sequence identity ≤ 24%) were used to generate multiple-protein structure with FoldMason. Species, protein names, and PDB codes are listed. *Cj*, *Campylobacter jejuni*; *Hp*, *Helicobacter pylori*; *Vc*, *Vibrio cholerae*, *Sm*, *Sinorhizobium meliloti*; *Pp*, *Pseudomonas putida*; *Ps*, *Pseudomonas syringae*; *Rp*, *Rhodopseudomonas palustris*; *Ac*, *Aeromonas caviae*; *Bs*, *Bacillus subtilis*; *Dv*, *Desulfovibrio vulgaris*; *So*, *Shewanella oneidensis*; *Ct*, *Chlamydia trachomatis*; *Pa*, *Pseudomonas aeruginosa*; *Vp*, *Vibrio parahaemolyticus*; *Li*, *Leptospira interrogans*; *Pea*, *Pectobacterium atrosepticum*; *Mm*, *Methanosarcina mazei*; *Pf*, *Pseudomonas fluoercens*. **b** Side-by-side global comparison. The two closest structural dCache domain homologs to DvhD's periplasmic domain are shown (pdb 4jgr[3] ; pdb 7k5n[4]). Corresponding secondary structure motifs are labeled. **c** Comparison of ligand binding sites of dCache domains. Close-up views of DvhD's distal lobe and ligand-bound states of representative dCache_1 domains (pdb 5t7m[5] ; pdb 7prq[6] ; pdb 8bmv[7]). Corresponding secondary structure motifs are labeled. Strand βi in dCache_1AM and dCache_1PU refers to an additional β strand not present in all dCache domains, which follows and folds back on strand β2.

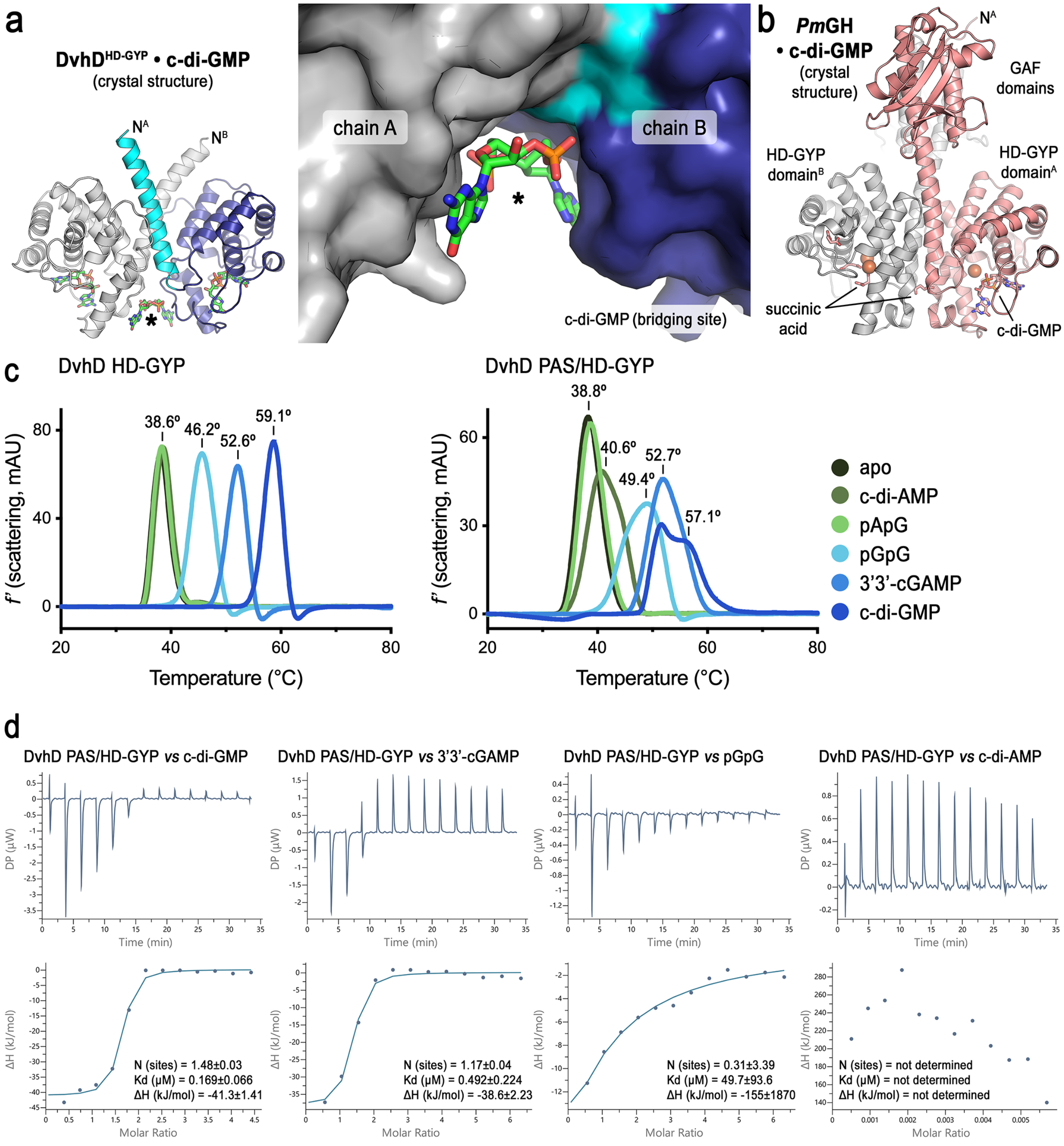

Supplementary Fig. 6. **Nucleotide binding to DvhD. a** The surface presentation shows the shape complementarity between DvhD and c-di-GMP at the bridging site. **b** The dimeric GAF/HD-GYP domain structure of *Pm*GH bound to c-di-GMP is shown (pdb 4mdz[8]). **c** The thermostability, as measured by nDSF, of DvhD's HD-GYP domain (left panel) or the PAS/HD-GYP tandem module (right panel) varies with the type of nucleotide added at saturating conditions. Representative data for three independent experiments is shown as the first derivative of the scattering curve upon heat denaturation (main-peak temperatures are indicated). **d** Characterization of ligand binding to the PAS/HD-GYP domain of DvhD. Isothermal titration calorimetry was used to determine the apparent affinity (expressed as K_d) of the PAS/HD-GYP domain of DvhD for c-di-GMP, 3'3'-cGAMP, pGpG, and c-di-AMP. The top graphs show the titration experiments, the bottom graphs show the fits of a single-binding site model to the data. Shown are representative plots from three independent experiments. Binding characteristics are reported in the inset and in Supplementary Table 4.

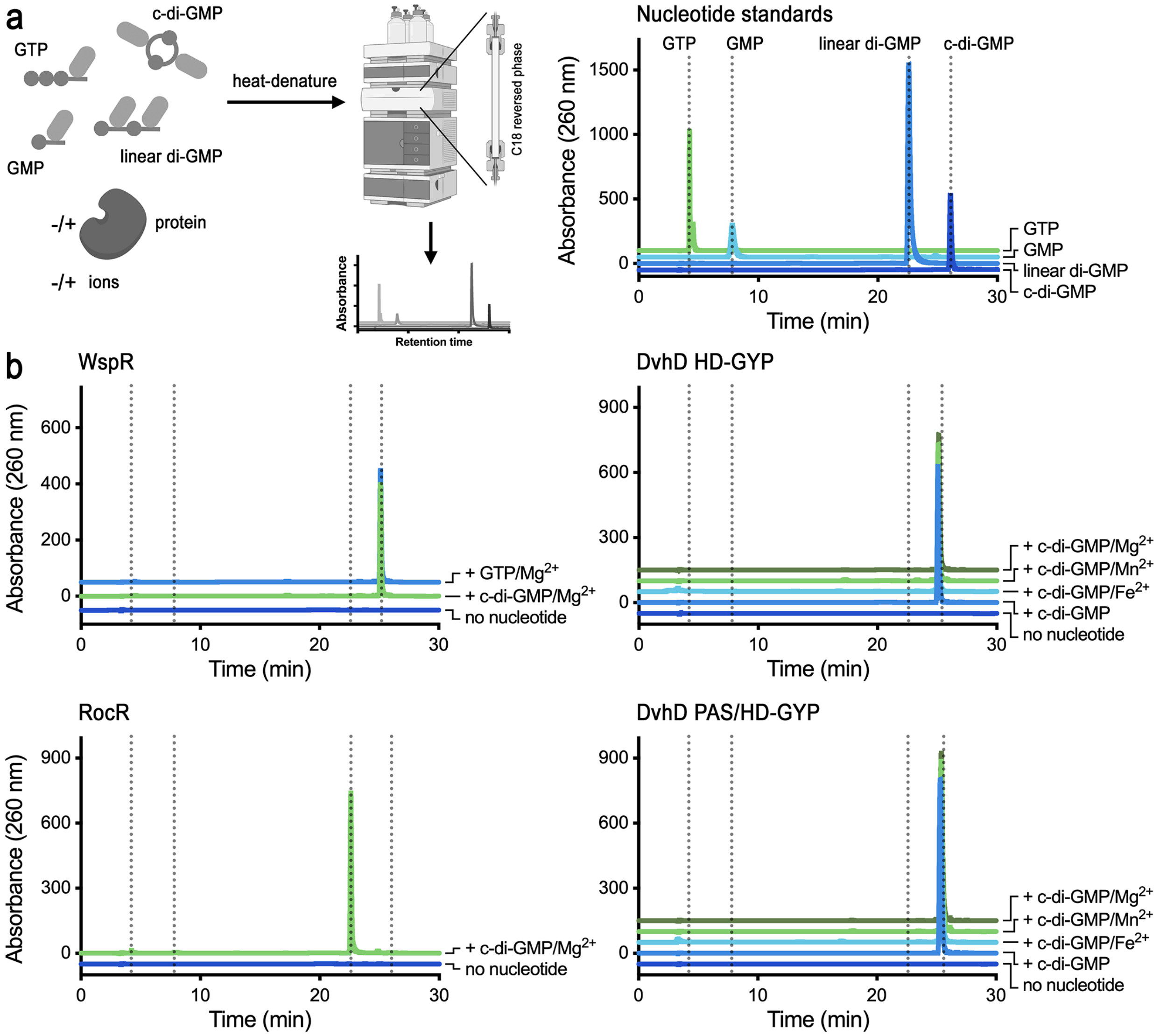

Supplementary Fig. 7. **Assessing catalytic activity of DvhD. a** Assay overview. Purified proteins were incubated in nucleotides in the presence or absence of the indicated metal co-factors. Reactions were heat-denatured, cleared by centrifugation and ultrafiltration, followed by HPLC-based analysis of nucleotide species in the supernatant. Pure nucleotide standards eluted in the following order, from slow to fast: c-di-GMP, linear di-GMP (pGpG), GMP, GTP (right panel). Created in BioRender. Sondermann, H. (2026) https://BioRender.com/hvhmsdz. **b** The diguanylate cyclase WspR and phosphodiesterase RocR were included as controls. Reactions for the HD-GYP and PAS/HD-GYP protein fragments of DvhD are shown with the indicated additives. Representative HPLC elution traces are shown.

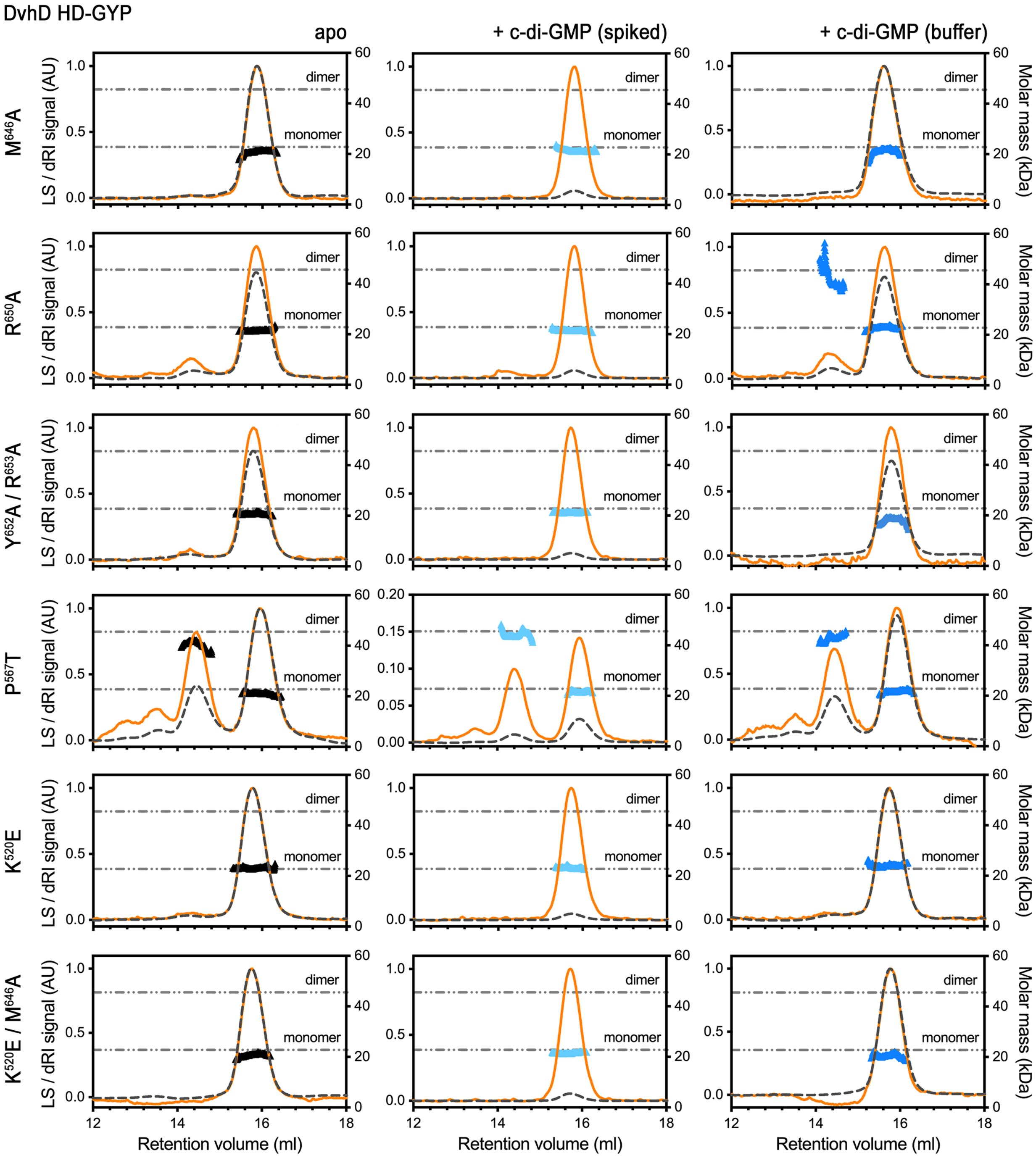

Supplementary Fig. 8. **Effect of targeted mutations introduced in the HD-GYP domain on c-di-GMP-induced dimerization.** SEC-MALS traces and molar mass calculations are shown for the purified HD-GYP domain variants without ligand, with ligand added prior to gel filtrations, or with ligand added to the mobile phase of the gel filtration step. Dashed and solid curves show the light-scattering and refractive index signal, respectively, from a size exclusion-coupled multi-angle light scattering experiment. The data points across the peak report molar mass calculations based on light scattering. Theoretical molecular masses of a monomer and dimer based on protein sequence are indicated as horizontal lines.

Supplementary Table 1. AlphaFold (AF) models and scores.			
AF models	Confidence scores*		
	pLDDT	pTM	ipTM
DvhG deltaTM	64.9	0.59	-
DvhG BTLCP domain	92.0	0.91	-
DvhA 1-108	83.6	0.70	-
DvhA 212-483	85.2	0.79	-
DvhA 2224-2824	82.7	0.52	-
DvhA 2832-3038	89.1	0.87	-
DVU1545 1-90	77.1	0.62	-
DVU1545 144-462	89.8	0.86	-
DVU1545 1871-2414	85.9	0.60	-
*Pfl*_LapG•LapA (88-128)	93.2	0.92	0.87
*Pfl*_LapG•MapA (83-131)	93.5	0.92	0.88
*Vc*_LapG•CraA (100-141)	92.0	0.91	0.84
*Vc*_LapG•FrhA (64-110)	91.3	0.90	0.82
DvhG (210-399)•DvhA (1-110)	89.7	0.89	0.87
DvhG (210-399)•DVU1545 (1-91)	89.4	0.89	0.87
DvhD full-length, dimer	79.7	0.47	0.44
DvhD dCache • DvhG BTLCP **	87.8	0.81	0.84

* Scores for top-ranked models only. pLDDT: averaged value. Scores > 0.8 (high confidence); scores between 0.6 and 0.8 (intermediate confidence); scores < 0.6 (low confidence).
** This model was generated using AF3 using default parameters. All previous models were generated with AF3/ColabFold.

Supplementary Table 2. Quantification of proteolysis data shown in Fig. 4B, plotted as % uncleaved (n=3).

Dilution	DvhA - Ret.		DvhA + Ret		DVU1545 - Ret.		DVU1545 + Ret.	
	Mean	SD	Mean	SD	Mean	SD	Mean	SD
1000	92.2	3.4	39.6	1.7	95.1	1.7	49.3	1.7
500	91.3	1.9	26.8	2.1	92.8	1.6	36.8	1.1
100	77.2	2.0	1.6	0.1	75.3	1.7	2.2	0.6
50	62.9	1.5	1.5	0.3	58.5	2.1	1.5	0.3
10	16.2	1.4	1.6	0.5	9.7	1.1	1.7	1.1
5	4.4	0.7	2.5	1.1	1.9	0.1	1.6	0.7
-DvhG	92.9	1.8	90.5	3.9	95.8	1.0	95.7	0.8

Supplementary Table 3. Quantification of proteolysis data shown in Fig. 4C, plotted as % uncleaved (n=3).

Dilution	DvhA + Ret. (wt)		DvhA + Ret. ($L^{90}E$)		DvhA + Ret. ($I^{85}E$)		DvhA + Ret. ($F^{95}E$)	
	Mean	SD	Mean	SD	Mean	SD	Mean	SD
1600	68.5	0.4	82.4	1.2	88.6	0.5	73.5	0.6
800	50.1	0.9	73.1	1.6	80.2	1.1	57.3	1.3
400	13.7	0.7	45.4	1.2	56.3	1.1	23.8	0.5
200	1.1	0.2	10.8	0.7	18.8	0.8	2.7	0.7
100	1.1	0.2	9.9	0.6	17.7	0.6	2.5	1.0
50	1.4	0.4	1.8	0.2	3.0	0.2	1.1	0.4
-DvhG	97.5	1.1	98.5	0.7	98.1	1.3	99.6	0.2

Supplementary Table 4. Crystallographic data collection and refinement statistics.*			
	DvhD periplasmic domain	DvhD PAS/HD-GYP	DvhD HD-GYP • c-di-GMP
Data collection			
Wavelength	0.9763	1.0332	0.9763
Resolution range [Å]	48.1 - 3.0 (3.07 - 3.00)	46.2 - 3.4 (3.60 - 3.44)	43.8 - 1.8 (1.82 - 1.80)
Space group	P 6_4 2 2	P 6_5 2 2	P 1
Unit cell dimension [Å]	120.4 120.4 124.0	120.9 120.9 98.4	44.9 44.9 115.6
Unit cell angles [º]	90 90 120	90 90 120	89.9 101.4 96.2
Total reflections	425,994 (30,005)	233,863 (25,226)	264,983 (4,737)
Unique reflections	20,145 (1,437)	10,703 (1,338)	137,047 (2,476)
Multiplicity	21.1 (20.9)	21.9 (18.9)	1.9 (1.9)
Completeness (%)	99.9 (99.7)	99.8 (99.4)	84.0 (43.1)
Mean I/sigma(I)	20.5 (1.3)	9.8 (1.4)	9.5 (2.5)
R_{merge}	0.087 (2.646)	0.240 (1.992)	0.048 (0.1649)
CC1/2	1 (0.668)	0.999 (0.520)	0.993 (0.959)
Refinement			
Reflections (refinement)	11,121 (769)	6,007 (723)	70,509 (1,378)
Reflections (R-free)	1,115 (77)	601 (72)	2,009 (41)
R-work	27.00 (36.54)	31.56 (41.73)	19.47 (35.15)
R-free	30.26 (41.69)	32.94 (45.48)	22.86 (40.08)
# non-hydrogen atoms	1,881	2,219	7,217
# macromolecule atoms	1,881	2,219	6,304
# ligand atoms	0	0	276
# solvent atoms	0	0	637
RMS bonds [Å]	0.004	0.004	0.002
RMS angles [º]	0.77	0.75	0.58
Ramachandran favored (%)	94.72	93.75	99.75
Ramachandran allowed (%)	5.28	5.21	0.25
Ramachandran outliers (%)	0.00	1.04	0.00
Average B-factor	133.84	126.70	35.48
* Statistics for the highest-resolution shell are shown in parentheses.			

Supplementary Table 5. ITC data for ligand binding to the PAS/HD-GYP fragment of DvhD (n=3). *

Ligand	Cell (μM)	Syringe (μM)	N (sites)	K_d (μM)	ΔH (kJ/mol)	ΔG (kJ/mol)	-TΔS (kJ/mol)
c-di-GMP	15	350	1.48±0.03	0.169±0.066	-41.3±1.41	-38.2	3.10
c-di-GMP	15	350	1.24±0.02	0.089±0.052	-44.3±1.36	-39.7	4.60
c-di-GMP	15	350	1.01±0.02	0.039±0.026	-53.2±1.61	-41.8	11.50
cGAMP	15	500	1.75±0.01	0.418±0.045	-32.3±0.36	-36.0	-3.67
cGAMP	15	500	1.17±0.04	0.492±0.224	-38.6±2.23	-35.6	3.01
cGAMP	15	350	1.08±0.03	0.594±0.195	-49.5±2.43	-35.1	14.4
pGpG	15	500	0.31±3.39	49.7±93.6	-155±1870	-24.3	131
pGpG	15	666	0.09±0.72	19.8±11.6	-335±2830	-26.5	308
pGpG	15	666	0.16±0.64	28.2±10.7	-335±1440	-25.6	309

* One set of sites model was used.

Supplementary Table 6. Transmembrane topology predictions for DvhG orthologs via DeepTMHMM. *

ProteinID	Pred.	Protein sequence	Detailed prediction
WP_066802140.1	SP	MPGRGIYRTIVVTLCAASIVATLGWAGTAHAASPDKRQLLGTLEFKGSLS	SSSSSSSSSSSSSSSSSSSSSSSSSSSSSSSOOOOOOOOOOOOOOOOOOO
		KLPKWTRVLDKMKAWKGYFNDAATASHPSKQAWNTLKASLKDASELDKLK	OOOOOOOOOOOOOOOOOOOOOOOOOOOOOOOOOOOOOOOOOOOOOOOOOO
		AVTKFFNKWPYRLDKANWGVSEYWATPWEFLKKSGDCEDYSIAKFYGLQE	OOOOOOOOOOOOOOOOOOOOOOOOOOOOOOOOOOOOOOOOOOOOOOOOOO
		LGFSGDQMRIVAVKDAIRGIGHAVLAVYATDDIYILDNQTVMVLSHEKYK	OOOOOOOOOOOOOOOOOOOOOOOOOOOOOOOOOOOOOOOOOOOOOOOOOO
		HYIPQYSVNEKYRWMHVAPKKQTTYEKK	OOOOOOOOOOOOOOOOOOOOOOOOOOOO
WP_156176855.1	SP	MPMDVAHAQSLGDLAKAAKGKGTALFRTVEFRSKSFKALPQWARVLKKSK	SSSSSSSSSSOOOOOOOOOOOOOOOOOOOOOOOOOOOOOOOOOOOOOOOO
		PEIKSFNACANNVANCKTPQQKSWRKIIHAAKNMSRRQKLDTVNKFFNRW	OOOOOOOOOOOOOOOOOOOOOOOOOOOOOOOOOOOOOOOOOOOOOOOOOO
		PYKLDHEVYGVSEYWASPNQFMRTSGDCEDYSIAKFYALKELGFRDNEMR	OOOOOOOOOOOOOOOOOOOOOOOOOOOOOOOOOOOOOOOOOOOOOOOOOO
		IVILKDSIRGIGHAVLAIYEKNDILILDSLSNLILTHTKYRHYIPQYSMN	OOOOOOOOOOOOOOOOOOOOOOOOOOOOOOOOOOOOOOOOOOOOOOOOOO
		STTRWAHIK	OOOOOOOOO
WP_013513847.1	SP	MASATAICRTLWGLWCATLLVLCLVCGGRDALAAGEAAPPRLRLFGTVEF	SSSSSSSSSSSSSSSSSSSSSSSSSSSSSSSSSOOOOOOOOOOOOOOOOO
		KGAITQLPKWTGVLDRMGAWKGFFEDPSMAAIPARQSWQTLKGEIATAAP	OOOOOOOOOOOOOOOOOOOOOOOOOOOOOOOOOOOOOOOOOOOOOOOOOO
		MERVKAVNKFFNQWPYRLDQANYGQSDYWATPPEFMKKSGDCEDYSIAKF	OOOOOOOOOOOOOOOOOOOOOOOOOOOOOOOOOOOOOOOOOOOOOOOOOO
		YALKELGFTGDDMRVVALKDTIRNIGHAVLVVYLDGDAYVLDNQTVMVLS	OOOOOOOOOOOOOOOOOOOOOOOOOOOOOOOOOOOOOOOOOOOOOOOOOO
		HEKYKHYLPQYSVNEKFRWMHVPPTKNTTYTKTKK	OOOOOOOOOOOOOOOOOOOOOOOOOOOOOOOOOOO
WP_231038959.1	SP	MKADGANAADGKGAPRATADTADTADTADTADTADTAPAGTETANTNTVG	SSSSSSSSSSOOOOOOOOOOOOOOOOOOOOOOOOOOOOOOOOOOOOOOOO
		LPDAPLVAQAAQPRQANQPAQTAESAQSAEPRQGAASASRGIRLFNTIEF	OOOOOOOOOOOOOOOOOOOOOOOOOOOOOOOOOOOOOOOOOOOOOOOOOO
		RGPLKHLPKWDRVLNVDRKTPGLIPERALGGRHALWGELRSEWRGLSTMD	OOOOOOOOOOOOOOOOOOOOOOOOOOOOOOOOOOOOOOOOOOOOOOOOOO
		KLRNVNSLFNQWPYRLDNEVWGVVDYWAAPVEFLRKSGDCEDYAITKYFA	OOOOOOOOOOOOOOOOOOOOOOOOOOOOOOOOOOOOOOOOOOOOOOOOOO
		LKQLGVPVSDMRIVILLDSIRRLAHAILVVYTGGDAYVLDNLSNVVLSHQ	OOOOOOOOOOOOOOOOOOOOOOOOOOOOOOOOOOOOOOOOOOOOOOOOOO
		RYGHYVPQYSINEEHRWAHIPIKGGPGARRMK	OOOOOOOOOOOOOOOOOOOOOOOOOOOOOOOO
WP_084264872.1	SP	MMKAFITALGITVILSAPAQSTASPQEPGLFGSLEIASGNLSALPQWQRS	SSSSSSSSSSSSSSSSSSSSSOOOOOOOOOOOOOOOOOOOOOOOOOOOOO
		LAGFDKLREASKRCDEDILKCDSQQMTLWRTKIQELEHSDRSIAMRQINS	OOOOOOOOOOOOOOOOOOOOOOOOOOOOOOOOOOOOOOOOOOOOOOOOOO
		FINKWQHIADQDLYKQSDYWATPLEFITNGGDSEDFAIMKYVSLRELGFS	OOOOOOOOOOOOOOOOOOOOOOOOOOOOOOOOOOOOOOOOOOOOOOOOOO
		ADQMRIVVTNDVLRGVTHTVLSVKLSSKSYILDSQNDTVLREEFVNYYVP	OOOOOOOOOOOOOOOOOOOOOOOOOOOOOOOOOOOOOOOOOOOOOOOOOO
		YYSVNENTRWAHIPSQFSQLGDAAGAKQ	OOOOOOOOOOOOOOOOOOOOOOOOOOOO
WP_018125290.1	SP	MGFAAVISRLTGTGLAFVVALGVLAWCPAANASEQADVESPSSMKEPAPE	SSSSSSSSSSSSSSSSSSSSSSSSSSSSSSSSOOOOOOOOOOOOOOOOOO
		KSEAKKKNRLFGTLEFKGRIKKLPKWSRVLDRMHAWKGYFRDSSLADLPS	OOOOOOOOOOOOOOOOOOOOOOOOOOOOOOOOOOOOOOOOOOOOOOOOOO
		KAGWIKLKADTRGMSRMERLKAVNKFFNRWPYRLDPANYGAKDYWATPPE	OOOOOOOOOOOOOOOOOOOOOOOOOOOOOOOOOOOOOOOOOOOOOOOOOO
		FLKRSGDCEDYAIAKFYALKELGFSGDEMRIVALKDLIRNIGHAVLAVYL	OOOOOOOOOOOOOOOOOOOOOOOOOOOOOOOOOOOOOOOOOOOOOOOOOO
		EDDIYVLDNQTVMVLPHSRYKHYLPYYSVNEKFRWMHVPTSGGDLLKRRL	OOOOOOOOOOOOOOOOOOOOOOOOOOOOOOOOOOOOOOOOOOOOOOOOOO
		KQ	OO
WP_264983481.1	SP	MTGRVGHTLLVLALCAMCIGVVLDPAVEARAADTSPKLLGSLEFKSTIKK	SSSSSSSSSSSSSSSSSSSSSSSSSSSSSSSOOOOOOOOOOOOOOOOOOO
		LPKWTRVLDKMKAWKGYFQDAATANHPSKGSWYALKAELAGKPQKDILKG	OOOOOOOOOOOOOOOOOOOOOOOOOOOOOOOOOOOOOOOOOOOOOOOOOO
		VNKFFNKWPYRLDKANWGVSEYWATPWEFLKKSGDCEDYSIAKFYALQEL	OOOOOOOOOOOOOOOOOOOOOOOOOOOOOOOOOOOOOOOOOOOOOOOOOO
		GFTGDQMRIVAVKDGIRGIGHAILAVFLPDDIYILDNQTIMVLSHKRYKH	OOOOOOOOOOOOOOOOOOOOOOOOOOOOOOOOOOOOOOOOOOOOOOOOOO
		YIPQYSVNEKFRWMHVAPKKKSTYTKAKQ	OOOOOOOOOOOOOOOOOOOOOOOOOOOOO
WP_245590754.1	GLOB	MQAKNEDTAARAGAPVTPEVPATREVRGAVTEATRDAVEPARDGARADTA	OOOOOOOOOOOOOOOOOOOOOOOOOOOOOOOOOOOOOOOOOOOOOOOOOO
		VQATRIVADTPDRPDRPDVADVADVADVAEVGDRPDELPLTPKVDGADAA	OOOOOOOOOOOOOOOOOOOOOOOOOOOOOOOOOOOOOOOOOOOOOOOOOO
		DGKGTPPVAVDTADTAETAETADTETAETAPVGLPDAPLVAQAEQARQAR	OOOOOOOOOOOOOOOOOOOOOOOOOOOOOOOOOOOOOOOOOOOOOOOOOO
		QAGQARQAGQAGQAGQAGQAAESAQAAEPRQGTSSASRGIRLFNTIEFRG	OOOOOOOOOOOOOOOOOOOOOOOOOOOOOOOOOOOOOOOOOOOOOOOOOO
		PLKNLPKWDRVLNVDRKTPGLIPERALGGRNALWGALKAEWRDLSTMDKL	OOOOOOOOOOOOOOOOOOOOOOOOOOOOOOOOOOOOOOOOOOOOOOOOOO
		RNVNSLFNQWPYRLDSEVWGVVDYWAAPIEFLRKSGDCEDYAITKYFALK	OOOOOOOOOOOOOOOOOOOOOOOOOOOOOOOOOOOOOOOOOOOOOOOOOO
		QLGVPASDMRIVILLDSIRRLAHAILVVYTGGDAYVLDNLSNVVLSHKRY	OOOOOOOOOOOOOOOOOOOOOOOOOOOOOOOOOOOOOOOOOOOOOOOOOO
		GHYVPQYSINEQYRWAHIPTRGGPGTRRMQ	OOOOOOOOOOOOOOOOOOOOOOOOOOOOOO

WP_176244911.1	SP	MARGRGAARLRALAFAGLLAAGTAVGTVGAAAGATGTGDTTILFGSRATA	SSSSSSSSSSSSSSSSSSSSSSSSSSSSSSSSSOOOOOOOOOOOOOOOOO
		HQGLKPFPRWTGAIERYFEARKDRAGSCDDPTFNRCHYERWQALIRDVAG	OOOOOOOOOOOOOOOOOOOOOOOOOOOOOOOOOOOOOOOOOOOOOOOOOO
		KPRLRQLEAVNDFMNRYRYILDPINWGVKDYWASPDEFFRRFGDCEDYAI	OOOOOOOOOOOOOOOOOOOOOOOOOOOOOOOOOOOOOOOOOOOOOOOOOO
		AKYFSLRAIGVPADDLRIVVLQDLNLGIPHAVLTVREGDKIYVLDNQISM	OOOOOOOOOOOOOOOOOOOOOOOOOOOOOOOOOOOOOOOOOOOOOOOOOO
		VVEAGRIRHYRPIYSVNENGWWRYRPAG	OOOOOOOOOOOOOOOOOOOOOOOOOOOO
WP_236031536.1	SP	MDQAAPDNPAAVRPQSAGPPQSRADAVASLPAVDQESSPAAPAKPSAPPH	SSSSSSSSSSSSSSSSSSOOOOOOOOOOOOOOOOOOOOOOOOOOOOOOOO
		TTLRLFGTVEFRRPLSSLPGWLELLKRNAQHGIFTPGQVLKKGVTWAQFR	OOOOOOOOOOOOOOOOOOOOOOOOOOOOOOOOOOOOOOOOOOOOOOOOOO
		AGAAGKKGVSLLRYVNAFWNAWPYREDRDVWGREDYWAIPQEFLRRSGDC	OOOOOOOOOOOOOOOOOOOOOOOOOOOOOOOOOOOOOOOOOOOOOOOOOO
		EDYAIIKYFTLKELGLSPKTMRIVVVRDTVRNLGHAVLAVYMDGTAYILD	OOOOOOOOOOOOOOOOOOOOOOOOOOOOOOOOOOOOOOOOOOOOOOOOOO
		NLSNAILPHDRLRQYRPQYSVNEQGRWAHLQVSRVP	OOOOOOOOOOOOOOOOOOOOOOOOOOOOOOOOOOOO
WP_092379318.1	GLOB	MQNQTSQVELASEDAGAATGGTHQETRQDSGSSRIADPLKTQANIRPDQE	OOOOOOOOOOOOOOOOOOOOOOOOOOOOOOOOOOOOOOOOOOOOOOOOOO
		QAKDATPAVSLKQGEILDSQASDSLEQAPGTPAAVQGRDSNVTTVAQAER	OOOOOOOOOOOOOOOOOOOOOOOOOOOOOOOOOOOOOOOOOOOOOOOOOO
		NQESSTSKAPLPVWRQGAQTQAPTPSGQAPRAQSNTGDQPIRLFQSAAFR	OOOOOOOOOOOOOOOOOOOOOOOOOOOOOOOOOOOOOOOOOOOOOOOOOO
		GNFNALPKWKRILSKVKGQLQTLGSCTSAKTCPPGATSWQRIIKQARGKE	OOOOOOOOOOOOOOOOOOOOOOOOOOOOOOOOOOOOOOOOOOOOOOOOOO
		RMEQLKMINSFFNKWPYRLDQDAYGVSDWWATPQEFLKISGDCEDYAIIK	OOOOOOOOOOOOOOOOOOOOOOOOOOOOOOOOOOOOOOOOOOOOOOOOOO
		YFALRELGFSKDELRIVVVKDRIRGIAHAVLAVFTDGDAYILNNISDAIF	OOOOOOOOOOOOOOOOOOOOOOOOOOOOOOOOOOOOOOOOOOOOOOOOOO
		THGKYRHYIPQYSLNEEHRWSHIPVAN	OOOOOOOOOOOOOOOOOOOOOOOOOOO
WP_246118364.1	GLOB	MNELEPIFVNGKVFTRTATWDSFKQGARGKSGMELLRYVNTFWNRFPYRE	OOOOOOOOOOOOOOOOOOOOOOOOOOOOOOOOOOOOOOOOOOOOOOOOOO
		DITNWGKADYWVWPNLFLKKSGDCEDYAIVKYFTLKELGMDTDKLRIVVL	OOOOOOOOOOOOOOOOOOOOOOOOOOOOOOOOOOOOOOOOOOOOOOOOOO
		RDTLRRLSHAVLAVYMDNDIVILDNISNAILPQGRLRHYVPQFSFNEKAR	OOOOOOOOOOOOOOOOOOOOOOOOOOOOOOOOOOOOOOOOOOOOOOOOOO
		WAHMRGKKTNE	OOOOOOOOOOO
WP_006000675.1	SP	MPGWNNIRALILLGLLLSTVGCSPVQASSPKTTAADRPPPTGVFDSYEFR	SSSSSSSSSSSSSSSSSSSSSOOOOOOOOOOOOOOOOOOOOOOOOOOOOO
		GTLTALKNWQRVLPLGDDEMNRFSSPQQDTGIRAAREWQQVVTQLTDAPL	OOOOOOOOOOOOOOOOOOOOOOOOOOOOOOOOOOOOOOOOOOOOOOOOOO
		MEQLKTVNRFFNRWPYRLDREVWQKKDYWATPLEFLRHSGDCEDYAICKY	OOOOOOOOOOOOOOOOOOOOOOOOOOOOOOOOOOOOOOOOOOOOOOOOOO
		FALRHLGVAPDRMRIVILHDTIRTITHAVLAVYTQNDILILDNLSDPVFS	OOOOOOOOOOOOOOOOOOOOOOOOOOOOOOOOOOOOOOOOOOOOOOOOOO
		HHRYQHYLPQYSVNENTRWIHIKPRSTALPTRTDTGE	OOOOOOOOOOOOOOOOOOOOOOOOOOOOOOOOOOOOO
WP_155934525.1	SP	MVHTARICRTSWGWCVALLVLSLTVRAGACLAAEGGASASRLFGTVEFKG	SSSSSSSSSSSSSSSSSSSSSSSSSSSSSSSSOOOOOOOOOOOOOOOOOO
		PITQLPKWTGVLDKMKGWKGFFEDPSMAGVPARQSWQTLKSEIASAAPMD	OOOOOOOOOOOOOOOOOOOOOOOOOOOOOOOOOOOOOOOOOOOOOOOOOO
		RVKAVNKYFNQWPYRLDQANYGQSDYWATPPEFMKKSGDCEDYSIAKFYA	OOOOOOOOOOOOOOOOOOOOOOOOOOOOOOOOOOOOOOOOOOOOOOOOOO
		LKELGFTGDDMRIVALRDTIRNIGHAVLVVYLDGDAYVLDNQTVMVLSHE	OOOOOOOOOOOOOOOOOOOOOOOOOOOOOOOOOOOOOOOOOOOOOOOOOO
		KYKHYLPQYSVNEQFRWMHVPPTGSTTYTKQKK	OOOOOOOOOOOOOOOOOOOOOOOOOOOOOOOOO
WP_169726987.1	SP	MLFFSVLGMAPGNVWAANLSLENHQDILGLDQETPDTPVAKKPRTPASAV	SSSSSSSSSSSSSSSSOOOOOOOOOOOOOOOOOOOOOOOOOOOOOOOOOO
		GDKAGEGMVPPQVEEEVEETTPASEPDPKAEAPADKPKPIHLFGHVEFRG	OOOOOOOOOOOOOOOOOOOOOOOOOOOOOOOOOOOOOOOOOOOOOOOOOO
		PLKNIPKWERVLKEEQKAPSYGAGNSTGMPEEVRKRWLPMREKLMGQPLM	OOOOOOOOOOOOOOOOOOOOOOOOOOOOOOOOOOOOOOOOOOOOOOOOOO
		DQLKAVNNFFNQWPYKNDIANWGLEDYWATPKEFFKKSGDCEDYAIAKYY	OOOOOOOOOOOOOOOOOOOOOOOOOOOOOOOOOOOOOOOOOOOOOOOOOO
		ALRDLGVPTHKMRIVAVMETIRGIGHAVLVVFVDDKAYVLDSLSNLLLQH	OOOOOOOOOOOOOOOOOOOOOOOOOOOOOOOOOOOOOOOOOOOOOOOOOO
		SRLTNYAPKFSVNEEFRWVHVKPVQQPKKK	OOOOOOOOOOOOOOOOOOOOOOOOOOOOOO
WP_154510627.1	SP	MPPFLQHGGPWQRRLRGGVFALAVAVVCLLPVVGCFGFAADITPGAVPGG	SSSSSSSSSSSSSSSSSSSSSSSSSSSSSSSSSSOOOOOOOOOOOOOOOO
		ESGVSERPRVLERVDESAFSAAPVSTPEDAAPALSASDAATPDASGPSDP	OOOOOOOOOOOOOOOOOOOOOOOOOOOOOOOOOOOOOOOOOOOOOOOOOO
		EGAAAARVGRPVSADTIGPSPAVLPDKDDLPSAEASASPTAAVAVPKIKL	OOOOOOOOOOOOOOOOOOOOOOOOOOOOOOOOOOOOOOOOOOOOOOOOOO
		FGTVEFKRPLSSLPAWLDLLKRNQADPIFIPEKHFNKKVTWAQFKEKAKG	OOOOOOOOOOOOOOOOOOOOOOOOOOOOOOOOOOOOOOOOOOOOOOOOOO
		KNPMNLLRYVNSFWNTWPYREDIENWGQADYWEIPAEFLKKSGDCEDYSI	OOOOOOOOOOOOOOOOOOOOOOOOOOOOOOOOOOOOOOOOOOOOOOOOOO
		IKYFTLKELGIPPETMRIVVVRDTIRNMAHAVLVVYMNNDAYVLDSLSNV	OOOOOOOOOOOOOOOOOOOOOOOOOOOOOOOOOOOOOOOOOOOOOOOOOO
		VLSHTRFGHYSPQYSVNEFGRWAHLKGRKLK	OOOOOOOOOOOOOOOOOOOOOOOOOOOOOOO
WP_193371556.1	SP	MDRRAFLRHSAAVAGAAASGLLVDAWRNDAGAQSLQDLMKTAPNAGTALF	SSSSSSSSSSSSSSSSSSSSSSSSSSSSSSSSOOOOOOOOOOOOOOOOOO
		GSTEVSAANLKALPQWARVLAKMKSERKLFRACLDNQAACTTAGLRSWRE	OOOOOOOOOOOOOOOOOOOOOOOOOOOOOOOOOOOOOOOOOOOOOOOOOO
		VAAEAKGKPQLEMLKVVNRYFNRWPYKIDRELYGVSEFWATPTEFMKRSG	OOOOOOOOOOOOOOOOOOOOOOOOOOOOOOOOOOOOOOOOOOOOOOOOOO
		DCEDYSIAKFFALRDLGMANEDLRVVILMDRIRRLGHAVLAVYALGDILI	OOOOOOOOOOOOOOOOOOOOOOOOOOOOOOOOOOOOOOOOOOOOOOOOOO
		LDSLTDLVFSHKKYKHYVPQYSMNETTRWAHFYGKNRTASL	OOOOOOOOOOOOOOOOOOOOOOOOOOOOOOOOOOOOOOOOO

WP_169543093.1	SP	MTCTFACSLMMVPHIPSAQAVGIFGFKEYAHDDLKPFPKWKNVLETYKDT	SSSSSSSSSSSSSSSSSSSSOOOOOOOOOOOOOOOOOOOOOOOOOOOOOO
		PGDCDNGQLNACAYAKWEELVEGLKGKPKKYQLERVNTYINLHRYILDPI	OOOOOOOOOOOOOOOOOOOOOOOOOOOOOOOOOOOOOOOOOOOOOOOOOO
		NWGVKDYWEIPKEFFARFGDCEDYAIVKYFTLRSLGWKAEDMKIVVLQDL	OOOOOOOOOOOOOOOOOOOOOOOOOOOOOOOOOOOOOOOOOOOOOOOOOO
		NLRIAHAVLAVQFDKKEVVLDNQIGLVIDAKRIRHYRPIYSVNEQGWWRY	OOOOOOOOOOOOOOOOOOOOOOOOOOOOOOOOOOOOOOOOOOOOOOOOOO
		KP	OO
WP_085901370.1	SP	MTDFKPLQIIKKAALIASLASVLVNTMPMDAAHAQSLGDLAKAAKGKGTA	SSSSSSSSSSSSSSSSSSSSSSSSSSSSSSSSSSOOOOOOOOOOOOOOOO
		LFRTVEFRSKSFKALPQWARVLKKSKPEIRSFNACANNIANCKTPQQKSW	OOOOOOOOOOOOOOOOOOOOOOOOOOOOOOOOOOOOOOOOOOOOOOOOOO
		RKIIHAAKNMSRRQKLDTVNKFFNRWPYKLDHEVYGVSEYWASPNQFMRT	OOOOOOOOOOOOOOOOOOOOOOOOOOOOOOOOOOOOOOOOOOOOOOOOOO
		SGDCEDYSIAKFYALKELGFKDNEMRIVILKDSIRGIGHAVLAIYEKNDI	OOOOOOOOOOOOOOOOOOOOOOOOOOOOOOOOOOOOOOOOOOOOOOOOOO
		LILDSLSNLILTHTKYRHYIPQYSMNSTTRWAHIK	OOOOOOOOOOOOOOOOOOOOOOOOOOOOOOOOOOO
WP_174408948.1	SP	MSKDAPGTPLPERWPQCRPARLAVLLLCIGIASVHTAGGWRAFAQDKQAA	SSSSSSSSSSSSSSSSSSSSSSSSSSSSSSSSSSSSSSSSSSSSOOOOOO
		SAILGASAGQTNATQKPSAFRQLLSGTQAAPPAPATGAKADEATAGGSIR	OOOOOOOOOOOOOOOOOOOOOOOOOOOOOOOOOOOOOOOOOOOOOOOOOO
		LFNTVEFKGPIKSLPQWTRVMQEMVKAGGTLAKALPRSEQEGGKGDARAA	OOOOOOOOOOOOOOOOOOOOOOOOOOOOOOOOOOOOOOOOOOOOOOOOOO
		WERFVASVAKEEPMEKIRRTNTYFNQFPYRLDEEVWGQADYWATPAEFIS	OOOOOOOOOOOOOOOOOOOOOOOOOOOOOOOOOOOOOOOOOOOOOOOOOO
		RSGDCEDFSIIKYYALKELGIDPQHMRIVAVKDRIRGIGHAVLVVFMQNN	OOOOOOOOOOOOOOOOOOOOOOOOOOOOOOOOOOOOOOOOOOOOOOOOOO
		AWVLDNQTDLVLGHDRYGHYLPQYSVNESYRWAHVPAQ	OOOOOOOOOOOOOOOOOOOOOOOOOOOOOOOOOOOOOO
WP_223294588.1	GLOB	MRLFGTIEFRGQLKALPKWSRVVETERKKPGLYLDRALGGKGGQVWRELR	OOOOOOOOOOOOOOOOOOOOOOOOOOOOOOOOOOOOOOOOOOOOOOOOOO
		GEWQGLPLMERLKKVNTFFNQWPYRLDSENYGLPDYWATPDEFLRKSGDC	OOOOOOOOOOOOOOOOOOOOOOOOOOOOOOOOOOOOOOOOOOOOOOOOOO
		EDYSIIKYFALKQLGVSADSMRIVVLLDKIRGIAHAVLAVYDGNTAYILD	OOOOOOOOOOOOOOOOOOOOOOOOOOOOOOOOOOOOOOOOOOOOOOOOOO
		NLSGLVLAHDFYKHYVPQYSVNESYRWAHIPLGKKAGRK	OOOOOOOOOOOOOOOOOOOOOOOOOOOOOOOOOOOOOOO
WP_012805218.1	GLOB	MHDQPAQTKHARADTNAGTMISARPEQEHNATRSASPQEMQTDVQPDQKP	OOOOOOOOOOOOOOOOOOOOOOOOOOOOOOOOOOOOOOOOOOOOOOOOOO
		DQVADPHVQTEQSQPPDTQASVPAEQAPVTEADAALAQDLDSNAPEGTQA	OOOOOOOOOOOOOOOOOOOOOOOOOOOOOOOOOOOOOOOOOOOOOOOOOO
		QQNQDSPTQRAALPVWRQGTQTQPAAPLEQARSAGADTGGQPLRLFQSAA	OOOOOOOOOOOOOOOOOOOOOOOOOOOOOOOOOOOOOOOOOOOOOOOOOO
		FRGNFNALPKWKRILSKVKGQIQTLSSCTSATACPPGATSWQRIMKQARG	OOOOOOOOOOOOOOOOOOOOOOOOOOOOOOOOOOOOOOOOOOOOOOOOOO
		KERMEQLKMINSFFNKWPYRLDQDAYGVSDWWATPQEFLKISGDCEDYAI	OOOOOOOOOOOOOOOOOOOOOOOOOOOOOOOOOOOOOOOOOOOOOOOOOO
		IKYFALRELGFSQNELRIVVLKDRIRGIAHAVLAVFTHGDAYILNNISDA	OOOOOOOOOOOOOOOOOOOOOOOOOOOOOOOOOOOOOOOOOOOOOOOOOO
		IFTHDKYRHYVPQYSLNEEHRWSHIPVAN	OOOOOOOOOOOOOOOOOOOOOOOOOOOOO
WP_161949025.1	GLOB	MQNQTGQVELASRDANAATGGTSQETRQDNESSRMPSPLKTQADIGPDQE	OOOOOOOOOOOOOOOOOOOOOOOOOOOOOOOOOOOOOOOOOOOOOOOOOO
		RAKDATPAVSRKQGEALDSQTSVASEQAPDTPATVAKGDRNQESSTPKAP	OOOOOOOOOOOOOOOOOOOOOOOOOOOOOOOOOOOOOOOOOOOOOOOOOO
		LPVWRQGAQAQAPTPSGQAPRTKSETGDQPIRLFQSAAFRGNFNALPKWK	OOOOOOOOOOOOOOOOOOOOOOOOOOOOOOOOOOOOOOOOOOOOOOOOOO
		RVLSKVKGQIQTLGSCTSAKTCPPGATSWQRIMKQARGKERTEQLKMINS	OOOOOOOOOOOOOOOOOOOOOOOOOOOOOOOOOOOOOOOOOOOOOOOOOO
		FFNKWPYRLDQDAYGASDWWATPQEFLKISGDCEDYAIIKYFALRELGFS	OOOOOOOOOOOOOOOOOOOOOOOOOOOOOOOOOOOOOOOOOOOOOOOOOO
		KDELRIVVVKDRIRGIAHAVLAVFTDGDAYILNNISDAIFTHGKYRHYIP	OOOOOOOOOOOOOOOOOOOOOOOOOOOOOOOOOOOOOOOOOOOOOOOOOO
		QYSLNEEHRWSHIPVAN	OOOOOOOOOOOOOOOOO
WP_236884816.1	SP	MPVLAALLVAGLFFPARAWPDMASMRLFQSASFRGNFDALPKWKRVLKKA	SSSSSSSSSSSSSSSSSSOOOOOOOOOOOOOOOOOOOOOOOOOOOOOOOO
		EGEIRRFQTCSGQCPPGASSWRRIMARARDRQPLEQIRMVNEFFNKWPYR	OOOOOOOOOOOOOOOOOOOOOOOOOOOOOOOOOOOOOOOOOOOOOOOOOO
		LDEEAYGVSDWWATPQEFLKISGDCEDYAIIKYFALRELGFAPGALRIVV	OOOOOOOOOOOOOOOOOOOOOOOOOOOOOOOOOOOOOOOOOOOOOOOOOO
		LKDRIRNIGHAVLAVFLDGEVHVLDNMTDMVFPHTKYRHYVPQYSLNETT	OOOOOOOOOOOOOOOOOOOOOOOOOOOOOOOOOOOOOOOOOOOOOOOOOO
		RWNHIPLAKKP	OOOOOOOOOOO
WP_281762967.1	SP	MTMTALCAVFIVVVFGMAPTASAAAKKQKQPRLLGTMEFKGKIQKLPKWS	SSSSSSSSSSSSSSSSSSSSSSSOOOOOOOOOOOOOOOOOOOOOOOOOOO
		RVVTKMQAWKGYFQDSKTANHPSKSGWYALKSQIKGKPNKEKLKAVTKFF	OOOOOOOOOOOOOOOOOOOOOOOOOOOOOOOOOOOOOOOOOOOOOOOOOO
		NKWPYRLDKANWGVSEYWATPWEFLRKSGDCEDYSIAKFYALQEVGFSQD	OOOOOOOOOOOOOOOOOOOOOOOOOOOOOOOOOOOOOOOOOOOOOOOOOO
		QMRIVAVKDAIRGIGHAVLAVYTDNDIFILDNQTVMVLSHTKYKHYIPQY	OOOOOOOOOOOOOOOOOOOOOOOOOOOOOOOOOOOOOOOOOOOOOOOOOO
		SVNEKFRWMHVAPKKKSTYTKTKK	OOOOOOOOOOOOOOOOOOOOOOOO
WP_005027457.1	SP	MILWRWLRVLAGLLAVSIFVGLPYPGAFASDDDDEILLKRSSVGSLLSNR	SSSSSSSSSSSSSSSSSSSSSSSSSSSSSOOOOOOOOOOOOOOOOOOOOO
		NKAEGARVAAPSDPPPAVTPAEPAREPEAQAAPAGKRGEGGPIRLFGTLE	OOOOOOOOOOOOOOOOOOOOOOOOOOOOOOOOOOOOOOOOOOOOOOOOOO
		MRSKIGKMPKWTSVLEKERKNPGYVANRQFPGQGAWKDIKAKLSDMSPLE	OOOOOOOOOOOOOOOOOOOOOOOOOOOOOOOOOOOOOOOOOOOOOOOOOO
		QVKAVNVLINRWPYRTDMDVWGVMDYWETPVEFFQKSGDCEDFAIAKYFA	OOOOOOOOOOOOOOOOOOOOOOOOOOOOOOOOOOOOOOOOOOOOOOOOOO
		LRDLGFPASQMRIVVLKDTLRNLDHAVTAVYLDGDAWILDNLSNAVLSHK	OOOOOOOOOOOOOOOOOOOOOOOOOOOOOOOOOOOOOOOOOOOOOOOOOO
		RLSHYRPQFSVNEEYRWAHLTPSAPKKTGR	OOOOOOOOOOOOOOOOOOOOOOOOOOOOOO

WP_097012996.1	SP	MTERSSILHPAAMLVCVACIVVCLCCGPAAAADSAPPKVKLFGTVEFKGK	SSSSSSSSSSSSSSSSSSSSSSSSSSSSSSSOOOOOOOOOOOOOOOOOOO
		IKQLPNWTRVLGKMEKWKGFFQYISTAKLPSKSGWSKLVKEVEGQDAMGK	OOOOOOOOOOOOOOOOOOOOOOOOOOOOOOOOOOOOOOOOOOOOOOOOOO
		LKAVNKFFNLWPYRLDAANYGVSDYWATPMEFLKKSGDCEDYAIAKYYAL	OOOOOOOOOOOOOOOOOOOOOOOOOOOOOOOOOOOOOOOOOOOOOOOOOO
		QEFGFSIDSMRIVAVKDYIRGIGHAVLVVYTDSGAYVLDNQTNMVLSHAR	OOOOOOOOOOOOOOOOOOOOOOOOOOOOOOOOOOOOOOOOOOOOOOOOOO
		YKHYIPQYSVNEKFRWMHVPTKKRFGKKATTNNVKQENVLPVP	OOOOOOOOOOOOOOOOOOOOOOOOOOOOOOOOOOOOOOOOOOO
WP_174406921.1	TM	MKKNVRHTLHSAHRTGSGQAPVPAVPLEPDARPAKPEQADRKLGAGRTGQ	IIIIIIIIIIIIIIIIIIIIIIIIIIIIIIIIIIIIIIIIIIIIIIIIII
		AGGTGKSGWSLACLSLVAALIPAVILAPAGGLWRAHAQEEPSMAAKMLEG	IIIIIIIIIIIIIIIIMMMMMMMMMMOOOOOOOOOOOOOOOOOOOOOOOO
		AKALPAQQRPSAFRQLITGTQQPAPAPAGKTGQNGRVRLFNTVEFKGPLK	OOOOOOOOOOOOOOOOOOOOOOOOOOOOOOOOOOOOOOOOOOOOOOOOOO
		LVPQWSRVMQAMKAGKDKLSAVLPATGNGNARTAWKALRDSAAKDPLMEK	OOOOOOOOOOOOOOOOOOOOOOOOOOOOOOOOOOOOOOOOOOOOOOOOOO
		LKKVNAYFNKYPYRLDQEIWGKTDYWATPAEFAEKSGDCEDYAISKYYAL	OOOOOOOOOOOOOOOOOOOOOOOOOOOOOOOOOOOOOOOOOOOOOOOOOO
		REMGIEADKLRIVALRDRIRGIGHAVLVVYAEGTAWVLDNQTDLVLSHDR	OOOOOOOOOOOOOOOOOOOOOOOOOOOOOOOOOOOOOOOOOOOOOOOOOO
		YSHYQPQYSVNEHNRWAHVPTR	OOOOOOOOOOOOOOOOOOOOOO
WP_183719708.1	SP	MKTNRLLRFSLITALPLAVVFLLVALTARYFLPLPATAAQNTASGIVAGG	SSSSSSSSSSSSSSSSSSSSSSSSSSSSSSSSSSSSSSOOOOOOOOOOOO
		LVGDDYAPERPISTTLDKVDQIQPSSLEGFTRQPPAPASNKQQTPRPGKK	OOOOOOOOOOOOOOOOOOOOOOOOOOOOOOOOOOOOOOOOOOOOOOOOOO
		NAPSNQEGVGQPNESRDALQDSENTARQDDGKASPQDQGNAAPEDEGDDV	OOOOOOOOOOOOOOOOOOOOOOOOOOOOOOOOOOOOOOOOOOOOOOOOOO
		VPASHDKVDVPTADSASATSSSDFSKEQVGTSGNKVQLFGTVEFKRPLSS	OOOOOOOOOOOOOOOOOOOOOOOOOOOOOOOOOOOOOOOOOOOOOOOOOO
		LPGWLTLLKRNAKDPIFIPGKYFKKSVTWDNFKEKAKGKSPMDLLRYVNS	OOOOOOOOOOOOOOOOOOOOOOOOOOOOOOOOOOOOOOOOOOOOOOOOOO
		FWNTWPYKEDIVNWGVEDYWAIPAEFLKKSGDCEDYAIVKYFTLKELGIP	OOOOOOOOOOOOOOOOOOOOOOOOOOOOOOOOOOOOOOOOOOOOOOOOOO
		AENMRIVVVRDTVRNLAHAVLAVYLNGDAYILDNLSNAVLSHSRIRQYSP	OOOOOOOOOOOOOOOOOOOOOOOOOOOOOOOOOOOOOOOOOOOOOOOOOO
		QYSVNEVGRWAHLKGRKVK	OOOOOOOOOOOOOOOOOOO
WP_147818644.1	SP	MAAPSFIPRAARGLLYAVLVAACLAVWSASGVRAADKAQPPRLFNTLEFK	SSSSSSSSSSSSSSSSSSSSSSSSSSSSSSSSSSOOOOOOOOOOOOOOOO
		GKIKKLPKWMGVLRRMALWRGYFKDSSMDRLPSKAGWLKLKKSVAGKPPL	OOOOOOOOOOOOOOOOOOOOOOOOOOOOOOOOOOOOOOOOOOOOOOOOOO
		ERLKAVNRYFNQWPYRLDMSNYGQRDYWATPVEFLKKSGDCEDYAIAKYY	OOOOOOOOOOOOOOOOOOOOOOOOOOOOOOOOOOOOOOOOOOOOOOOOOO
		ALKELGFSPGQMRIVAVRDMIRNIGHAVLAVYLPDDIYILDNQTVMVLSH	OOOOOOOOOOOOOOOOOOOOOOOOOOOOOOOOOOOOOOOOOOOOOOOOOO
		KRYTHYVPQYSVNERFRWMHVPPMKKKKTQAAPTARVQDRNNSDNGTLTA	OOOOOOOOOOOOOOOOOOOOOOOOOOOOOOOOOOOOOOOOOOOOOOOOOO
		REPEKNNQME	OOOOOOOOOO
WP_077072032.1	SP	MGIAAHMAYVGRTLALLLCVACPVGYVPLFAASAEAKPRLFGTVEFQMDL	SSSSSSSSSSSSSSSSSSSSSSSSSSSSSSSSSSSSOOOOOOOOOOOOOO
		KKQKNWLSAMERNGKNPIFFDENRFNSSTLWKDLKKRAEGRPLREQLDII	OOOOOOOOOOOOOOOOOOOOOOOOOOOOOOOOOOOOOOOOOOOOOOOOOO
		NRFWNKWPYRTDQEAYRKPDYWAAPYEFLKLSGDCEDYSIVKYYTLKALG	OOOOOOOOOOOOOOOOOOOOOOOOOOOOOOOOOOOOOOOOOOOOOOOOOO
		VPVDDMRIVVVRETIRNIGHAVLAVYDGDDIYILDNLSETVRPAQRVRNY	OOOOOOOOOOOOOOOOOOOOOOOOOOOOOOOOOOOOOOOOOOOOOOOOOO
		APQFSVNEKYRWVHVKPR	OOOOOOOOOOOOOOOOOO
WP_245628634.1	SP	MRIRRAAQRTFITALAVVCFACLAPRAHAQDSHDKPPELFGTMEFKGTIT	SSSSSSSSSSSSSSSSSSSSSSSSSSSSSOOOOOOOOOOOOOOOOOOOOO
		RLPKWTGVLQKMRLWKGYFQGAKTSGLPSRSGWQKLKDQVSDKPPMEKLK	OOOOOOOOOOOOOOOOOOOOOOOOOOOOOOOOOOOOOOOOOOOOOOOOOO
		AVNRFFNQWPYRLDKANYGRSDYWATPFEFLKKSGDCEDYAIAKYYALKE	OOOOOOOOOOOOOOOOOOOOOOOOOOOOOOOOOOOOOOOOOOOOOOOOOO
		LGFSVEDMRIVALKDMIRNIGHAVLAVYLDGEIFVLDNQTVMVLPHDRYT	OOOOOOOOOOOOOOOOOOOOOOOOOOOOOOOOOOOOOOOOOOOOOOOOOO
		HYVPHYSVNEKYRWMHVPRTKLDD	OOOOOOOOOOOOOOOOOOOOOOOO
WP_015751021.1	SP	MGAQQQPQRIVSLLTQHIVWVLLLLGVLGLADTAGAAPKTAGRVLGALSF	SSSSSSSSSSSSSSSSSSSSSSSSSSSSSSSSSSSSOOOOOOOOOOOOOO
		RGSLSALPQWKRVVREAPQEVSLLGNCTVGAQGCPDAATTWQALLEQGND	OOOOOOOOOOOOOOOOOOOOOOOOOOOOOOOOOOOOOOOOOOOOOOOOOO
		LSERQQLQKVNARLNRWPYRMDPEAYGVFDYWATPLEFLRQSGDCEDFCI	OOOOOOOOOOOOOOOOOOOOOOOOOOOOOOOOOOOOOOOOOOOOOOOOOO
		VKYYALREMGFAVERLRIVVVRDTIRNVTHAVLRVQLADKAVILDNLSDL	OOOOOOOOOOOOOOOOOOOOOOOOOOOOOOOOOOOOOOOOOOOOOOOOOO
		VVSQDKYGHYHPQYSVNEHYRWAHVIPRQHSPTSLSTLRPR	OOOOOOOOOOOOOOOOOOOOOOOOOOOOOOOOOOOOOOOOO
WP_251935991.1	SP	MRSFSRFSCLSIVTLSATLSLAPMKSAYADVNVDGLFGTVEIASDNLSAL	SSSSSSSSSSSSSSSSSSSSSSSSSSSSSOOOOOOOOOOOOOOOOOOOOO
		PQWKRVIESFEEVAIEAKKCDLKIEDCYSQQMTLWRAKINELRTSDKRVQ	OOOOOOOOOOOOOOOOOOOOOOOOOOOOOOOOOOOOOOOOOOOOOOOOOO
		IQQINRFLNSWRKRTDTENYAVSDYWATPLEFMENGGDSEDFAIMKYISL	OOOOOOOOOOOOOOOOOOOOOOOOOOOOOOOOOOOOOOOOOOOOOOOOOO
		KELGVPPSDMRIVVTNDVLRNNVHTVLSVKTGGKIYILDDLNDSILSESF	OOOOOOOOOOOOOOOOOOOOOOOOOOOOOOOOOOOOOOOOOOOOOOOOOO
		TPYYLPLYSVNEQNRWAHIPKAVSVEQASQGTTQND	OOOOOOOOOOOOOOOOOOOOOOOOOOOOOOOOOOOO

WP_223299999.1	GLOB	MLDQVDESAFSVVPPSLPKDESASLPDGMPDVAPSETPPPDAAARVVQSP	OOOOOOOOOOOOOOOOOOOOOOOOOOOOOOOOOOOOOOOOOOOOOOOOOO
		PDSTSPDSDGYSPAQARRPVSADTVGAPPAVLPEKDDLPSAAAAEPPAAS	OOOOOOOOOOOOOOOOOOOOOOOOOOOOOOOOOOOOOOOOOOOOOOOOOO
		SASAASKIKLFGTVEFKRPLSSLPAWLDLLKRNQADPIFLPEKHFNKKVT	OOOOOOOOOOOOOOOOOOOOOOOOOOOOOOOOOOOOOOOOOOOOOOOOOO
		WAQFKEKAKGKNAMDLLRYVNSFWNTWPYREDIDNWGQEDYWEIPAEFLK	OOOOOOOOOOOOOOOOOOOOOOOOOOOOOOOOOOOOOOOOOOOOOOOOOO
		KSGDCEDYSIIKYFTLKELGIPPETMRIVVVRDTIRNMAHAVLVVYMNND	OOOOOOOOOOOOOOOOOOOOOOOOOOOOOOOOOOOOOOOOOOOOOOOOOO
		AYVLDSLSNAVLSHTRFSHYSPQYSVNEFGRWAHLKGRKLK	OOOOOOOOOOOOOOOOOOOOOOOOOOOOOOOOOOOOOOOOO
WP_169570193.1	SP	MKVIRLLLLSSIICSFAFSGSNSNAQDADGLFGTLEIASSDLQALPQWRR	SSSSSSSSSSSSSSSSSSSSSSSSSOOOOOOOOOOOOOOOOOOOOOOOOO
		ALASFSDLKKAAKACDEDILKCNSQQMTLWRTKIQELEHSPKSIIMRQIN	OOOOOOOOOOOOOOOOOOOOOOOOOOOOOOOOOOOOOOOOOOOOOOOOOO
		QFVNKWQHNSDWATYRHQDYWASPLEFLTVGGDSEDFAIMKYVSLKELGF	OOOOOOOOOOOOOOOOOOOOOOOOOOOOOOOOOOOOOOOOOOOOOOOOOO
		SSEQMRIVVTRDVLRGVTHTILSVQLNNKSYVLDSQTDTVFQEKFIKYYV	OOOOOOOOOOOOOOOOOOOOOOOOOOOOOOOOOOOOOOOOOOOOOOOOOO
		PLYSLNETTRWAHIPSQYIAASNAARIDQ	OOOOOOOOOOOOOOOOOOOOOOOOOOOOO
WP_025899687.1	SP	MKLKTLVCSQKFLKASVLAVGLLAVPHIPSAEAVGIFGFKAYEHKDLKPF	SSSSSSSSSSSSSSSSSSSSSSSSSSSSSSSSSOOOOOOOOOOOOOOOOO
		PKWRKVLDTYKDQPGACGNGQLNACAYKKWQELVDELKDKPKKFQLAKVN	OOOOOOOOOOOOOOOOOOOOOOOOOOOOOOOOOOOOOOOOOOOOOOOOOO
		SYINLYRYILDPINWGVKDYWAIPKEFFAKFGDCEDYAIVKYFTLRALGW	OOOOOOOOOOOOOOOOOOOOOOOOOOOOOOOOOOOOOOOOOOOOOOOOOO
		KAEEMQILVLQDMNLRIAHAILTVEFENKKMVLDNQIGLVIDSKRIRHYR	OOOOOOOOOOOOOOOOOOOOOOOOOOOOOOOOOOOOOOOOOOOOOOOOOO
		PIYAVNENGWWRYKPR	OOOOOOOOOOOOOOOO
WP_161315810.1	SP	MKPTVFIKHTIAALLAGYIIVGPASSPSLRAEVTEKGLFGLIEISSDNLT	SSSSSSSSSSSSSSSSSSSSSSSSSSSSSSSOOOOOOOOOOOOOOOOOOO
		ALPLWLKVKASFPELADAAAKCDARIEDCQSQQMTLWRTKIAELQFSPER	OOOOOOOOOOOOOOOOOOOOOOOOOOOOOOOOOOOOOOOOOOOOOOOOOO
		IQMREINRFLNGWKYIPDETEAEASTENHWASPLEFLSNGGGSRDFAIMK	OOOOOOOOOOOOOOOOOOOOOOOOOOOOOOOOOOOOOOOOOOOOOOOOOO
		YVSLKELGFDPDAMRIVIANDVLRNKVQAVLSVTLRGQRYVLDSLNDTIL	OOOOOOOOOOOOOOOOOOOOOOOOOOOOOOOOOOOOOOOOOOOOOOOOOO
		EERFVNYYVPLYSVNENTRWAHIPQDFLIAQDDAGSKNDD	OOOOOOOOOOOOOOOOOOOOOOOOOOOOOOOOOOOOOOOO
WP_161338412.1	SP	MRIFAGHKRISLALFSAFFLFGTVAGAPARAEVTEEGLFGMVEISSDNLA	SSSSSSSSSSSSSSSSSSSSSSSSSSSSSSSOOOOOOOOOOOOOOOOOOO
		ALPRWLEVMSSFPELAKAASKCDDRIEDCQSQQMTLWRTKIAELQRTNRR	OOOOOOOOOOOOOOOOOOOOOOOOOOOOOOOOOOOOOOOOOOOOOOOOOO
		AQLQEINRFLNGWKHIQEEDAAATSDDWATPLQFLSNGGRSRDFAIMKYV	OOOOOOOOOOOOOOOOOOOOOOOOOOOOOOOOOOOOOOOOOOOOOOOOOO
		SLKELGFDPDSMRIVVANDVLRNKDHAVLSVSLRGKRYVLDSLNDTILEE	OOOOOOOOOOOOOOOOOOOOOOOOOOOOOOOOOOOOOOOOOOOOOOOOOO
		RLVNYYVPLYSVNEKRRWAHIPKDFLVAQEASGDEKND	OOOOOOOOOOOOOOOOOOOOOOOOOOOOOOOOOOOOOO
WP_229595160.1	SP	MIQTAIMAVCVVVCLGIGANVCVAAEKQKPVRLLGTMEFKGTKKLPKWSR	SSSSSSSSSSSSSSSSSSSSSSSSOOOOOOOOOOOOOOOOOOOOOOOOOO
		VVQKMKAWKGYFKDAATANHPSKKGWFSLKAQLKGKPEKEILKAVNKFFN	OOOOOOOOOOOOOOOOOOOOOOOOOOOOOOOOOOOOOOOOOOOOOOOOOO
		KWPYRLDKANWGVSEYWATPWEFLRKSGDCEDYSIAKFYALQEVGFTGDQ	OOOOOOOOOOOOOOOOOOOOOOOOOOOOOOOOOOOOOOOOOOOOOOOOOO
		LRIVAVKDAIRGIGHAILAVYATDDVYILDNQTLMILSHKKYRHYIPQYS	OOOOOOOOOOOOOOOOOOOOOOOOOOOOOOOOOOOOOOOOOOOOOOOOOO
		LNEKYRWMHVAPKKKSTYKKAKK	OOOOOOOOOOOOOOOOOOOOOOO
WP_338667776.1	SP	MRPVRGIYSAAAAALCAACIALALVRPSNALAADATTPEPAAPKSSAAKP	SSSSSSSSSSSSSSSSSSSSSSSSSSSSSSSSOOOOOOOOOOOOOOOOOO
		DTRRLFGTMEFKGKLQKLPKWTRVLEKMKAWKGYFRDGKTADHPSKKAWE	OOOOOOOOOOOOOOOOOOOOOOOOOOOOOOOOOOOOOOOOOOOOOOOOOO
		TLKAQVKGKSELERLRAVTKFFNKWPYRLDKANWGVSDYWETPWEFLKKS	OOOOOOOOOOOOOOOOOOOOOOOOOOOOOOOOOOOOOOOOOOOOOOOOOO
		GDCEDYAIAKFYALQELGFSPDQLRIVAVRDAIRGIGHAVLAVYAKDDIY	OOOOOOOOOOOOOOOOOOOOOOOOOOOOOOOOOOOOOOOOOOOOOOOOOO
		ILDNQTDMVLSHTKYKHYIPQYSVNEHYRWMHVAPQKRTTYEKAKQ	OOOOOOOOOOOOOOOOOOOOOOOOOOOOOOOOOOOOOOOOOOOOOO
WP_285907339.1	SP	MALAAEGGPSPSLLFGTVEFKGPISQLPKWTGVLEKMQGWKGFFEDPSMA	SSSSSSSSSSOOOOOOOOOOOOOOOOOOOOOOOOOOOOOOOOOOOOOOOO
		GVPARQSWQTLKNEIASAPPMDRVKAVNKYFNQWPYRLDQANYGQSDYWA	OOOOOOOOOOOOOOOOOOOOOOOOOOOOOOOOOOOOOOOOOOOOOOOOOO
		TPPEFMKKSGDCEDYSIAKFYALKELGFTGDDMRIVALRDTIRNIGHAVL	OOOOOOOOOOOOOOOOOOOOOOOOOOOOOOOOOOOOOOOOOOOOOOOOOO
		VVYLDGDAYVLDNQTVMVLSHAKYKHYMPQYSVNERFRWMHVPPTGKTTY	OOOOOOOOOOOOOOOOOOOOOOOOOOOOOOOOOOOOOOOOOOOOOOOOOO
		TKPKQ	OOOOO
WP_380255403.1	SP	MNTVPITTVHAQSLGDLANAAKGKGTALFNTVEFRSKSFKALPQWARVLK	SSSSSSSSSSSOOOOOOOOOOOOOOOOOOOOOOOOOOOOOOOOOOOOOOO
		KAKTEIRSFNACVSNAANCKTAQQNSWRKIINSAQNMSRRQKLDTVNKFF	OOOOOOOOOOOOOOOOOOOOOOOOOOOOOOOOOOOOOOOOOOOOOOOOOO
		NRWPYKLDSEVYGVSEYWASPNQFMRTSGDCEDYSIAKYYALQQLGFKDS	OOOOOOOOOOOOOOOOOOOOOOOOOOOOOOOOOOOOOOOOOOOOOOOOOO
		EMRIVILKDSIRGIGHAVLAIYEKNDILILDSLSNLILTHKKYRHYLPQY	OOOOOOOOOOOOOOOOOOOOOOOOOOOOOOOOOOOOOOOOOOOOOOOOOO
		SMNATTRWAHIK	OOOOOOOOOOOO

WP_148266956.1	SP	MVALCAACLAVVLVLSPRAVAAQATPDPATARPEAVRPATAVPSAVQPAP	SSSSSSSSSSSSSSSSSSSSSSOOOOOOOOOOOOOOOOOOOOOOOOOOOO
		ANHTTGKSDKRQLFGTLEFKGKIQKLPKWTRVLDKMKAWKGYFKDDRTND	OOOOOOOOOOOOOOOOOOOOOOOOOOOOOOOOOOOOOOOOOOOOOOOOOO
		HPSKKAWNTLKAQVGDKSEMDRLRAVTRFFNKWPYRLDMANWGVSDYWET	OOOOOOOOOOOOOOOOOOOOOOOOOOOOOOOOOOOOOOOOOOOOOOOOOO
		PWEFLKKSGDCEDYSIAKFYALQELGFTDDQLRIVAVKDAIRGIGHAVLA	OOOOOOOOOOOOOOOOOOOOOOOOOOOOOOOOOOOOOOOOOOOOOOOOOO
		VYATDDIYILDNQTDMVLSHTKYKHYIPQYSINEKYRWMHVAPQKRTTYE	OOOOOOOOOOOOOOOOOOOOOOOOOOOOOOOOOOOOOOOOOOOOOOOOOO
		KAKQ	OOOO
WP_074216486.1	GLOB	MLFKPPPVKKTRPKRQKKAAPSTGPLKDKNGKIRLFRTMELRGKNKTLAR	OOOOOOOOOOOOOOOOOOOOOOOOOOOOOOOOOOOOOOOOOOOOOOOOOO
		WQRIVDKMAAHKQLFLSNSLPTSGKNQRKRWKEFKSTINSAPRMQQLRKV	OOOOOOOOOOOOOOOOOOOOOOOOOOOOOOOOOOOOOOOOOOOOOOOOOO
		NAYFNKWPYRLDKENWGKREYWASPPEFVTRSGDCEDYAIAKYYALRELG	OOOOOOOOOOOOOOOOOOOOOOOOOOOOOOOOOOOOOOOOOOOOOOOOOO
		IPASSMRIVALMDNIRRLGHAVLVVYHDGDAWVLDNQTKLVLSHSKFSHY	OOOOOOOOOOOOOOOOOOOOOOOOOOOOOOOOOOOOOOOOOOOOOOOOOO
		VPQFSVNETNRWAHIPVKKNQPASKPKK	OOOOOOOOOOOOOOOOOOOOOOOOOOOO
WP_161624767.1	GLOB	MAAYKQEFLSNSLPTSGKNQRQRWKQFKSTIVSMPRMQQLRKINSYFNKW	OOOOOOOOOOOOOOOOOOOOOOOOOOOOOOOOOOOOOOOOOOOOOOOOOO
		PYRLDIENWGKQEYWATPPEFVKKSGDCEDYAIAKYYALRELGMPADSLR	OOOOOOOOOOOOOOOOOOOOOOOOOOOOOOOOOOOOOOOOOOOOOOOOOO
		IVAVMDNIRRLGHAVLVVFYDNDAWVLDNQTKLVLPHSKFSHYVPQFSVN	OOOOOOOOOOOOOOOOOOOOOOOOOOOOOOOOOOOOOOOOOOOOOOOOOO
		ETNRWAHIPVKKNQLASKPKK	OOOOOOOOOOOOOOOOOOOOO
WP_231895534.1	SP	MRHWTHSTVFRVTLICFACSLVIAAQPIQASSEYTKNTSRPSVSTKGASL	SSSSSSSSSSSSSSSSSSSSSSSSSSSSSSOOOOOOOOOOOOOOOOOOOO
		SKGKKKSAFRLLFKPPPIKKSAPKPRKRVAASKGPLKDTKGNIRLFRTIE	OOOOOOOOOOOOOOOOOOOOOOOOOOOOOOOOOOOOOOOOOOOOOOOOOO
		LKGKNKTLARWKRIVDKMAVHKQLYLSNSLPTSGKNQQKRWKQFKSTVVS	OOOOOOOOOOOOOOOOOOOOOOOOOOOOOOOOOOOOOOOOOOOOOOOOOO
		KSRMQQLRKVNTYFNNWPYRLDKENWGKQEYWASPPEFVKKSGDCEDYAI	OOOOOOOOOOOOOOOOOOOOOOOOOOOOOOOOOOOOOOOOOOOOOOOOOO
		AKYYALRELGLSASSMRIVALMDNIRRLGHAVLVVYLNGDAWVLDNQTKL	OOOOOOOOOOOOOOOOOOOOOOOOOOOOOOOOOOOOOOOOOOOOOOOOOO
		VLSHSKFSHYVPQFSVNETNRWAHIPVKKNQPASKPKK	OOOOOOOOOOOOOOOOOOOOOOOOOOOOOOOOOOOOOO
WP_072697218.1	SP	MGLIHKNNQKAVYKIWLIFFALLFLQQGLISILSANSPPRLFGTVEFKTV	SSSSSSSSSSSSSSSSSSSSSSSSSSSSSSSSSSSOOOOOOOOOOOOOOO
		LKDMPKWQRVLSAEKKNPTFTDQFHTLLPKNLAEKWQTIEKNFPSFSTQE	OOOOOOOOOOOOOOOOOOOOOOOOOOOOOOOOOOOOOOOOOOOOOOOOOO
		KLKAVNMFFNQWPYRTDSSLFGLEDYWATPKEFLKKSGDCEDYAIIKFFA	OOOOOOOOOOOOOOOOOOOOOOOOOOOOOOOOOOOOOOOOOOOOOOOOOO
		LKYLGIPEEQMRIVVVKDTIRNLGHGVLAVYIDDNIYILDNLSNTPLSHT	OOOOOOOOOOOOOOOOOOOOOOOOOOOOOOOOOOOOOOOOOOOOOOOOOO
		TIRNYQPAFSLNSQFRWAHLPVKKKK	OOOOOOOOOOOOOOOOOOOOOOOOOO
WP_169560244.1	SP	MMPLSATLLPFALAASLLMAGSAQANTMADIPPPDGLFGTVEIPSRDLQA	SSSSSSSSSSSSSSSSSSSSSSSSSOOOOOOOOOOOOOOOOOOOOOOOOO
		LPQWTRVIGDFKKSDRAARLCDQDIQKCASQQMTLWRAKIQELTEAERST	OOOOOOOOOOOOOOOOOOOOOOOOOOOOOOOOOOOOOOOOOOOOOOOOOO
		QLREINRFINKWRHVSDMENYGHADYWASPLEFITNGGDSEDFAIMKYIS	OOOOOOOOOOOOOOOOOOOOOOOOOOOOOOOOOOOOOOOOOOOOOOOOOO
		LKELGVPVANMRIVVTSDVLRGQNHTILYVRENGRRLVLDSQNDTVQQEK	OOOOOOOOOOOOOOOOOOOOOOOOOOOOOOOOOOOOOOOOOOOOOOOOOO
		NVKYYVPFYSVNETTRWAHVANEATEIAGATEKPRDD	OOOOOOOOOOOOOOOOOOOOOOOOOOOOOOOOOOOOO
WP_242012421.1	SP	MASGRLIRWAGMGALAALVLAVWLAVGGVAREGGTDLGPEPEAQAAPSTE	SSSSSSSSSSSSSSSSSSSSSSSSSSSSSSSSOOOOOOOOOOOOOOOOOO
		AGAMPRDEPPPASEENPPQKEDEPALDGANQAGEPAPVTEQSSSVEENAT	OOOOOOOOOOOOOOOOOOOOOOOOOOOOOOOOOOOOOOOOOOOOOOOOOO
		APASTSVPEPEPAVQPVDVPASTPKPGPAPGPEPSTKQQPAPAPAEVRER	OOOOOOOOOOOOOOOOOOOOOOOOOOOOOOOOOOOOOOOOOOOOOOOOOO
		KPESALKSEPAPKVVPKPAPAPVPEPAKATVPAVPASLPDAKEVKPAPKP	OOOOOOOOOOOOOOOOOOOOOOOOOOOOOOOOOOOOOOOOOOOOOOOOOO
		APKAVAKPAPKPGIKLFGTVEFKGRLTKLPLWTAVLKKMRGWKGYFRDPS	OOOOOOOOOOOOOOOOOOOOOOOOOOOOOOOOOOOOOOOOOOOOOOOOOO
		MAKLPSRSGWNKLVRETSSLSLMGRLKAVNKFFNRWPYRLDAANYGKSDY	OOOOOOOOOOOOOOOOOOOOOOOOOOOOOOOOOOOOOOOOOOOOOOOOOO
		WATPKEFLMKSGDCEDYAIAKFYALQELGFRNVPMRIVAVRDVIRNIGHA	OOOOOOOOOOOOOOOOOOOOOOOOOOOOOOOOOOOOOOOOOOOOOOOOOO
		VLAVYLDDQVYILDNQTDMVLPHTRYKHYVPQYSVDEHYRWMHIPVSGKA	OOOOOOOOOOOOOOOOOOOOOOOOOOOOOOOOOOOOOOOOOOOOOOOOOO
		KQRQ	OOOO
WP_251935996.1	SP	MTGILLKFRVSILALVLGSALLVSPLATNAEAVGLFGYRATPYDGLKPFP	SSSSSSSSSSSSSSSSSSSSSSSSSSSSSSSSOOOOOOOOOOOOOOOOOO
		KWRQVLKTYKEEPGDCQSGKYNRCAHQKWLALIEDWRELPKQEQLNRVNQ	OOOOOOOOOOOOOOOOOOOOOOOOOOOOOOOOOOOOOOOOOOOOOOOOOO
		HMNLFRYILDPVNWGVRDYWEIPKEFFARFGDCEDYAIVKYFTLRALGWS	OOOOOOOOOOOOOOOOOOOOOOOOOOOOOOOOOOOOOOOOOOOOOOOOOO
		AEDMKIVVLQDMNLGIAHAILVVEFDGKNLVLDNQIGQVVDAKRIKHYRP	OOOOOOOOOOOOOOOOOOOOOOOOOOOOOOOOOOOOOOOOOOOOOOOOOO
		IYSVNEIGWWRHKEP	OOOOOOOOOOOOOOO
WP_085882215.1	SP	MSGYRRQRHGLAALMALLLACGLAGTVTRAAEAPLAVETGLFGSLEFPSH	SSSSSSSSSSSSSSSSSSSSSSSSSSSSSSOOOOOOOOOOOOOOOOOOOO
		DLGAFPQWRHILAGLPAFRMAVNACARDETNCSNFSVARWVARKMLLEGQ	OOOOOOOOOOOOOOOOOOOOOOOOOOOOOOOOOOOOOOOOOOOOOOOOOO
		PNSRQLAGVNQFVNSLATAPDAPLAKPDGRWAGPKETLREGGDPLDLAVL	OOOOOOOOOOOOOOOOOOOOOOOOOOOOOOOOOOOOOOOOOOOOOOOOOO
		KYVLLRELGFADHQLRIVIVTDTLRDRPHAVLIVRTRGTWVLDSRSDHLQ	OOOOOOOOOOOOOOOOOOOOOOOOOOOOOOOOOOOOOOOOOOOOOOOOOO
		KAADMRFYLPHISLNGETRWAHMAPWLALRDGAGTAAEPEHRGGMPQ	OOOOOOOOOOOOOOOOOOOOOOOOOOOOOOOOOOOOOOOOOOOOOOO

WP_161338416.1	SP	MTSAGLRKFVVLRVVILAALLLLTPSTSPAQAAGLFGYQETPYSGLKPFP	SSSSSSSSSSSSSSSSSSSSSSSSSSSSSSSSOOOOOOOOOOOOOOOOOO
		KWRKVLNTYKDDPGDCVTGKYNRCAYQKWVALIDEWQQLSKEEQLAQVNR	OOOOOOOOOOOOOOOOOOOOOOOOOOOOOOOOOOOOOOOOOOOOOOOOOO
		HMNLFRYILDPVNWGVKDYWEIPKEFFARFGDCEDYAIVKYFTLRALGWP	OOOOOOOOOOOOOOOOOOOOOOOOOOOOOOOOOOOOOOOOOOOOOOOOOO
		AEDMKIVVLQDVNLGVAHAILVVNFKGKKLVLDNQIGQVVDSSRIRHYKP	OOOOOOOOOOOOOOOOOOOOOOOOOOOOOOOOOOOOOOOOOOOOOOOOOO
		IYSVNENGWWRHKPKIANN	OOOOOOOOOOOOOOOOOOO
WP_169543097.1	SP	MIRKSFISTLAVAGLISIATGNARAITDTDLGLFGTIEIASDDLTSLPQW	SSSSSSSSSSSSSSSSSSSSSSSSSOOOOOOOOOOOOOOOOOOOOOOOOO
		KRVISSFKELDKQAALCDRDIQKCKSQQMTLWRAKIQELELETANAKVRQ	OOOOOOOOOOOOOOOOOOOOOOOOOOOOOOOOOOOOOOOOOOOOOOOOOO
		VNSFINKWRMTTDEETYKRSDHWAEPLEFIVNGGDSEDFAIMKYISLKEL	OOOOOOOOOOOOOOOOOOOOOOOOOOOOOOOOOOOOOOOOOOOOOOOOOO
		GIPASSMRIVVTNDVLRGKTHTILSVDTGSERYVLDSQNNTILREDLVRY	OOOOOOOOOOOOOOOOOOOOOOOOOOOOOOOOOOOOOOOOOOOOOOOOOO
		YVPFYSLNETKRWAHVPKQAADLKKAAGVEKND	OOOOOOOOOOOOOOOOOOOOOOOOOOOOOOOOO
WP_051307240.1	SP	MTRRPRALLTLIAAAILLPGLFLAERAEPMGAQGPGPEIRAGQEQAQGAE	SSSSSSSSSSSSSSSSSSSSSSSSOOOOOOOOOOOOOOOOOOOOOOOOOO
		AQQGAKPEPDGEAEKNPSPRNNASVAGDQGREDGAATPAELNGQSVRDRA	OOOOOOOOOOOOOOOOOOOOOOOOOOOOOOOOOOOOOOOOOOOOOOOOOO
		EVTGASDAPDRMEDQGVPEPVQAVSDEKAEAADPAPEQVADAERVRPRPP	OOOOOOOOOOOOOOOOOOOOOOOOOOOOOOOOOOOOOOOOOOOOOOOOOO
		ADPSENVPAQPLTGQAVARPWRGESRTQEAASRASGQPQPSGQPGQDAKG	OOOOOOOOOOOOOOOOOOOOOOOOOOOOOOOOOOOOOOOOOOOOOOOOOO
		QPVRLFQTAAFRGNFDALPKWKRVLSKADAQVRVLNSCSGAGCPPGATSW	OOOOOOOOOOOOOOOOOOOOOOOOOOOOOOOOOOOOOOOOOOOOOOOOOO
		QRIMSQARGLAPMEQLKAVNTFFNKWPYRLDQDAYGTSDWWATPQEFLKI	OOOOOOOOOOOOOOOOOOOOOOOOOOOOOOOOOOOOOOOOOOOOOOOOOO
		SGDCEDFAITKYFALRELGYSPDDLRIVILKDRIRGIAHAILAVFIDGDA	OOOOOOOOOOOOOOOOOOOOOOOOOOOOOOOOOOOOOOOOOOOOOOOOOO
		LVLDNVTSVIFSHDKYKHYAPYYSLNERYRWSHIPLDTKP	OOOOOOOOOOOOOOOOOOOOOOOOOOOOOOOOOOOOOOOO
WP_071544367.1	SP	MRAVRRGIYSALAAALCAACIAAAVIRPRAALAAEPAAAEVSAPKPDKRQ	SSSSSSSSSSSSSSSSSSSSSSSSSSSSSSSSSOOOOOOOOOOOOOOOOO
		LFGTMEFKGKLQKLPKWTGVLAKMKAWKGYFKDDRTANHPSKKAWETLKT	OOOOOOOOOOOOOOOOOOOOOOOOOOOOOOOOOOOOOOOOOOOOOOOOOO
		QARGKSELDRLRAVTKFFNKWPYRLDKANWGVSDYWETPWEFLKKSGDCE	OOOOOOOOOOOOOOOOOOOOOOOOOOOOOOOOOOOOOOOOOOOOOOOOOO
		DYSIAKFYALQELGFTGDQMRIVAVRDAIRGIGHAVLAVYAKDDIYILDN	OOOOOOOOOOOOOOOOOOOOOOOOOOOOOOOOOOOOOOOOOOOOOOOOOO
		QTDMVLPHTKYKHYIPQYSVNEHYRWMHVAPQKRTTYEKAKQ	OOOOOOOOOOOOOOOOOOOOOOOOOOOOOOOOOOOOOOOOOO
WP_015416032.1	SP	MTRASLIRRMAAVALLVAAFAIYPMVGGTTQTQGAGNTEKEAAADDTVAE	SSSSSSSSSSSSSSSSSSSSSSSSSSSOOOOOOOOOOOOOOOOOOOOOOO
		NPSVESGSEETQVREVISSGRQETPPKKAVSTQAVTKEVGIHADRGASTP	OOOOOOOOOOOOOOOOOOOOOOOOOOOOOOOOOOOOOOOOOOOOOOOOOO
		VVPASSQAEAVFSSTPVTSKKTSPVLKSSQPTSPVARKEKVPVVRSVPAP	OOOOOOOOOOOOOOOOOOOOOOOOOOOOOOOOOOOOOOOOOOOOOOOOOO
		RKAGKETPQSKKILLFDTIEFKGKIRKLPKWSRVLAKMKAWKGYFKDAQS	OOOOOOOOOOOOOOOOOOOOOOOOOOOOOOOOOOOOOOOOOOOOOOOOOO
		AKLPSRSGWVKLRRDVAGLDRMGRLKAVNKFFNKWPYRLDAANYGASDYW	OOOOOOOOOOOOOOOOOOOOOOOOOOOOOOOOOOOOOOOOOOOOOOOOOO
		ATPMEFLRKSGDCEDYSIAKFYALQELGFTGDSMRIVALKDRIRNIGHAV	OOOOOOOOOOOOOOOOOOOOOOOOOOOOOOOOOOOOOOOOOOOOOOOOOO
		LVVYLGNDAYVLDNQTNMVLSHVKYKHYVPQYSVNEHFRWMHVPRSKKSV	OOOOOOOOOOOOOOOOOOOOOOOOOOOOOOOOOOOOOOOOOOOOOOOOOO
		RRSVKKK	OOOOOOO
WP_161315807.1	SP	MTGARSQFFAILTICILVTCVLASPRISEAKSGGLFGYQATPYSGLKPFP	SSSSSSSSSSSSSSSSSSSSSSSSSSSSSSOOOOOOOOOOOOOOOOOOOO
		KWRKVLATYKDEPGDCVSGKYNRCAHQKWLALIEEWRELPKEEQLKKINR	OOOOOOOOOOOOOOOOOOOOOOOOOOOOOOOOOOOOOOOOOOOOOOOOOO
		HMNLFRYILDPVNWGVRDYWEIPKEFFARFGDCEDYAIVKYFTLRALGWP	OOOOOOOOOOOOOOOOOOOOOOOOOOOOOOOOOOOOOOOOOOOOOOOOOO
		DKDMKIVVLQDVNLGIAHAILVVKYKGKNLVLDNQIGQVVDATRIRHYRP	OOOOOOOOOOOOOOOOOOOOOOOOOOOOOOOOOOOOOOOOOOOOOOOOOO
		IYSVNEAGWWRHKP	OOOOOOOOOOOOOO
WP_167230619.1	SP	MDRRAFLRSSALMLGTAAGGLVLDAWAKPAAAQSLGDFMKSTRNSGNALF	SSSSSSSSSSSSSSSSSSSSSSSSSSSSSSSSOOOOOOOOOOOOOOOOOO
		GSTEISSATLKALPQWARVLSKMKKERSSFQACLNNPAACTTAGLKSWRE	OOOOOOOOOOOOOOOOOOOOOOOOOOOOOOOOOOOOOOOOOOOOOOOOOO
		VALAAKGKPRAAMLKTVNQYFNRWPYKIDQELYGVSEFWATPLEFMKRSG	OOOOOOOOOOOOOOOOOOOOOOOOOOOOOOOOOOOOOOOOOOOOOOOOOO
		DCEDYSIAKFFALRDLGVSNDEMRVVILMDRIRRIGHAVLAVYQPNDILI	OOOOOOOOOOOOOOOOOOOOOOOOOOOOOOOOOOOOOOOOOOOOOOOOOO
		LDSLTDLIFPHKKYRHYVPQYSMNETKRWAHFYDKKRTASL	OOOOOOOOOOOOOOOOOOOOOOOOOOOOOOOOOOOOOOOOO
WP_081649842.1	SP	MPDRGKGIASERALGGLCLAFLVSAGLSALWLIVDEERATRSQITYHNEG	SSSSSSSSSSSSSSSSSSSSSSSSSSSSSSSSOOOOOOOOOOOOOOOOOO
		SQQQQTSTGHSPGEVLSFPWPEILLAQSSGSSDLSRLAKGLFGTIEFRSS	OOOOOOOOOOOOOOOOOOOOOOOOOOOOOOOOOOOOOOOOOOOOOOOOOO
		NLEAFPQWLRVMEELEDLEELKRLCDEENECLNNRFAAWRANVKSLENRH	OOOOOOOOOOOOOOOOOOOOOOOOOOOOOOOOOOOOOOOOOOOOOOOOOO
		PRAQMDELNRFINEAMPYIEDPQVYGRSDYWAAPQEFLRHSGDCEDYAIL	OOOOOOOOOOOOOOOOOOOOOOOOOOOOOOOOOOOOOOOOOOOOOOOOOO
		KYASLRELGFPEDSLRIVVLKDTLRDLPHAVLAVDYDDKTYILDNNFSVV	OOOOOOOOOOOOOOOOOOOOOOOOOOOOOOOOOOOOOOOOOOOOOOOOOO
		LNDDQLPQYAPQYSLNTNHRWVHISPSSVE	OOOOOOOOOOOOOOOOOOOOOOOOOOOOOO

WP_142895221.1	SP	MAALASVPSPGRAATLLDLTKTIDTAAPGIFNTIEFRSKSFKALPQWRRV<br>LDQFKRERKAFLACTNDAANCSSEVQKSWREIILEARDLDRAAKLNAVNR<br>YFNRWPYKLDKEVFGVNEYWASPVEFMTRSGDCEDFSIAKYFALKELGFT<br>QEEMRIVILMDRIRNIGHAVLAIYEKNDILVLDSLSNVILSHSKYKHYLP<br>QYSMNETTRWAHFVQQ	SSSSSSSSSSSSSOOOOOOOOOOOOOOOOOOOOOOOOOOOOOOOOOOOOO<br>OOOOOOOOOOOOOOOOOOOOOOOOOOOOOOOOOOOOOOOOOOOOOOOOOO<br>OOOOOOOOOOOOOOOOOOOOOOOOOOOOOOOOOOOOOOOOOOOOOOOOOO<br>OOOOOOOOOOOOOOOOOOOOOOOOOOOOOOOOOOOOOOOOOOOOOOOOOO<br>OOOOOOOOOOOOOOOO
WP_243544993.1	SP	MKQKPRAWRTRAATLAAVCSAVLAACLWVGLRPESPSAAHVSPGVADSGT<br>HVVRTVPRARPDVPDMVVQNSQPAPLHTPAPAPVHTPAPAPIHAPAPAPA<br>PVHTPPPAPLQTPAPAPMHTPPPAPVSIPAPSVKPAVSGTAVASGANGKG<br>GRPHLFGTVEFKGKIKALPKWTGVLDRMKAWKGYFRDASTNGVSGKAGWQ<br>ELRNGAAGKDRLEQLKAVNVFFNKWPYRLDQANYGMRDYWASPPEFLHKS<br>GDCEDYAIAKYYALKELGFSMDDLRIVAVKDTIRNIGHAVLAVYVDDQIY<br>ILDNQTVMVLPHDKYTHYNPQYSVNEKYRWFHVPPTKKTTYGTPAVK	SSSSSSSSSSSSSSSSSSSSSSSSSSSSSSSSSSSSSSOOOOOOOOOOOO<br>OOOOOOOOOOOOOOOOOOOOOOOOOOOOOOOOOOOOOOOOOOOOOOOOOO<br>OOOOOOOOOOOOOOOOOOOOOOOOOOOOOOOOOOOOOOOOOOOOOOOOOO<br>OOOOOOOOOOOOOOOOOOOOOOOOOOOOOOOOOOOOOOOOOOOOOOOOOO<br>OOOOOOOOOOOOOOOOOOOOOOOOOOOOOOOOOOOOOOOOOOOOOOOOOO<br>OOOOOOOOOOOOOOOOOOOOOOOOOOOOOOOOOOOOOOOOOOOOOOOOOO<br>OOOOOOOOOOOOOOOOOOOOOOOOOOOOOOOOOOOOOOOOOOOOOOO
WP_169560248.1	SP	MKKRNHPKSRLLLFSSALAFGLSVLVAPPHASANGIFGFREIAYTDLKPF<br>PKWQKVLDTYKERPGNCDSGQLNACAYKKWEELIEELQDAPKAKQLERVN<br>TYINLFRYILDPINWGVKDYWEIPREFFARFGDCEDYAIVKYFTLRALGW<br>KAEEMKIVVLQDMNLRIAHAILAVELDGKQMILDNQIGLVVDAKRIKHYR<br>PIYSVNELGWWRHKPAVN	SSSSSSSSSSSSSSSSSSSSSSSSSSSSSSSSSOOOOOOOOOOOOOOOOO<br>OOOOOOOOOOOOOOOOOOOOOOOOOOOOOOOOOOOOOOOOOOOOOOOOOO<br>OOOOOOOOOOOOOOOOOOOOOOOOOOOOOOOOOOOOOOOOOOOOOOOOOO<br>OOOOOOOOOOOOOOOOOOOOOOOOOOOOOOOOOOOOOOOOOOOOOOOOOO<br>OOOOOOOOOOOOOOOOOO
WP_265826327.1	SP	MIKRVRLRQTSAHLLPLVRLVCLCLTVAIIPAIILAPTGRLWHAHAQEGS<br>SMAAKMLAGSKAAPAQQKPSAFRQMLSGTPAAKPAPASPDGKVRLFNTME<br>FKGPLKSLQQWNRIIQAMKAGKNKLSAVLPASGKNNAKASWEAFRNSIKD<br>EPLMEKLKKLNAHFNKYPYRLDQEVWGQSDYWATPAEFALKSGDCEDYSI<br>TKYYALRELGVPAEDLRIVALKDRIRGIGHAVLVVYAEGTAWVLDNQTDL<br>VLSHDRYSHYLPQYSVNEFNRWAHVPVK	SSSSSSSSSSSSSSSSSSSSSSSSSSSSSSSSSSSSSSSSSSSSSSOOOO<br>OOOOOOOOOOOOOOOOOOOOOOOOOOOOOOOOOOOOOOOOOOOOOOOOOO<br>OOOOOOOOOOOOOOOOOOOOOOOOOOOOOOOOOOOOOOOOOOOOOOOOOO<br>OOOOOOOOOOOOOOOOOOOOOOOOOOOOOOOOOOOOOOOOOOOOOOOOOO<br>OOOOOOOOOOOOOOOOOOOOOOOOOOOOOOOOOOOOOOOOOOOOOOOOOO<br>OOOOOOOOOOOOOOOOOOOOOOOOOOOO
WP_245622974.1	GLOB	MKLLNACANNAANCKTPQQKSWRKIIHSAKNMSRRQKLDTVNKFFNRWPY<br>KLDHEVYGVSEYWASPNQFMRTSGDCEDYSIAKFYALKQLGFSDNEMRIV<br>ILKDSIRGIGHAVLAIYEKNDILILDSLSNLILTHTKYRHYIPQYSMNST<br>TRWAHIK	OOOOOOOOOOOOOOOOOOOOOOOOOOOOOOOOOOOOOOOOOOOOOOOOOO<br>OOOOOOOOOOOOOOOOOOOOOOOOOOOOOOOOOOOOOOOOOOOOOOOOOO<br>OOOOOOOOOOOOOOOOOOOOOOOOOOOOOOOOOOOOOOOOOOOOOOOOOO<br>OOOOOOO
WP_371384792.1	SP	MVALCAACIAVALVRPPDAFAAAPGADAAAARPDKRQLFGTMEFKGKLQK<br>LPKWTRVLEKMKAWKGYFKDDRTANHPSNKAWSTLKAQFKDKPELDRLRA<br>VTKFFNKWPYRLDMANWGVSDYWETPWEFLKKSGDCEDYAIAKFYALQEL<br>GFSPDQMRIVAVRDAIRGIGHAVLAVYAKDDIYILDNQTDMVLSHTKYKH<br>YIPQYSVNEHYRWMHVAPQKRTTYEKAKQ	SSSSSSSSSSSSSSSSSSSSSOOOOOOOOOOOOOOOOOOOOOOOOOOOOO<br>OOOOOOOOOOOOOOOOOOOOOOOOOOOOOOOOOOOOOOOOOOOOOOOOOO<br>OOOOOOOOOOOOOOOOOOOOOOOOOOOOOOOOOOOOOOOOOOOOOOOOOO<br>OOOOOOOOOOOOOOOOOOOOOOOOOOOOOOOOOOOOOOOOOOOOOOOOOO<br>OOOOOOOOOOOOOOOOOOOOOOOOOOOOO
WP_279521168.1	SP	MRPVRGIYLALAVALCAVCLAAILVRPPDAQAADPGKRQLFGTLEFKGKL<br>QKLPKWTRVLDKMKAWKGYFNDSQTSGHPSRKAWETLKAQVGDKSEMDRL<br>RAVTKFFNKWPYRLDKTNWGVSEYWATPWVFLKKSGDCEDYSIAKFYALE<br>ELGFTGDQLRIVAVRDAIRGIGHAVLAVYASDDIYILDNQTNMVLSHSRY<br>RHYVPQYSVNEKYRWMHVAPKKRTAYEKAKN	SSSSSSSSSSSSSSSSSSSSSSSSSSSSSSSSOOOOOOOOOOOOOOOOOO<br>OOOOOOOOOOOOOOOOOOOOOOOOOOOOOOOOOOOOOOOOOOOOOOOOOO<br>OOOOOOOOOOOOOOOOOOOOOOOOOOOOOOOOOOOOOOOOOOOOOOOOOO<br>OOOOOOOOOOOOOOOOOOOOOOOOOOOOOOOOOOOOOOOOOOOOOOOOOO<br>OOOOOOOOOOOOOOOOOOOOOOOOOOOOOOO
WP_162175094.1	SP	MVDRHDQSADLYVPWPEILLAQSSGDSDLSSLAKGLFGTIEFRSSNLQAF<br>PQWLRVLDELEELKELNRLCDEENECLNNRFSAWRARVKSLENRHPMSQM<br>DELNRFINEAMPYLEDPQVYGQSDYWAAPQEFLRHSGDCEDYAILKYASL<br>RELGFPEDSMRIVVLQDTLRDVPHAVLAVDYNDKTFILDNNFSVVLNDDQ<br>LPQYAPQYSLNANHRWVHISPSSVE	SSSSSSSSSSOOOOOOOOOOOOOOOOOOOOOOOOOOOOOOOOOOOOOOOO<br>OOOOOOOOOOOOOOOOOOOOOOOOOOOOOOOOOOOOOOOOOOOOOOOOOO<br>OOOOOOOOOOOOOOOOOOOOOOOOOOOOOOOOOOOOOOOOOOOOOOOOOO<br>OOOOOOOOOOOOOOOOOOOOOOOOOOOOOOOOOOOOOOOOOOOOOOOOOO<br>OOOOOOOOOOOOOOOOOOOOOOOOO
WP_207045699.1	SP	MKQLLIFRRTTALLLAGLFLLGPATAISARAEVTEKGLFGLVEISSDNLA<br>ALPHWLKVKASFPLLANAASKCDDRIEDCQSQQMTLWRTKIAELQRSPKR<br>IQMQEINRFLNGWKYIPDDVEASPGSPWASPLEFLSNGGGSKDFAVMKYI<br>SLKELGFDPDSMRIVIANDVLRNKVHAVLSVSQQGKRYVLDSLNDTILEE<br>RFVNYYVPLYSVNENTRWAHIPIDFLTAQDAAGSNTND	SSSSSSSSSSSSSSSSSSSSSSSSSSSSSSSOOOOOOOOOOOOOOOOOOO<br>OOOOOOOOOOOOOOOOOOOOOOOOOOOOOOOOOOOOOOOOOOOOOOOOOO<br>OOOOOOOOOOOOOOOOOOOOOOOOOOOOOOOOOOOOOOOOOOOOOOOOOO<br>OOOOOOOOOOOOOOOOOOOOOOOOOOOOOOOOOOOOOOOOOOOOOOOOOO<br>OOOOOOOOOOOOOOOOOOOOOOOOOOOOOOOOOOOOOO

WP_020591926.1	SP	MNFRQLTKATTLAAVLLSAMPAPVVQAQTLGDLAKAAKGKGTALFNTVEF<br>QSKSFKALPQWARVLEKAKTDMNLFQACSNDINKCKTAQQNSWRKIIHAA<br>RNMTRRQKLDAVNRFFNKWPYKLDQEVYGVSEYWASPNQFMKTSGDCEDY<br>SIAKYYALKELGFQDSELRIVILLDRIRGIGHAVLAIYEPNDILILDSLS<br>NLILTHTKYRHYVPQYSMNATTRWAHIK	SSSSSSSSSSSSSSSSSSSSSSSSSSSOOOOOOOOOOOOOOOOOOOOOOO<br>OOOOOOOOOOOOOOOOOOOOOOOOOOOOOOOOOOOOOOOOOOOOOOOOOO<br>OOOOOOOOOOOOOOOOOOOOOOOOOOOOOOOOOOOOOOOOOOOOOOOOOO<br>OOOOOOOOOOOOOOOOOOOOOOOOOOOOOOOOOOOOOOOOOOOOOOOOOO<br>OOOOOOOOOOOOOOOOOOOOOOOOOOOO
WP_169570190.1	SP	MKTLLRTSLVLGCLISSPTAAFSESPDIFGFKAYTHTDLKPFPKWRKVLN<br>TYKNEPGPCGNGQLNACAYDKWKELIDQLKDEPKKYQLTKVNSYLNLYRY<br>VLDPINWGIKDYWEIPKEFFAKFGDCEDYAIVKYFTLRALGWDADDMKIV<br>VLQDMNLRIAHAILTVKFEEKKMVLDNQIGLVIDAKRIRHYRPIYALNEN<br>GWWRYKPS	SSSSSSSSSSSSSSSSSSSSSSSOOOOOOOOOOOOOOOOOOOOOOOOOOO<br>OOOOOOOOOOOOOOOOOOOOOOOOOOOOOOOOOOOOOOOOOOOOOOOOOO<br>OOOOOOOOOOOOOOOOOOOOOOOOOOOOOOOOOOOOOOOOOOOOOOOOOO<br>OOOOOOOOOOOOOOOOOOOOOOOOOOOOOOOOOOOOOOOOOOOOOOOOOO<br>OOOOOOOO
WP_193370322.1	SP	MSLPGLLGLFCLGVLLAAPRPVAGQDLASMIQATKNAQDENTLFGSIEIR<br>SDSLKGLQQWQRVVSAMRTAGPAFNNCAKSAGACTTSMLKRWREIVVAAQ<br>RLPHNERLKSVNTYFNGWPYKLDMEVYGVREYWATPKEFIIRSGDCEDYS<br>IAKYYALRNVGFSKDELRIVALRDTIRGFGHAVMAAYVNGDILILDNTSQ<br>LVLSHKKYGHYEPQVSMNETSRWAHVPGKKRELKAPSGFALSASTLFQKR	SSSSSSSSSSSSSSSSSSSSSSSSOOOOOOOOOOOOOOOOOOOOOOOOOO<br>OOOOOOOOOOOOOOOOOOOOOOOOOOOOOOOOOOOOOOOOOOOOOOOOOO<br>OOOOOOOOOOOOOOOOOOOOOOOOOOOOOOOOOOOOOOOOOOOOOOOOOO<br>OOOOOOOOOOOOOOOOOOOOOOOOOOOOOOOOOOOOOOOOOOOOOOOOOO<br>OOOOOOOOOOOOOOOOOOOOOOOOOOOOOOOOOOOOOOOOOOOOOOOOOO
WP_245170854.1	SP	MPKLRYSKFALVAALSGGMAALLVCLLFWVNGWNPAQAGSLNLQPRAAIG<br>NAEVSEPPAVSSAAAENNAAPSVAATDQASTGAEQNGSNGANSALPTADQ<br>QPGLQSGQQSGPQTIQQADADSSVQPQTDADSAEQPPSPESSQQTGKPDS<br>SAAVNAPAKVATPDAAKGAAQSAAQPAAPAHDSKVQLFGTVEFKRPLSTL<br>PGWLDLLKRNQMDPIFIPGKVFKKGVTWDTFKSKAPVNNKMELLRYVNSF<br>WNTWPYVEDIVNWRQEDYWEIPAEFLKKSGDCEDYSIIKYFTLKELGIPP<br>ESMRVVVVRDTIRNFAHAVLVVYLNDDAFILDNLSNSVLSHTKVRQYSPQ<br>YSVNEFGRWAHLKGRKID	SSSSSSSSSSSSSSSSSSSSSSSSSSSSSSSSSSSSSSOOOOOOOOOOOO<br>OOOOOOOOOOOOOOOOOOOOOOOOOOOOOOOOOOOOOOOOOOOOOOOOOO<br>OOOOOOOOOOOOOOOOOOOOOOOOOOOOOOOOOOOOOOOOOOOOOOOOOO<br>OOOOOOOOOOOOOOOOOOOOOOOOOOOOOOOOOOOOOOOOOOOOOOOOOO<br>OOOOOOOOOOOOOOOOOOOOOOOOOOOOOOOOOOOOOOOOOOOOOOOOOO<br>OOOOOOOOOOOOOOOOOOOOOOOOOOOOOOOOOOOOOOOOOOOOOOOOOO<br>OOOOOOOOOOOOOOOOOOOOOOOOOOOOOOOOOOOOOOOOOOOOOOOOOO<br>OOOOOOOOOOOOOOOOOO
WP_407844443.1	SP	MAVIFFLSVLAWRHFSPAIARAEQGHNAEIAAGRPIDDDVAPERPLSASL<br>NSVDDVPPASLAEVAGQSQPFFSEPGGDEPDAAALSEKDAASVQENDGDA<br>REQAQAAEHAKTSADVGSSSPEPSAAPGSKVQLFGTVEFKRPLSSLPGWL<br>DVLKRNKKDPIFIPGKYFKKSVTWDSFKKNAQGKNTMELLRYVNSFWNTW<br>PYKEDIANWGVEDYWAIPAEFLKKSGDCEDYAIVKYFTLKELGIPAENMR<br>IVVVRDTLRNLAHAVLAVYLNGDAYILDNLSNTVLSHSRIRQYSPQYSVN<br>ESGRWAHLKGRKLK	SSSSSSSSSSSSSSSSSSSSSSOOOOOOOOOOOOOOOOOOOOOOOOOOOO<br>OOOOOOOOOOOOOOOOOOOOOOOOOOOOOOOOOOOOOOOOOOOOOOOOOO<br>OOOOOOOOOOOOOOOOOOOOOOOOOOOOOOOOOOOOOOOOOOOOOOOOOO<br>OOOOOOOOOOOOOOOOOOOOOOOOOOOOOOOOOOOOOOOOOOOOOOOOOO<br>OOOOOOOOOOOOOOOOOOOOOOOOOOOOOOOOOOOOOOOOOOOOOOOOOO<br>OOOOOOOOOOOOOOOOOOOOOOOOOOOOOOOOOOOOOOOOOOOOOOOOOO<br>OOOOOOOOOOOOOO
WP_388050255.1	GLOB	MAGNPAGPIGWGSDLSSLAKGLFGTIEFRSSNLEAFPQWLRVLDELERLD<br>ELIRLCDEENECLNNRFSAWRARVNSLENRHPRAQMDELNRFINEIMPYL<br>EDSRVYGRSDYWAAPQEFLRHSGDCEDYAILKYASLKELGFPEEDMRIVV<br>LEDTLREVPHAVLAVDYEDETFILDNNFSVVLNDDELPQYAPQYSVNATH<br>RWVHISPSSVE	OOOOOOOOOOOOOOOOOOOOOOOOOOOOOOOOOOOOOOOOOOOOOOOOOO<br>OOOOOOOOOOOOOOOOOOOOOOOOOOOOOOOOOOOOOOOOOOOOOOOOOO<br>OOOOOOOOOOOOOOOOOOOOOOOOOOOOOOOOOOOOOOOOOOOOOOOOOO<br>OOOOOOOOOOOOOOOOOOOOOOOOOOOOOOOOOOOOOOOOOOOOOOOOOO<br>OOOOOOOOOOO
WP_234703607.1	GLOB	MRLFNTVAFRGPLKALPKWERVLRENRRNPVLQKQTVSGPDAPAGGRGEG<br>WKSVAARAAGAPPMEKLRMVNAFFNRWPYRLDVEIYGVSDFWATPAEFMR<br>HSGDCEDYAISKYFALRMLGFPADALRIVVLLDSIRGIGYAVLVVFLDAK<br>AYVLDNLSDLVLTHDRYSHYRPQYSVNEEYRWAHIPVRPAATGKAEGKQ	OOOOOOOOOOOOOOOOOOOOOOOOOOOOOOOOOOOOOOOOOOOOOOOOOO<br>OOOOOOOOOOOOOOOOOOOOOOOOOOOOOOOOOOOOOOOOOOOOOOOOOO<br>OOOOOOOOOOOOOOOOOOOOOOOOOOOOOOOOOOOOOOOOOOOOOOOOOO<br>OOOOOOOOOOOOOOOOOOOOOOOOOOOOOOOOOOOOOOOOOOOOOOOOO
WP_207045707.1	SP	MTKTNPMTGARSRFTAIFVALILACSVVAMPSFAEAKAGDLFGYKATPYS<br>GLKPFPKWRKVLATYKDRPGDCVSGKYNRCAHQKWLALIEEWRLLPKEEQ<br>LEKVNRHMNLFRYILDPVNWGVRDYWEIPKEFFARFGDCEDYAIVKYFTL<br>RALGWPAEDMKIVVLQDINLGVAHAILVVKYEGRNLVLDNQIGQVVEATR<br>IRHYRPIYSVNEMGWWRHKR	SSSSSSSSSSSSSSSSSSSSSSSSSSSSSSSSSSSSOOOOOOOOOOOOOO<br>OOOOOOOOOOOOOOOOOOOOOOOOOOOOOOOOOOOOOOOOOOOOOOOOOO<br>OOOOOOOOOOOOOOOOOOOOOOOOOOOOOOOOOOOOOOOOOOOOOOOOOO<br>OOOOOOOOOOOOOOOOOOOOOOOOOOOOOOOOOOOOOOOOOOOOOOOOOO<br>OOOOOOOOOOOOOOOOOOOO
WP_092162492.1	SP	MLFFQSIRAVRATVLCVLFVAFFVIGTEESGFAGSKKVVKQKIFGTVEFK<br>GKISKLPKWTRVLAGMKQWKGYFSSKSKASQTSKAGWKLLKKQIASKPEM<br>DRLRAVNKYFNQWPYRLDKQNYGVTDYWATPIEFLSKSGDCEDYAIAKYY<br>ALKELGFSKDKMRIVAVRDSIRGIGHAVLVVYLSDDVYVLDNQTVLVLSH<br>AKYKHYVPQYSVNENFRWFHVAPAKKTTYKKPINKK	SSSSSSSSSSSSSSSSSSSSSSSSSSSSSSSSSOOOOOOOOOOOOOOOOO<br>OOOOOOOOOOOOOOOOOOOOOOOOOOOOOOOOOOOOOOOOOOOOOOOOOO<br>OOOOOOOOOOOOOOOOOOOOOOOOOOOOOOOOOOOOOOOOOOOOOOOOOO<br>OOOOOOOOOOOOOOOOOOOOOOOOOOOOOOOOOOOOOOOOOOOOOOOOOO<br>OOOOOOOOOOOOOOOOOOOOOOOOOOOOOOOOOOOO

* ProteinID, NCBI sequence identifier; prections (Pred.): GLOB – globular, SP – signal peptide, TM – transmembrane; detailed predictions: S – signal peptide, M – transmembrane helix, O – outside; I – inside.

Supplementary Table 7. Signal peptide prediction likelihood scores for DvhG homologs via SignalP-6.0. *								
ProteinID_species	Prediction	OTHER	SP (Sec/SPI)	LIPO (Sec/SPII)	TAT (Tat/SPI)	TATLIPO (Tat/SPII)	PILIN (Sec/SPIII)	CS Position
WP_066802140.1_Pseudodesulfovibrio	SP	0.0008	0.9983	0.0002	0.0003	0.0002	0.0002	31-32. Pr: 0.9621
WP_156176855.1_Kiloniella spongiae	OTHER	1.0000	0.0000	0.0000	0.0000	0.0000	0.0000	
WP_013513847.1_Pseudodesulfovibrio aespoeensis	SP	0.0105	0.9884	0.0004	0.0003	0.0002	0.0002	33-34. Pr: 0.9239
WP_231038959.1_Nitratidesulfovibrio oxamicus	OTHER	1.0000	0.0000	0.0000	0.0000	0.0000	0.0000	
WP_084264872.1_Sneathiella glossodoripedis	SP	0.0003	0.9990	0.0002	0.0002	0.0002	0.0001	23-24. Pr: 0.7442
WP_018125290.1_Desulfovibrio oxyclinae	SP	0.0027	0.7082	0.2880	0.0005	0.0003	0.0003	32-33. Pr: 0.6692
WP_264983481.1_Pseudodesulfovibrio portus	SP	0.0002	0.9992	0.0001	0.0002	0.0001	0.0001	31-32. Pr: 0.6386
WP_245590754.1_Nitratidesulfovibrio termitidis	OTHER	0.9999	0.0001	0.0000	0.0000	0.0000	0.0000	
WP_176244911.1_Oceanibacterium hippocampi	SP	0.0017	0.9972	0.0003	0.0004	0.0002	0.0002	30-31. Pr: 0.9789
WP_236031536.1_Desulfovibrio legallii	OTHER	0.9999	0.0002	0.0000	0.0000	0.0000	0.0000	
WP_092379318.1_Desulfomicrobium apsheronum	OTHER	1.0000	0.0000	0.0000	0.0000	0.0000	0.0000	
WP_246118364.1_Desulfovibrio piger	OTHER	1.0000	0.0000	0.0000	0.0000	0.0000	0.0000	
WP_006000675.1_Desulfuromonas acetoxidans	LIPO	0.0001	0.0289	0.9710	0.0000	0.0000	0.0000	21-22. Pr: 0.9525
WP_155934525.1_Pseudodesulfovibrio alkaliphilus	SP	0.0007	0.9986	0.0002	0.0002	0.0001	0.0002	32-33. Pr: 0.8869
WP_169726987.1_Desulfovibrio cuneatus	OTHER	0.5271	0.4714	0.0006	0.0004	0.0002	0.0002	
WP_154510627.1_Desulfovibrio porci	LIPO	0.0081	0.0112	0.9806	0.0000	0.0001	0.0000	34-35. Pr: 0.9170
WP_193371556.1_Pelagibius marinus	TAT	0.0000	0.0000	0.0000	1.0000	0.0000	0.0000	32-33. Pr: 0.8703
WP_169543093.1_Sneathiella aquimaris	OTHER	0.5860	0.4115	0.0011	0.0007	0.0003	0.0004	
WP_085901370.1_Kiloniella majae	SP	0.0005	0.9987	0.0003	0.0002	0.0002	0.0002	34-35. Pr: 0.9541
WP_174408948.1_Desulfovibrio psychrotolerans	OTHER	0.9577	0.0382	0.0012	0.0001	0.0001	0.0027	
WP_223294588.1_Nitratidesulfovibrio vulgaris	OTHER	1.0000	0.0000	0.0000	0.0000	0.0000	0.0000	
WP_012805218.1_Desulfomicrobium baculatum	OTHER	1.0000	0.0000	0.0000	0.0000	0.0000	0.0000	
WP_161949025.1_Desulfomicrobium norvegicum	OTHER	1.0000	0.0000	0.0000	0.0000	0.0000	0.0000	
WP_236884816.1_Desulfomicrobium orale	SP	0.0004	0.9990	0.0002	0.0002	0.0001	0.0001	18-19. Pr: 0.9678
WP_281762967.1_Pseudodesulfovibrio nedwellii	SP	0.0002	0.9992	0.0001	0.0002	0.0001	0.0001	23-24. Pr: 0.9740
WP_005027457.1_Bilophila wadsworthia	SP	0.0440	0.9525	0.0022	0.0004	0.0004	0.0004	29-30. Pr: 0.9089

WP_097012996.1_Pseudodesulfovibrio profundus	SP	0.0044	0.9947	0.0003	0.0002	0.0002	0.0002	32-33. Pr: 0.6862
WP_174406921.1_Desulfovibrio subterraneus	OTHER	0.9982	0.0018	0.0000	0.0000	0.0000	0.0000	
WP_183719708.1_Desulfovibrio intestinalis	OTHER	0.9995	0.0002	0.0000	0.0000	0.0000	0.0002	
WP_147818644.1_Salidesulfovibrio onnuriiensis	SP	0.0043	0.9947	0.0003	0.0002	0.0002	0.0002	34-35. Pr: 0.9518
WP_077072032.1_Mailhella massiliensis	SP	0.0336	0.9411	0.0230	0.0014	0.0004	0.0004	36-37. Pr: 0.7985
WP_245628634.1_Salidesulfovibrio brasiliensis	SP	0.0009	0.5948	0.4026	0.0009	0.0004	0.0003	29-30. Pr: 0.5572
WP_015751021.1_Desulfohalobium retbaense	SP	0.0008	0.9985	0.0002	0.0002	0.0002	0.0002	36-37. Pr: 0.9532
WP_251935991.1_Sneathiella marina	SP	0.0006	0.9985	0.0003	0.0002	0.0002	0.0001	29-30. Pr: 0.9542
WP_223299999.1_Desulfovibrio fairfieldensis	OTHER	1.0000	0.0000	0.0000	0.0000	0.0000	0.0000	
WP_169570193.1_Sneathiella limimaris	SP	0.0014	0.8565	0.1413	0.0003	0.0003	0.0002	25-26. Pr: 0.7837
WP_025899687.1_Sneathiella glossodoripedis	SP	0.0003	0.9991	0.0001	0.0002	0.0001	0.0001	33-34. Pr: 0.9749
WP_161315810.1_Sneathiella litorea	SP	0.3119	0.6803	0.0068	0.0003	0.0003	0.0004	30-31. Pr: 0.5326
WP_161338412.1_Sneathiella chungangensis	SP	0.0012	0.9968	0.0012	0.0003	0.0002	0.0002	31-32. Pr: 0.9407
WP_229595160.1_Pseudodesulfovibrio sediminis	SP	0.2126	0.7762	0.0102	0.0004	0.0003	0.0003	24-25. Pr: 0.7303
WP_338667776.1_Pseudodesulfovibrio methanolicus	SP	0.0206	0.8971	0.0794	0.0017	0.0007	0.0005	32-33. Pr: 0.8648
WP_285907339.1_Pseudodesulfovibrio pelocollis	OTHER	1.0000	0.0000	0.0000	0.0000	0.0000	0.0000	
WP_380255403.1_Kiloniella antarctica	OTHER	1.0000	0.0000	0.0000	0.0000	0.0000	0.0000	
WP_148266956.1_Pseudodesulfovibrio mercurii	SP	0.0010	0.9982	0.0002	0.0002	0.0002	0.0002	22-23. Pr: 0.5247
WP_074216486.1_Halodesulfovibrio marinisediminis	OTHER	1.0000	0.0000	0.0000	0.0000	0.0000	0.0000	
WP_161624767.1_Halodesulfovibrio aestuarii	OTHER	1.0000	0.0000	0.0000	0.0000	0.0000	0.0000	
WP_231895534.1_Halodesulfovibrio spirochaetisodalis	SP	0.0114	0.9850	0.0029	0.0002	0.0002	0.0002	25-26. Pr: 0.7184
WP_072697218.1_Desulfovibrio litoralis	OTHER	0.9995	0.0005	0.0000	0.0000	0.0000	0.0000	
WP_169560244.1_Sneathiella chinensis	SP	0.0004	0.9988	0.0003	0.0002	0.0002	0.0002	25-26. Pr: 0.9740
WP_242012421.1_Pseudodesulfovibrio cashew	OTHER	1.0000	0.0000	0.0000	0.0000	0.0000	0.0000	
WP_251935996.1_Sneathiella marina	SP	0.0003	0.9990	0.0002	0.0002	0.0002	0.0002	32-33. Pr: 0.9452
WP_085882215.1_Oceanibacterium hippocampi	SP	0.0078	0.5082	0.4655	0.0163	0.0016	0.0006	30-31. Pr: 0.4921
WP_161338416.1_Sneathiella chungangensis	SP	0.0002	0.9991	0.0002	0.0002	0.0001	0.0001	32-33. Pr: 0.9650
WP_169543097.1_Sneathiella aquimaris	SP	0.0003	0.9990	0.0002	0.0002	0.0002	0.0001	25-26. Pr: 0.9686
WP_051307240.1_Desulfomicrobium escambiense	OTHER	0.5796	0.3752	0.0390	0.0024	0.0013	0.0024	

WP_071544367.1_Pseudodesulfovibrio hydrargyri	SP	0.0381	0.8218	0.0914	0.0412	0.0064	0.0011	33-34. Pr: 0.7836
WP_015416032.1_Pseudodesulfovibrio piezophilus	OTHER	0.5514	0.3769	0.0693	0.0010	0.0005	0.0009	
WP_161315807.1_Sneathiella litorea	SP	0.0002	0.9992	0.0001	0.0001	0.0001	0.0001	23-24. Pr: 0.9800
WP_167230619.1_Pelagibius litoralis	TAT	0.0000	0.0000	0.0000	1.0000	0.0000	0.0000	32-33. Pr: 0.7942
WP_081649842.1_Fodinicurvata sediminis	OTHER	1.0000	0.0000	0.0000	0.0000	0.0000	0.0000	
WP_142895221.1_Denitrobaculum tricleocarpae	OTHER	1.0000	0.0000	0.0000	0.0000	0.0000	0.0000	
WP_243544993.1_Pseudodesulfovibrio tunisiensis	LIPO	0.1807	0.0701	0.7480	0.0003	0.0003	0.0005	25-26. Pr: 0.5478
WP_169560248.1_Sneathiella chinensis	SP	0.0003	0.9990	0.0001	0.0002	0.0002	0.0001	33-34. Pr: 0.9699
WP_265826327.1_Desulfovibrio mangrovi	OTHER	0.8586	0.1222	0.0014	0.0007	0.0004	0.0166	
WP_245622974.1_Kiloniella litopenaei	OTHER	1.0000	0.0000	0.0000	0.0000	0.0000	0.0000	
WP_371384792.1_Pseudodesulfovibrio karagichevae	SP	0.1110	0.8878	0.0002	0.0004	0.0003	0.0003	21-22. Pr: 0.8096
WP_279521168.1_Pseudodesulfovibrio thermohalotolerans	OTHER	0.6007	0.3972	0.0010	0.0004	0.0003	0.0004	
WP_162175094.1_Fodinicurvata fenggangensis	OTHER	1.0000	0.0000	0.0000	0.0000	0.0000	0.0000	
WP_207045699.1_Sneathiella sedimenti	SP	0.0011	0.9977	0.0005	0.0003	0.0002	0.0002	31-32. Pr: 0.6855
WP_020591926.1_Kiloniella laminariae	SP	0.0002	0.9991	0.0002	0.0002	0.0002	0.0001	27-28. Pr: 0.9734
WP_169570190.1_Sneathiella limimaris	SP	0.0004	0.9989	0.0002	0.0002	0.0002	0.0002	23-24. Pr: 0.5139
WP_193370322.1_Pelagibius marinus	SP	0.0003	0.9991	0.0001	0.0002	0.0001	0.0001	24-25. Pr: 0.9584
WP_245170854.1_Desulfovibrio desulfuricans	OTHER	1.0000	0.0000	0.0000	0.0000	0.0000	0.0000	
WP_407844443.1_Desulfovibrio falkowii	OTHER	0.5559	0.4413	0.0020	0.0003	0.0002	0.0003	
WP_388050255.1_Fodinicurvata halophila	OTHER	1.0000	0.0000	0.0000	0.0000	0.0000	0.0000	
WP_234703607.1_Oleidesulfovibrio alaskensis	OTHER	1.0000	0.0000	0.0000	0.0000	0.0000	0.0000	
WP_207045707.1_Sneathiella sedimenti	SP	0.0076	0.9455	0.0440	0.0023	0.0004	0.0003	36-37. Pr: 0.8947
WP_092162492.1_Maridesulfovibrio ferrireducens	SP	0.4246	0.5716	0.0026	0.0004	0.0003	0.0005	33-34. Pr: 0.1957

* SP – secretory signal peptide (Sec/SPI), LIPO – lipoprotein signal peptide (Sec/SPII), TAT – TAT signal peptide (Tat/SPI), TATLIPO – TAT lipoprotein signal peptide (Tat/SPII), PILIN – Pilin-like signal peptide (Sec/SPIII), CS position – cleavage site position (residues and probability).

Supplementary Table 8. Resource table.

**Cells**		
T7 Express *E. coli* BL21(DE3)	*fhuA2 lacZ::T7 gene1 [lon] ompT gal sulA11 R(mcr-73::miniTn10*--Tet^S*)2 [dcm] R(zgb-210::Tn10*--Tet^S*) endA1 Δ(mcrC-mrr)114::IS10*	NEB
NEB 5-alpha	*fhuA2Δ(argF-lacZ)U169 phoA glnV44 Φ80Δ(lacZ)M15 gyrA96 recA1 relA1 endA1 thi-1 hsdR17*	NEB
**Plasmids**		
pET28	T7 promotor/terminator, f1 origin, pBR322 origin, *lacI*, Kan^R	Novagen
pET21	T7 promotor/terminator, f1 origin, pBR322 origin, *lacI*, Amp^R	Novagen
pCDFDuet	T7 promotor/term., CDF origin, *lacI*, *lacI*, Sm^R	Novagen
pET28-LapD-msfGFP-His_6		[9]
pET28-DvhD-msfGFP-His_6		this study
pET28-LapG-msfGFP-His_6		this study
pET28-His_6-SUMO-LapG		[1]
pET28-DvhG-msfGFP-His_6		this study
pCDF-Duet-DvhG-StrepTagII		this study
pCDF-Duet-DvhG-StrepTagII $D^{316}A/E^{318}A$		this study
pCDF-Duet-DvhG-StrepTagII $C^{317}A$		this study
pET28-His6-SUMO-$LapA^{81-131}$-msfGFP	pCleevR-$LapA^{81-131}$	[10]
pET28-His6-SUMO-$DvhA^{79-129}$-msfGFP	pCleevR-$DvhA^{79-129}$	this study
pET28-His6-SUMO-$DvhA^{79-129}$-msfGFP PRRG	pCleevR-$DvhA^{79-129}$ PRRG mutant	this study
pET28-His6-SUMO-$DvhA^{1-129}$-msfGFP	pCleevR-$DvhA^{1-129}$	this study
pET28-His6-SUMO-$DvhA^{1-129}$-msfGFP $L^{90}E$	pCleevR-$DvhA^{1-129}$ $L^{90}E$	this study
pET28-His6-SUMO-$DvhA^{1-129}$-msfGFP $I^{85}E$	pCleevR-$DvhA^{1-129}$ $I^{85}E$	this study
pET28-His6-SUMO-$DvhA^{1-129}$-msfGFP $F^{95}E$	pCleevR-$DvhA^{1-129}$ $F^{95}E$	this study
pET28-His6-SUMO-$DVU1545^{59-111}$-msfGFP	pCleevR-$DVU1545^{59-111}$	this study
pET28-His6-SUMO-$DVU1545^{59-111}$-msfGFP PRRG	pCleevR-$DVU1545^{59-111}$ PRRG mutant	this study
pET28-His6-SUMO-$DVU1545^{1-111}$-msfGFP	pCleevR-$DVU1545^{1-111}$	this study
pET28-His_6-SUMO-$DvhD^{57-327}$	DvhD periplasmic domain	this study
pET28-His_6-SUMO-$DvhD^{491-701}$	DvhD HD-GYP domain	this study
pET28-His_6-SUMO-$DvhD^{491-701}$ $M^{646}A$		this study
pET28-His_6-SUMO-$DvhD^{491-701}$ $R^{650}A$		this study
pET28-His_6-SUMO-$DvhD^{491-701}$ $Y^{652}A/R^{653}A$		this study
pET28-His_6-SUMO-$DvhD^{491-701}$ $P^{567}T$		this study
pET28-His_6-SUMO-$DvhD^{491-701}$ $K^{520}E$		this study
pET28-His_6-SUMO-$DvhD^{491-701}$ $K^{520}E/M^{646}A$		this study
pET28-His_6-SUMO-$DvhD^{374-701}$	DvhD PAS/HD-GYP domains	this study
pET21-WspR	*P. aeruginosa* WspR full-length	[11]
pET21-RocR	*P. aeruginosa* RocR/SadR full-length	This study
**Key reagents**		
InFusion HD EcoDry cloning kit		Takara Bio
Quikchange II site directed mutagenesis		Agilent
NucleoSpin Plasmid Mini kit		Macherey-Nagel
NiNTA Superflow		Qiagen
HiPrep 26/10 Desalting colum		Cytiva
HiLoad 16/600 Superdex 200 pg		Cytiva
Nucleotides		Jena Bioscience
Nucleotides		Biolog
Gemini 3µm C18 (150 x 4.6 mm)		Phenomenex
**Services**		
Sanger sequencing		Microsynth
DNA primer		Eurofins
DNA fragments (codon-optimized)		Eurofins
DNA fragments (codon-optimized)		GeneArt
Gene synthesis and cloning		GenScript

Software		
XDS		[12]
Phenix		[13]
Coot		[14]
Pymol Molecular Graphics System		Schrödinger, LLC
ChimeraX		[15,16]
Prism		GraphPad
ImageJ/Fiji		[17]
Servers, web sites, databases		
ColabFold		[18]
BioRender		-
RCSB		[19]
Foldseek		[20]
FoldMason		[21]
Clustal Omega		[22]
BLASTp		[23,24]
ConSurf web server		[25]
DeepTMHMM - 1.0		[26]
SignalP - 6.0		[27]
AF2 models		
DvhG full-length	AF2/ColabFold	this study
DvhA (segments spanning full-length)	AF2/ColabFold	this study
DVU1545 (segments spanning full-length)	AF2/ColabFold	this study
*Pfl*_LapG•LapA (88-128)	AF2/ColabFold	this study
*Pfl*_LapG•MapA (83-131)	AF2/ColabFold	this study
*Vc*_LapG•CraA (100-141)	AF2/ColabFold	this study
*Vc*_LapG•FrhA (64-110)	AF2/ColabFold	this study
DvhG (210-399)•DvhA (1-110)	AF2/ColabFold	this study
DvhG (210-399)•DVU1545 (1-91)	AF2/ColabFold	this study
DvhD full-length, dimer	AF2/ColabFold	this study
DvhD dCache • DvhG BTLCP domain	AF3	this study
Crystallographic models		
DvhD dCache	PDB code 9RC2	this study
DvhD PAS/HD-GYP	PDB code 9RC0	this study
DvhD HD-GYP•c-di-GMP	PDB code 9RBZ	this study

Supplemenary Table 9. Protein sequences of proteolysis reporter constructs. *

pCleevR-DvhA$^{1-129}$	MGSSHHHHHHSSGLVPRGSHMASMSDSEVNQEAKPEVKPEVKPETHINLKVSDGSSEIFF<br>KIKKTTPLRRLMEAFAKRQGKEMDSLRFLYDGIRIQADQTPEDLDMEDNDIIEAHREQIG<br>GS**MPLNRTITPQQATAGTIRLPAPAVDEVITIQNAAGLKLALEFAPDAATTEKSGNDLVF**<br>**TFPEGGQVIVSDFFAQLEGGNVPTFVIEGQELPGDAFLTAFNAELLPAAGPGAGGGAGSG**<br>***GVGDYTDDPGN***GTSGTS*SKGEELFTGVVPILVELDGDVNGHKFSVRGEGEGDATNGKLTL*<br>*KFICTTGKLPVPWPTLVTTLTYGVQCFSRYPDHMKRHDFFKSAMPEGYVQERTISFKDDG*<br>*TYKTRAEVKFEGDTLVNRIELKGIDFKEDGNILGHKLEYNFNSHNVYITADKQKNGIKAN*<br>*FKIRHNVEDGSVQLADHYQQNTPIGDGPVLLPDNHYLSTQSKLSKDPNEKRDHMVLLEFV*<br>*TARGITHGMDELYKGS*
pCleevR-DvhA$^{79-129}$	MGSSHHHHHHSSGLVPRGSHMASMSDSEVNQEAKPEVKPEVKPETHINLKVSDGSSEIFF<br>KIKKTTPLRRLMEAFAKRQGKEMDSLRFLYDGIRIQADQTPEDLDMEDNDIIEAHREQIG<br>GS**VPTFVIEGQELPGDAFLTAFNAELLPAAGPGAGGGAGSGGVGDYTDDPGN**GTSGTS*SK*<br>*GEELFTGVVPILVELDGDVNGHKFSVRGEGEGDATNGKLTLKFICTTGKLPVPWPTLVTT*<br>*LTYGVQCFSRYPDHMKRHDFFKSAMPEGYVQERTISFKDDGTYKTRAEVKFEGDTLVNRI*<br>*ELKGIDFKEDGNILGHKLEYNFNSHNVYITADKQKNGIKANFKIRHNVEDGSVQLADHYQ*<br>*QNTPIGDGPVLLPDNHYLSTQSKLSKDPNEKRDHMVLLEFVTARGITHGMDELYKGS*
>pCleevR-DVU1545$^{1-111}$	MGSSHHHHHHSSGLVPRGSHMASMSDSEVNQEAKPEVKPEVKPETHINLKVSDGSSEIFF<br>KIKKTTPLRRLMEAFAKRQGKEMDSLRFLYDGIRIQADQTPEDLDMEDNDIIEAHREQIG<br>GS**MPARTVEQHHSVAGAARILLDFPTDAATIEREGDALVFNFPDGARLVLDGFYTVTDGA**<br>**ELPDFILPDGTSFPGYDFLAAIDAELLPAAGPGAGGGSGAGGGVGEYDDDAGR**GTSGTS*S*<br>*KGEELFTGVVPILVELDGDVNGHKFSVRGEGEGDATNGKLTLKFICTTGKLPVPWPTLVT*<br>*TLTYGVQCFSRYPDHMKRHDFFKSAMPEGYVQERTISFKDDGTYKTRAEVKFEGDTLVNR*<br>*IELKGIDFKEDGNILGHKLEYNFNSHNVYITADKQKNGIKANFKIRHNVEDGSVQLADHY*<br>*QQNTPIGDGPVLLPDNHYLSTQSKLSKDPNEKRDHMVLLEFVTARGITHGMDELYKGS*
pCleevR-DVU1545$^{59-111}$	MGSSHHHHHHSSGLVPRGSHMASMSDSEVNQEAKPEVKPEVKPETHINLKVSDGSSEIFF<br>KIKKTTPLRRLMEAFAKRQGKEMDSLRFLYDGIRIQADQTPEDLDMEDNDIIEAHREQIG<br>TGSTGS**ELPDFILPDGTSFPGYDFLAAIDAELLPAAGPGAGGGSGAGGGVGEYDDDAGR**G<br>TSGTS*SKGEELFTGVVPILVELDGDVNGHKFSVRGEGEGDATNGKLTLKFICTTGKLPVP*<br>*WPTLVTTLTYGVQCFSRYPDHMKRHDFFKSAMPEGYVQERTISFKDDGTYKTRAEVKFEG*<br>*DTLVNRIELKGIDFKEDGNILGHKLEYNFNSHNVYITADKQKNGIKANFKIRHNVEDGSV*<br>*QLADHYQQNTPIGDGPVLLPDNHYLSTQSKLSKDPNEKRDHMVLLEFVTARGITHGMDEL*<br>*YKGS*

* Underlined: His_6-SUMO; bold: insert; italic: msfGFP.

Supplementary References

1. Chatterjee, D. *et al.* Mechanistic insight into the conserved allosteric regulation of periplasmic proteolysis by the signaling molecule cyclic-di-GMP. *eLife* **3**, e03650 (2014).

2. Chatterjee, D., Boyd, C. D., O'Toole, G. A. & Sondermann, H. Structural characterization of a conserved, calcium-dependent periplasmic protease from *Legionella pneumophila*. *J. Bacteriol.* **194**, 4415–4425 (2012).

3. Wu, R. *et al.* Insight into the sporulation phosphorelay: Crystal structure of the sensor domain of *Bacillus subtilis* histidine kinase, KinD. *Protein Sci.* **22**, 564–576 (2013).

4. Robinson, C. D. *et al.* Host-emitted amino acid cues regulate bacterial chemokinesis to enhance colonization. *Cell Host Microbe* **29**, 1221-1234.e8 (2021).

5. Gavira, J. A. *et al.* How Bacterial chemoreceptors evolve novel ligand specificities. *mBio* **11**, 10.1128/mbio.03066-19 (2020).

6. Matilla, M. A. *et al.* Chemotaxis of the human pathogen *Pseudomonas aeruginosa* to the neurotransmitter acetylcholine. *mBio* **13**, e03458-21 (2022).

7. Monteagudo-Cascales, E. *et al.* Ubiquitous purine sensor modulates diverse signal transduction pathways in bacteria. *Nat. Commun.* **15**, 5867 (2024).

8. Bellini, D. *et al.* Crystal structure of an HD-GYP domain cyclic-di-GMP phosphodiesterase reveals an enzyme with a novel trinuclear catalytic iron centre. *Mol. Microbiol.* **91**, 26–38 (2014).

9. Cooley, R. B., O'Donnell, J. P. & Sondermann, H. Coincidence detection and bi-directional transmembrane signaling control a bacterial second messenger receptor. *eLife* **5**, e21848 (2016).

10. Cooley, R. B. *et al.* Cyclic di-GMP-regulated periplasmic proteolysis of a *Pseudomonas aeruginosa* type Vb secretion system substrate. *J. Bacteriol.* **198**, 66–76 (2015).

11. De, N. *et al.* Phosphorylation-independent regulation of the diguanylate cyclase WspR. *PLoS Biol.* **6**, e67 (2008).

12. Kabsch, W. XDS. *Acta Crystallogr. Sect. D, Biol. Crystallogr.* **66**, 125–32 (2009).

13. Liebschner, D. *et al.* Macromolecular structure determination using X-rays, neutrons and electrons: recent developments in Phenix. *Acta Crystallogr. Sect. D* **75**, 861–877 (2019).

14. Emsley, P., Lohkamp, B., Scott, W. G. & Cowtan, K. Features and development of Coot. *Acta Crystallogr. Sect. D* **66**, 486–501 (2010).

15. Pettersen, E. F. *et al.* UCSF ChimeraX: Structure visualization for researchers, educators, and developers. *Protein Sci.* **30**, 70–82 (2021).

16. Meng, E. C. *et al.* UCSF ChimeraX: Tools for structure building and analysis. *Protein Sci.* **32**, e4792 (2023).

17. Schindelin, J. *et al.* Fiji: an open-source platform for biological-image analysis. *Nat. Methods* **9**, 676–682 (2012).

18. Mirdita, M. *et al.* ColabFold: making protein folding accessible to all. *Nat. methods* **19**, 679–682 (2022).

19. Berman, H. M. *et al.* The Protein Data Bank. *Nucleic Acids Res.* **28**, 235–242 (2000).

20. Kempen, M. van *et al.* Fast and accurate protein structure search with Foldseek. *Nat. Biotechnol.* **42**, 243–246 (2024).

21. Gilchrist, C. L. M., Mirdita, M. & Steinegger, M. Multiple protein structure alignment at scale with FoldMason. *bioRxiv* 2024.08.01.606130 (2024) doi:10.1101/2024.08.01.606130.

22. Sievers, F. & Higgins, D. G. Clustal Omega for making accurate alignments of many protein sequences. *Protein Sci.* **27**, 135–145 (2018).

23. Altschul, S. F., Gish, W., Miller, W., Myers, E. W. & Lipman, D. J. Basic local alignment search tool. *J. Mol. Biol.* **215**, 403–410 (1990).

24. Sayers, E. W. *et al.* Database resources of the National Center for Biotechnology Information in 2025. *Nucleic Acids Res.* **53**, D20–D29 (2024).

25. Yariv, B. *et al.* Using evolutionary data to make sense of macromolecules with a “face-lifted” ConSurf. *Protein Sci. : A Publ. Protein Soc.* **32**, e4582 (2023).

26. Hallgren, J. *et al.* DeepTMHMM predicts alpha and beta transmembrane proteins using deep neural networks. (2022) doi:10.1101/2022.04.08.487609.

27. Teufel, F. *et al.* SignalP 6.0 predicts all five types of signal peptides using protein language models. *Nat. Biotechnol.* **40**, 1023–1025 (2022).
