# Supplementary material for: Structural analyses uncover protease-adhesin interactions and c-di-GMP receptor regulation in sulfate-reducing bacteria
Source: Nat Commun. 2026 Apr 17;17:3564. doi: 10.1038/s41467-026-71936-5 (PMC13090378; doi:10.1038/s41467-026-71936-5)
Supplement: Supplementary file 2 — Description of Additional Supplementary Files [file 41467_2026_71936_MOESM2_ESM.pdf]

Description of Additional Supplementary Files

File name: Supplementary Data 1
Description: WebFlags analysis of DvhDG-containing gene neighborhoods.

File name: Supplementary Data 2
Description: Alphafold model summary.
