# Supplementary material for: Structural analyses uncover protease-adhesin interactions and c-di-GMP receptor regulation in sulfate-reducing bacteria
Source: Nat Commun. 2026 Apr 17;17:3564. doi: 10.1038/s41467-026-71936-5 (PMC13090378; doi:10.1038/s41467-026-71936-5)
Supplement: Supplementary file 3 — Supplementary Data 1 [file 41467_2026_71936_MOESM3_ESM.pdf]

Structural analyses uncover protease-adhesin interactions and c-di-GMP receptor regulation in sulfate-reducing bacteria

Maria E. Font, Amruta A. Karbelkar, Justin D. Lormand, Sofia Mortensen, Maria J. Garcia-Garcia, George A. O'Toole, Holger Sondermann

Supplementary Dataset 1

WebFlags analysis of DvhDG-containing gene neighborhoods

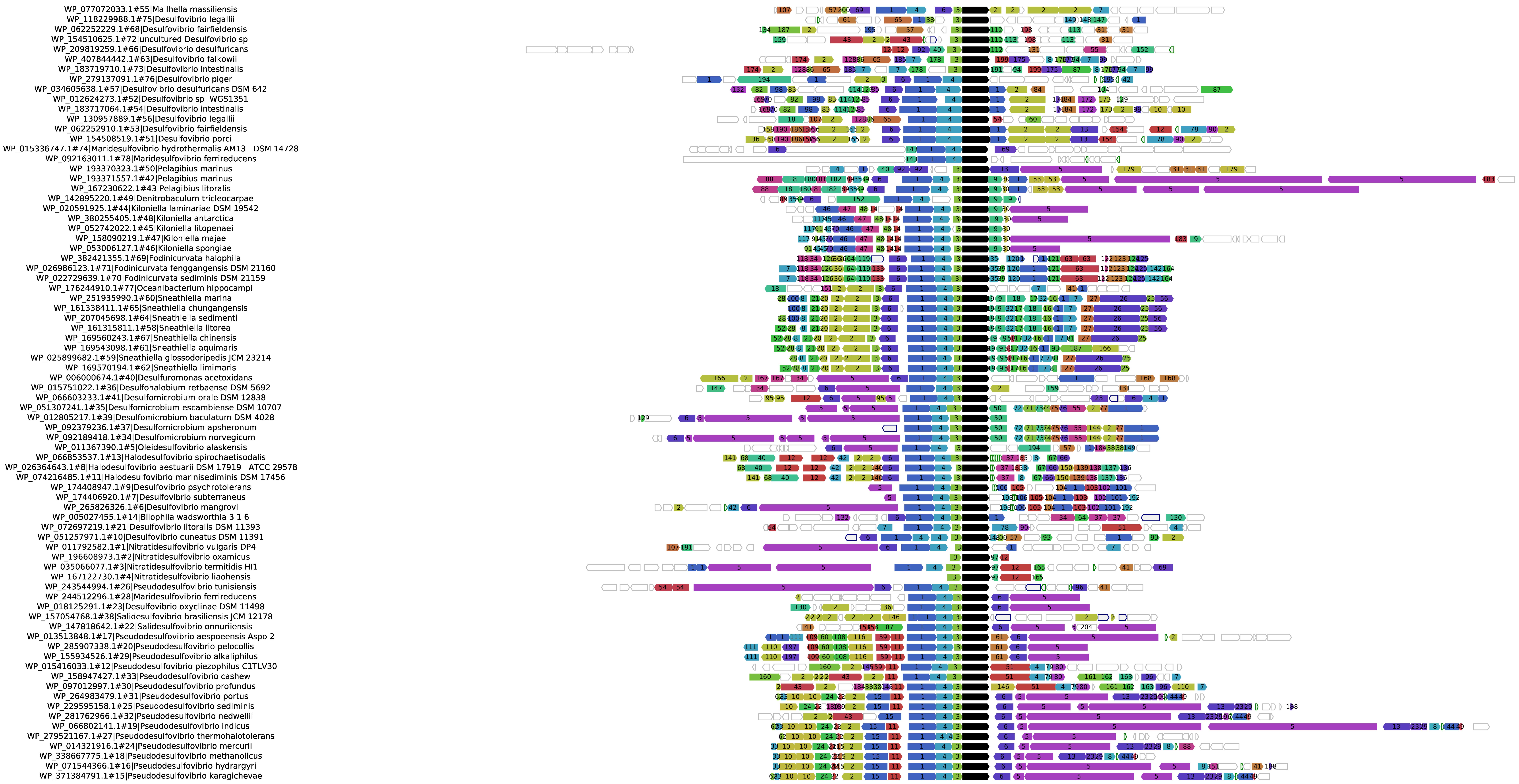

WP_077072033.1#55|Mailhella massiliensis
WP_118229988.1#75|Desulfovibrio legallii
WP_062252229.1#68|Desulfovibrio fairfieldensis
WP_154510625.1#72|uncultured Desulfovibrio sp
WP_209819259.1#66|Desulfovibrio desulfuricans
WP_407844442.1#63|Desulfovibrio falkowii
WP_183719710.1#73|Desulfovibrio intestinalis
WP_279137091.1#76|Desulfovibrio piger
WP_034605638.1#57|Desulfovibrio desulfuricans DSM 642
WP_012624273.1#52|Desulfovibrio sp WGS1351
WP_183717064.1#54|Desulfovibrio intestinalis
WP_130957889.1#56|Desulfovibrio legallii
WP_062252910.1#53|Desulfovibrio fairfieldensis
WP_154508519.1#51|Desulfovibrio porci
WP_015336747.1#74|Maridesulfovibrio hydrothermalis AM13 DSM 14728
WP_092163011.1#78|Maridesulfovibrio ferrireducens
WP_193370323.1#50|Pelagibius marinus
WP_193371557.1#42|Pelagibius marinus
WP_167230622.1#43|Pelagibius litoralis
WP_142895220.1#49|Denitrobaculum tricleocarpae
WP_020591925.1#44|Kiloniella laminariae DSM 19542
WP_380255405.1#48|Kiloniella antarctica
WP_052742022.1#45|Kiloniella litopenaei
WP_158090219.1#47|Kiloniella majae
WP_053006127.1#46|Kiloniella spongiae
WP_382421355.1#69|Fodinicurvata halophila
WP_026986123.1#71|Fodinicurvata fenggangensis DSM 21160
WP_022729639.1#70|Fodinicurvata sediminis DSM 21159
WP_176244910.1#77|Oceanibacterium hippocampi
WP_251935990.1#60|Sneathiella marina
WP_161338411.1#65|Sneathiella chungangensis
WP_207045698.1#64|Sneathiella sedimenti
WP_161315811.1#58|Sneathiella litorea
WP_169560243.1#67|Sneathiella chinensis
WP_169543098.1#61|Sneathiella aquimaris
WP_025899682.1#59|Sneathiella glossodoripedis JCM 23214
WP_169570194.1#62|Sneathiella limimaris
WP_006000674.1#40|Desulfuromonas acetoxidans
WP_015751022.1#36|Desulfohalobium retbaense DSM 5692
WP_066603233.1#41|Desulfomicrobium orale DSM 12838
WP_051307241.1#35|Desulfomicrobium escambiense DSM 10707
WP_012805217.1#39|Desulfomicrobium baculatum DSM 4028
WP_092379236.1#37|Desulfomicrobium apsheronum
WP_092189418.1#34|Desulfomicrobium norvegicum
WP_011367390.1#5|Oleidesulfovibrio alaskensis
WP_066853537.1#13|Halodesulfovibrio spirochaetisodalis
WP_026364643.1#8|Halodesulfovibrio aestuarii DSM 17919 ATCC 29578
WP_074216485.1#11|Halodesulfovibrio marinisediminis DSM 17456
WP_174408947.1#9|Desulfovibrio psychrotolerans
WP_174406920.1#7|Desulfovibrio subterraneus
WP_265826326.1#6|Desulfovibrio mangrovi
WP_005027455.1#14|Bilophila wadsworthia 3 1 6
WP_072697219.1#21|Desulfovibrio litoralis DSM 11393
WP_051257971.1#10|Desulfovibrio cuneatus DSM 11391
WP_011792582.1#1|Nitratidesulfovibrio vulgaris DP4
WP_196608973.1#2|Nitratidesulfovibrio oxamicus
WP_035066077.1#3|Nitratidesulfovibrio termitidis HI1
WP_167122730.1#4|Nitratidesulfovibrio liaohensis
WP_243544994.1#26|Pseudodesulfovibrio tunisiensis
WP_244512296.1#28|Maridesulfovibrio ferrireducens
WP_018125291.1#23|Desulfovibrio oxyclinae DSM 11498
WP_157054768.1#38|Salidesulfovibrio brasiliensis JCM 12178
WP_147818642.1#22|Salidesulfovibrio onnuriiensis
WP_013513848.1#17|Pseudodesulfovibrio aespoeensis Aspo 2
WP_285907338.1#20|Pseudodesulfovibrio pelocollis
WP_155934526.1#29|Pseudodesulfovibrio alkaliphilus
WP_015416033.1#12|Pseudodesulfovibrio piezophilus C1TLV30
WP_158947427.1#33|Pseudodesulfovibrio cashew
WP_097012997.1#30|Pseudodesulfovibrio profundus
WP_264983479.1#31|Pseudodesulfovibrio portus
WP_229595158.1#25|Pseudodesulfovibrio sediminis
WP_281762966.1#32|Pseudodesulfovibrio nedwellii
WP_066802141.1#19|Pseudodesulfovibrio indicus
WP_279521167.1#27|Pseudodesulfovibrio thermohalotolerans
WP_014321916.1#24|Pseudodesulfovibrio mercurii
WP_338667775.1#18|Pseudodesulfovibrio methanolicus
WP_071544366.1#16|Pseudodesulfovibrio hydrargyri
WP_371384791.1#15|Pseudodesulfovibrio karagichevae

1(1)	WP_028578378.1	WP_028578378.1	type I secretion system permease/ATPase
1(1)	WP_388050252.1	WP_388050252.1	hypothetical protein
1(1)	WP_035069860.1	WP_035069860.1	type I secretion system permease/ATPase
1(1)	WP_265826320.1	WP_265826320.1	ABC transporter ATP-binding protein
1(1)	WP_206376368.1	WP_206376368.1	type I secretion system permease/ATPase
1(1)	WP_066603256.1	WP_066603256.1	ABC transporter ATP-binding protein
1(1)	WP_066853458.1	WP_066853458.1	type I secretion system permease/ATPase
1(1)	WP_154508523.1	WP_154508523.1	type I secretion system permease/ATPase
1(1)	WP_161315818.1	WP_161315818.1	ABC transporter ATP-binding protein
1(1)	WP_118229976.1	WP_118229976.1	ABC transporter ATP-binding protein
1(1)	WP_018125288.1	WP_018125288.1	type I secretion system permease/ATPase
1(1)	WP_265826329.1	WP_265826329.1	type I secretion system permease/ATPase
1(1)	WP_084650651.1	WP_084650651.1	type I secretion system permease/ATPase
1(1)	WP_246387909.1	WP_246387909.1	type I secretion system permease/ATPase
1(1)	WP_371384824.1	WP_371384824.1	type I secretion system permease/ATPase
1(1)	WP_092163009.1	WP_092163009.1	ATP-binding cassette domain-containing protein
1(1)	WP_020000216.1	WP_020000216.1	type I secretion system permease/ATPase
1(1)	WP_022729637.1	WP_022729637.1	type I secretion system permease/ATPase
1(1)	WP_092379266.1	WP_092379266.1	excinuclease ABC subunit UvrA
1(1)	WP_279137087.1	WP_279137087.1	type I secretion system permease/ATPase
1(1)	WP_015416030.1	WP_015416030.1	type I secretion system permease/ATPase
1(1)	WP_169726988.1	WP_169726988.1	iron chelate uptake ABC transporter family permease subunit
1(1)	WP_035066087.1	WP_035066087.1	ABC transporter ATP-binding protein
1(1)	WP_071544519.1	WP_071544519.1	type I secretion system permease/ATPase
1(1)	WP_022659516.1	WP_022659516.1	type I secretion system permease/ATPase
1(1)	WP_174406914.1	WP_174406914.1	ABC transporter ATP-binding protein
1(1)	WP_169543105.1	WP_169543105.1	ABC transporter ATP-binding protein
1(1)	WP_251935980.1	WP_251935980.1	ABC transporter ATP-binding protein
1(1)	WP_012624271.1	WP_012624271.1	type I secretion system permease/ATPase
1(1)	WP_016361059.1	WP_016361059.1	MULTISPECIES: type I secretion system permease/ATPase
1(1)	WP_012805220.1	WP_012805220.1	type I secretion system permease/ATPase
1(1)	WP_217807797.1	WP_217807797.1	type I secretion system permease/ATPase
1(1)	WP_430709106.1	WP_430709106.1	type I secretion system permease/ATPase
1(1)	WP_245628633.1	WP_245628633.1	type I secretion system permease/ATPase
1(1)	WP_013513845.1	WP_013513845.1	MULTISPECIES: type I secretion system permease/ATPase
1(1)	WP_245628632.1	WP_245628632.1	cysteine peptidase family C39 domain-containing protein
1(1)	WP_092189442.1	WP_092189442.1	excinuclease ABC subunit UvrA
1(1)	WP_167230631.1	WP_167230631.1	glycosyltransferase family 4 protein
1(1)	WP_083717135.1	WP_083717135.1	type I secretion system permease/ATPase
1(1)	WP_169560236.1	WP_169560236.1	ABC transporter ATP-binding protein
1(1)	WP_205944922.1	WP_205944922.1	type I secretion system permease/ATPase
1(1)	WP_174406923.1	WP_174406923.1	type I secretion system permease/ATPase
1(1)	WP_092189412.1	WP_092189412.1	MULTISPECIES: type I secretion system permease/ATPase
1(1)	WP_014321919.1	WP_014321919.1	type I secretion system permease/ATPase
1(1)	WP_026986127.1	WP_026986127.1	glycosyltransferase
1(1)	WP_279137083.1	WP_279137083.1	glycosyltransferase
1(1)	WP_028578388.1	WP_028578388.1	excinuclease ABC subunit UvrA
1(1)	WP_011367397.1	WP_011367397.1	amino acid ABC transporter ATP-binding protein
1(1)	WP_388050258.1	WP_388050258.1	type I secretion system permease/ATPase
1(1)	WP_193371560.1	WP_193371560.1	glycosyltransferase family 4 protein
1(1)	WP_006000666.1	WP_006000666.1	excinuclease ABC subunit UvrA
1(1)	WP_169570200.1	WP_169570200.1	ATP-binding cassette domain-containing protein
1(1)	WP_207045703.1	WP_207045703.1	type I secretion system permease/ATPase
1(1)	WP_242621197.1	WP_242621197.1	type I secretion system permease/ATPase
1(1)	WP_026986121.1	WP_026986121.1	type I secretion system permease/ATPase
1(1)	WP_052742019.1	WP_052742019.1	type I secretion system permease/ATPase
1(1)	WP_342663664.1	WP_342663664.1	type I secretion system permease/ATPase
1(1)	WP_202526244.1	WP_202526244.1	type I secretion system permease/ATPase
1(1)	WP_147818648.1	WP_147818648.1	type I secretion system permease/ATPase
1(1)	WP_066603224.1	WP_066603224.1	type I secretion system permease/ATPase
1(1)	WP_013513837.1	WP_013513837.1	MULTISPECIES: iron ABC transporter permease
1(1)	WP_422394423.1	WP_422394423.1	type I secretion system permease/ATPase
1(1)	WP_431194128.1	WP_431194128.1	type I secretion system permease/ATPase
1(1)	WP_246387911.1	WP_246387911.1	glycosyltransferase
1(1)	WP_142895224.1	WP_142895224.1	type I secretion system permease/ATPase
1(1)	WP_169543095.1	WP_169543095.1	type I secretion system permease/ATPase
1(1)	WP_207045679.1	WP_207045679.1	ABC transporter ATP-binding protein
1(1)	WP_322096518.1	WP_322096518.1	ABC transporter ATP-binding protein
1(1)	WP_092379230.1	WP_092379230.1	type I secretion system permease/ATPase
1(1)	WP_174408950.1	WP_174408950.1	type I secretion system permease/ATPase
1(1)	WP_062252908.1	WP_062252908.1	glycosyltransferase

```
1(1)    WP_244512297.1 WP_244512297.1 type I secretion system permease/ATPase
1(1)    WP_285907341.1 WP_285907341.1 type I secretion system permease/ATPase
1(1)    WP_081428092.1 WP_081428092.1 type I secretion system permease/ATPase
1(1)    WP_035066084.1 WP_035066084.1 ABC transporter ATP-binding protein
1(1)    WP_047765663.1 WP_047765663.1 type I secretion system permease/ATPase
1(1)    WP_279137077.1 WP_279137077.1 ATP-binding cassette domain-containing protein
1(1)    WP_251935993.1 WP_251935993.1 type I secretion system permease/ATPase
1(1)    WP_062254822.1 WP_062254822.1 type I secretion system permease/ATPase
1(1)    WP_161338404.1 WP_161338404.1 ABC transporter ATP-binding protein
1(1)    WP_097012994.1 WP_097012994.1 type I secretion system permease/ATPase
1(1)    WP_027180960.1 WP_027180960.1 glycosyltransferase
1(1)    WP_066807092.1 WP_066807092.1 type I secretion system permease/ATPase
1(1)    WP_025899676.1 WP_025899676.1 ABC transporter ATP-binding protein
1(1)    WP_085901372.1 WP_085901372.1 type I secretion system permease/ATPase
1(1)    WP_206378396.1 WP_206378396.1 type I secretion system permease/ATPase
1(1)    WP_380255399.1 WP_380255399.1 type I secretion system permease/ATPase
1(1)    WP_169560246.1 WP_169560246.1 type I secretion system permease/ATPase
1(1)    WP_154508517.1 WP_154508517.1 glycosyltransferase
1(1)    WP_158950841.1 WP_158950841.1 type I secretion system permease/ATPase
1(1)    WP_084539447.1 WP_084539447.1 type I secretion system permease/ATPase
1(1)    WP_174408941.1 WP_174408941.1 ABC transporter ATP-binding protein
1(1)    WP_010938321.1 WP_010938321.1 ABC transporter ATP-binding protein
1(1)    WP_243544991.1 WP_243544991.1 type I secretion system permease/ATPase
1(1)    WP_388052166.1 WP_388052166.1 glycosyltransferase
1(1)    WP_118229992.1 WP_118229992.1 methionine ABC transporter ATP-binding protein
1(1)    WP_020591928.1 WP_020591928.1 type I secretion system permease/ATPase
1(1)    WP_085882207.1 WP_085882207.1 ABC transporter ATP-binding protein
1(1)    WP_005027453.1 WP_005027453.1 glycosyltransferase
1(1)    WP_227010806.1 WP_227010806.1 type I secretion system permease/ATPase
1(1)    WP_232088744.1 WP_232088744.1 MULTISPECIES: glycosyltransferase
1(1)    WP_407681916.1 WP_407681916.1 type I secretion system permease/ATPase
1(1)    WP_006000680.1 WP_006000680.1 type I secretion system permease/ATPase
1(1)    WP_015751019.1 WP_015751019.1 type I secretion system permease/ATPase
1(1)    WP_223294589.1 WP_223294589.1 type I secretion system permease/ATPase
1(1)    WP_022729643.1 WP_022729643.1 glycosyltransferase
1(1)    WP_025899684.1 WP_025899684.1 type I secretion system permease/ATPase
1(1)    WP_015336745.1 WP_015336745.1 ATP-binding cassette domain-containing protein
1(1)    WP_231117109.1 WP_231117109.1 type I secretion system permease/ATPase
1(1)    WP_013513836.1 WP_013513836.1 MULTISPECIES: ABC transporter ATP-binding protein
1(1)    WP_404823758.1 WP_404823758.1 type I secretion system permease/ATPase


2(1)    WP_154510633.1 WP_154510633.1 response regulator
2(1)    WP_232088708.1 WP_232088708.1 MULTISPECIES: sigma-54 interaction domain-containing protein
2(1)    WP_371384797.1 WP_371384797.1 sigma 54-interacting transcriptional regulator
2(1)    WP_097012986.1 WP_097012986.1 response regulator
2(1)    WP_013513852.1 WP_013513852.1 MULTISPECIES: HAMP domain-containing sensor histidine kinase
2(1)    WP_062252235.1 WP_062252235.1 sigma-54 interaction domain-containing protein
2(1)    WP_020000212.1 WP_020000212.1 response regulator transcription factor
2(1)    WP_245628638.1 WP_245628638.1 helix-turn-helix domain-containing protein
2(1)    WP_176290236.1 WP_176290236.1 chemotaxis protein
2(1)    WP_158947422.1 WP_158947422.1 response regulator
2(1)    WP_246387912.1 WP_246387912.1 sigma-54-dependent Fis family transcriptional regulator
2(1)    WP_154508515.1 WP_154508515.1 EAL domain-containing protein
2(1)    WP_118230619.1 WP_118230619.1 sigma-54 interaction domain-containing protein
2(1)    WP_229595194.1 WP_229595194.1 sigma 54-interacting transcriptional regulator
2(1)    WP_169560249.1 WP_169560249.1 HD domain-containing phosphohydrolase
2(1)    WP_092162513.1 WP_092162513.1 DVU0259 family response regulator domain-containing protein
2(1)    WP_062252919.1 WP_062252919.1 ATP-binding protein
2(1)    WP_353618294.1 WP_353618294.1 ATP-binding protein
2(1)    WP_015751023.1 WP_015751023.1 sigma-54 dependent transcriptional regulator
2(1)    WP_054649403.1 WP_054649403.1 ATP-binding protein
2(1)    WP_054649395.1 WP_054649395.1 response regulator
2(1)    WP_015416026.1 WP_015416026.1 sigma 54-interacting transcriptional regulator
2(1)    WP_071544370.1 WP_071544370.1 sigma 54-interacting transcriptional regulator
2(1)    WP_158947424.1 WP_158947424.1 sigma 54-interacting transcriptional regulator
2(1)    WP_051257977.1 WP_051257977.1 ATP-binding protein
2(1)    WP_066802131.1 WP_066802131.1 sigma 54-interacting transcriptional regulator
2(1)    WP_014321922.1 WP_014321922.1 sigma-54-dependent Fis family transcriptional regulator
2(1)    WP_279137085.1 WP_279137085.1 HD domain-containing phosphohydrolase
2(1)    WP_054649401.1 WP_054649401.1 sigma-54 dependent transcriptional regulator
```

2(1) WP_183717068.1 WP_183717068.1 EAL domain-containing protein
2(1) WP_326833658.1 WP_326833658.1 sensor domain-containing diguanylate cyclase
2(1) WP_251935999.1 WP_251935999.1 DNA-binding response regulator
2(1) WP_161315805.1 WP_161315805.1 hypothetical protein
2(1) WP_229772440.1 WP_229772440.1 HD-GYP domain-containing protein
2(1) WP_154508513.1 WP_154508513.1 HD domain-containing phosphohydrolase
2(1) WP_159104376.1 WP_159104376.1 HD-GYP domain-containing protein
2(1) WP_097012988.1 WP_097012988.1 sigma 54-interacting transcriptional regulator
2(1) WP_273522482.1 WP_273522482.1 GGDEF domain-containing protein
2(1) WP_022659518.1 WP_022659518.1 putative bifunctional diguanylate cyclase/phosphodiesterase
2(1) WP_066853453.1 WP_066853453.1 response regulator
2(1) WP_221277862.1 WP_221277862.1 sigma-54 interaction domain-containing protein
2(1) WP_054649397.1 WP_054649397.1 response regulator
2(1) WP_025899688.1 WP_025899688.1 PAS domain-containing protein
2(1) WP_245628631.1 WP_245628631.1 sensor histidine kinase
2(1) WP_251935997.1 WP_251935997.1 HD-GYP domain-containing protein
2(1) WP_074216492.1 WP_074216492.1 response regulator
2(1) WP_062252906.1 WP_062252906.1 EAL domain-containing protein
2(1) WP_158947421.1 WP_158947421.1 response regulator
2(1) WP_074216491.1 WP_074216491.1 HAMP domain-containing sensor histidine kinase
2(1) WP_202526243.1 WP_202526243.1 HD domain-containing phosphohydrolase
2(1) WP_062252889.1 WP_062252889.1 sensor domain-containing diguanylate cyclase
2(1) WP_169543092.1 WP_169543092.1 HD domain-containing phosphohydrolase
2(1) WP_066853455.1 WP_066853455.1 HAMP domain-containing sensor histidine kinase
2(1) WP_154510635.1 WP_154510635.1 PAS domain-containing sensor histidine kinase
2(1) WP_161338418.1 WP_161338418.1 DNA-binding response regulator
2(1) WP_012624275.1 WP_012624275.1 EAL domain-containing protein
2(1) WP_077072035.1 WP_077072035.1 HD domain-containing phosphohydrolase
2(1) WP_054649417.1 WP_054649417.1 ATP-binding protein
2(1) WP_273522485.1 WP_273522485.1 hybrid sensor histidine kinase/response regulator
2(1) WP_169543091.1 WP_169543091.1 DNA-binding response regulator
2(1) WP_169570189.1 WP_169570189.1 HD domain-containing phosphohydrolase
2(1) WP_161949027.1 WP_161949027.1 MULTISPECIES: GGDEF domain-containing protein
2(1) WP_018125282.1 WP_018125282.1 EAL domain-containing response regulator
2(1) WP_169570188.1 WP_169570188.1 DNA-binding response regulator
2(1) WP_265826338.1 WP_265826338.1 HD-GYP domain-containing protein
2(1) WP_051307242.1 WP_051307242.1 GGDEF domain-containing protein
2(1) WP_273522487.1 WP_273522487.1 hybrid sensor histidine kinase/response regulator
2(1) WP_207045709.1 WP_207045709.1 HD domain-containing phosphohydrolase
2(1) WP_207045711.1 WP_207045711.1 DNA-binding response regulator
2(1) WP_025899690.1 WP_025899690.1 DNA-binding response regulator
2(1) WP_062252904.1 WP_062252904.1 HD domain-containing phosphohydrolase
2(1) WP_279521172.1 WP_279521172.1 sigma 54-interacting transcriptional regulator
2(1) WP_158947420.1 WP_158947420.1 response regulator
2(1) WP_169560250.1 WP_169560250.1 response regulator
2(1) WP_223299899.1 WP_223299899.1 HD-GYP domain-containing protein
2(1) WP_245628630.1 WP_245628630.1 sensor histidine kinase
2(1) WP_085882220.1 WP_085882220.1 HD domain-containing phosphohydrolase
2(1) WP_158947419.1 WP_158947419.1 sensor histidine kinase
2(1) WP_338667782.1 WP_338667782.1 sigma 54-interacting transcriptional regulator
2(1) WP_288230542.1 WP_288230542.1 ATP-binding protein
2(1) WP_161338417.1 WP_161338417.1 HD-GYP domain-containing protein
2(1) WP_264983485.1 WP_264983485.1 sigma 54-interacting transcriptional regulator
2(1) WP_281762973.1 WP_281762973.1 response regulator
2(1) WP_020000213.1 WP_020000213.1 HAMP domain-containing sensor histidine kinase
2(1) WP_085882221.1 WP_085882221.1 helix-turn-helix transcriptional regulator
2(1) WP_177193266.1 WP_177193266.1 GGDEF domain-containing protein

3(1) WP_066802140.1 WP_066802140.1 transglutaminase-like cysteine peptidase
3(1) WP_156176855.1 WP_156176855.1 transglutaminase-like cysteine peptidase
3(1) WP_013513847.1 WP_013513847.1 transglutaminase-like cysteine peptidase
3(1) WP_231038959.1 WP_231038959.1 transglutaminase-like cysteine peptidase
3(1) WP_084264872.1 WP_084264872.1 transglutaminase-like cysteine peptidase
3(1) WP_018125290.1 WP_018125290.1 transglutaminase-like cysteine peptidase
3(1) WP_264983481.1 WP_264983481.1 transglutaminase-like cysteine peptidase
3(1) WP_245590754.1 WP_245590754.1 transglutaminase-like cysteine peptidase
3(1) WP_176244911.1 WP_176244911.1 transglutaminase-like cysteine peptidase
3(1) WP_236031536.1 WP_236031536.1 transglutaminase-like cysteine peptidase
3(1) WP_092379318.1 WP_092379318.1 transglutaminase-like cysteine peptidase
3(1) WP_246118364.1 WP_246118364.1 transglutaminase-like cysteine peptidase

3(1) WP_006000675.1 WP_006000675.1 transglutaminase-like cysteine peptidase
3(1) WP_155934525.1 WP_155934525.1 transglutaminase-like cysteine peptidase
3(1) WP_169726987.1 WP_169726987.1 transglutaminase-like cysteine peptidase
3(1) WP_154510627.1 WP_154510627.1 transglutaminase-like cysteine peptidase
3(1) WP_193371556.1 WP_193371556.1 transglutaminase-like cysteine peptidase
3(1) WP_169543093.1 WP_169543093.1 transglutaminase-like cysteine peptidase
3(1) WP_085901370.1 WP_085901370.1 transglutaminase-like cysteine peptidase
3(1) WP_174408948.1 WP_174408948.1 transglutaminase-like cysteine peptidase
3(1) WP_223294588.1 WP_223294588.1 transglutaminase-like cysteine peptidase
3(1) WP_012805218.1 WP_012805218.1 transglutaminase-like cysteine peptidase
3(1) WP_161949025.1 WP_161949025.1 transglutaminase-like cysteine peptidase
3(1) WP_236884816.1 WP_236884816.1 transglutaminase-like cysteine peptidase
3(1) WP_281762967.1 WP_281762967.1 transglutaminase-like cysteine peptidase
3(1) WP_005027457.1 WP_005027457.1 transglutaminase-like cysteine peptidase
3(1) WP_097012996.1 WP_097012996.1 transglutaminase-like cysteine peptidase
3(1) WP_174406921.1 WP_174406921.1 transglutaminase-like cysteine peptidase
3(1) WP_183719708.1 WP_183719708.1 transglutaminase-like cysteine peptidase
3(1) WP_147818644.1 WP_147818644.1 transglutaminase-like cysteine peptidase
3(1) WP_077072032.1 WP_077072032.1 transglutaminase-like cysteine peptidase
3(1) WP_245628634.1 WP_245628634.1 transglutaminase-like cysteine peptidase
3(1) WP_015751021.1 WP_015751021.1 transglutaminase-like cysteine peptidase
3(1) WP_251935991.1 WP_251935991.1 transglutaminase-like cysteine peptidase
3(1) WP_223299999.1 WP_223299999.1 transglutaminase-like cysteine peptidase
3(1) WP_169570193.1 WP_169570193.1 transglutaminase-like cysteine peptidase
3(1) WP_025899687.1 WP_025899687.1 transglutaminase-like cysteine peptidase
3(1) WP_161315810.1 WP_161315810.1 transglutaminase-like cysteine peptidase
3(1) WP_161338412.1 WP_161338412.1 transglutaminase-like cysteine peptidase
3(1) WP_229595160.1 WP_229595160.1 transglutaminase-like cysteine peptidase
3(1) WP_338667776.1 WP_338667776.1 transglutaminase-like cysteine peptidase
3(1) WP_285907339.1 WP_285907339.1 transglutaminase-like cysteine peptidase
3(1) WP_380255403.1 WP_380255403.1 transglutaminase-like cysteine peptidase
3(1) WP_148266956.1 WP_148266956.1 transglutaminase-like cysteine peptidase
3(1) WP_074216486.1 WP_074216486.1 transglutaminase-like cysteine peptidase
3(1) WP_161624767.1 WP_161624767.1 transglutaminase-like cysteine peptidase
3(1) WP_231895534.1 WP_231895534.1 transglutaminase-like cysteine peptidase
3(1) WP_072697218.1 WP_072697218.1 transglutaminase-like cysteine peptidase
3(1) WP_169560244.1 WP_169560244.1 transglutaminase-like cysteine peptidase
3(1) WP_242012421.1 WP_242012421.1 transglutaminase-like cysteine peptidase
3(1) WP_251935996.1 WP_251935996.1 transglutaminase-like cysteine peptidase
3(1) WP_085882215.1 WP_085882215.1 transglutaminase-like cysteine peptidase
3(1) WP_161338416.1 WP_161338416.1 transglutaminase-like cysteine peptidase
3(1) WP_169543097.1 WP_169543097.1 transglutaminase-like cysteine peptidase
3(1) WP_051307240.1 WP_051307240.1 transglutaminase-like cysteine peptidase
3(1) WP_071544367.1 WP_071544367.1 transglutaminase-like cysteine peptidase
3(1) WP_015416032.1 WP_015416032.1 transglutaminase-like cysteine peptidase
3(1) WP_161315807.1 WP_161315807.1 transglutaminase-like cysteine peptidase
3(1) WP_167230619.1 WP_167230619.1 transglutaminase-like cysteine peptidase
3(1) WP_081649842.1 WP_081649842.1 transglutaminase-like cysteine peptidase
3(1) WP_243838184.1 WP_243838184.1 transglutaminase-like cysteine peptidase, partial
3(1) WP_142895221.1 WP_142895221.1 transglutaminase-like cysteine peptidase
3(1) WP_243544993.1 WP_243544993.1 transglutaminase-like cysteine peptidase
3(1) WP_169560248.1 WP_169560248.1 transglutaminase-like cysteine peptidase
3(1) WP_265826327.1 WP_265826327.1 transglutaminase-like cysteine peptidase
3(1) WP_245622974.1 WP_245622974.1 transglutaminase-like cysteine peptidase
3(1) WP_371384792.1 WP_371384792.1 transglutaminase-like cysteine peptidase
3(1) WP_279521168.1 WP_279521168.1 transglutaminase-like cysteine peptidase
3(1) WP_162175094.1 WP_162175094.1 transglutaminase-like cysteine peptidase
3(1) WP_207045699.1 WP_207045699.1 transglutaminase-like cysteine peptidase
3(1) WP_020591926.1 WP_020591926.1 transglutaminase-like cysteine peptidase
3(1) WP_169570190.1 WP_169570190.1 transglutaminase-like cysteine peptidase
3(1) WP_193370322.1 WP_193370322.1 transglutaminase-like cysteine peptidase
3(1) WP_245170854.1 WP_245170854.1 transglutaminase-like cysteine peptidase
3(1) WP_407844443.1 WP_407844443.1 transglutaminase-like cysteine peptidase
3(1) WP_388050255.1 WP_388050255.1 transglutaminase-like cysteine peptidase
3(1) WP_234703607.1 WP_234703607.1 transglutaminase-like cysteine peptidase
3(1) WP_207045707.1 WP_207045707.1 transglutaminase-like cysteine peptidase
3(1) WP_092162492.1 WP_092162492.1 transglutaminase-like cysteine peptidase

4(1) WP_264983482.1 WP_264983482.1 HlyD family type I secretion periplasmic adaptor subunit
4(1) WP_367614074.1 WP_367614074.1 HlyD family type I secretion periplasmic adaptor subunit

4(1) WP_020000217.1 WP_020000217.1 HlyD family type I secretion periplasmic adaptor subunit
4(1) WP_092189414.1 WP_092189414.1 MULTISPECIES: HlyD family type I secretion periplasmic adaptor subunit
4(1) WP_066603226.1 WP_066603226.1 HlyD family type I secretion periplasmic adaptor subunit
4(1) WP_169570192.1 WP_169570192.1 HlyD family type I secretion periplasmic adaptor subunit
4(1) WP_041724680.1 WP_041724680.1 HlyD family type I secretion periplasmic adaptor subunit
4(1) WP_018125289.1 WP_018125289.1 HlyD family type I secretion periplasmic adaptor subunit
4(1) WP_074216487.1 WP_074216487.1 HlyD family type I secretion periplasmic adaptor subunit
4(1) WP_092162494.1 WP_092162494.1 HlyD family type I secretion periplasmic adaptor subunit
4(1) WP_083808688.1 WP_083808688.1 MULTISPECIES: HlyD family type I secretion periplasmic adaptor subunit
4(1) WP_169726986.1 WP_169726986.1 HlyD family type I secretion periplasmic adaptor subunit
4(1) WP_051384586.1 WP_051384586.1 HlyD family type I secretion periplasmic adaptor subunit
4(1) WP_014321918.1 WP_014321918.1 HlyD family type I secretion periplasmic adaptor subunit
4(1) WP_242621196.1 WP_242621196.1 HlyD family type I secretion periplasmic adaptor subunit
4(1) WP_207045701.1 WP_207045701.1 HlyD family type I secretion periplasmic adaptor subunit
4(1) WP_169560245.1 WP_169560245.1 HlyD family type I secretion periplasmic adaptor subunit
4(1) WP_085882216.1 WP_085882216.1 HlyD family type I secretion periplasmic adaptor subunit
4(1) WP_371384793.1 WP_371384793.1 HlyD family type I secretion periplasmic adaptor subunit
4(1) WP_097012995.1 WP_097012995.1 HlyD family type I secretion periplasmic adaptor subunit
4(1) WP_193370313.1 WP_193370313.1 efflux RND transporter periplasmic adaptor subunit
4(1) WP_108702587.1 WP_108702587.1 HlyD family type I secretion periplasmic adaptor subunit
4(1) WP_054649407.1 WP_054649407.1 HlyD family type I secretion periplasmic adaptor subunit
4(1) WP_092379233.1 WP_092379233.1 HlyD family type I secretion periplasmic adaptor subunit
4(1) WP_072697217.1 WP_072697217.1 HlyD family type I secretion periplasmic adaptor subunit
4(1) WP_404823705.1 WP_404823705.1 HlyD family type I secretion periplasmic adaptor subunit
4(1) WP_265826328.1 WP_265826328.1 HlyD family type I secretion periplasmic adaptor subunit
4(1) WP_246387910.1 WP_246387910.1 HlyD family type I secretion periplasmic adaptor subunit
4(1) WP_338667777.1 WP_338667777.1 HlyD family type I secretion periplasmic adaptor subunit
4(1) WP_150116148.1 WP_150116148.1 HlyD family type I secretion periplasmic adaptor subunit
4(1) WP_015416035.1 WP_015416035.1 efflux RND transporter periplasmic adaptor subunit
4(1) WP_380255401.1 WP_380255401.1 HlyD family type I secretion periplasmic adaptor subunit
4(1) WP_005027459.1 WP_005027459.1 HlyD family type I secretion periplasmic adaptor subunit
4(1) WP_158947429.1 WP_158947429.1 efflux RND transporter periplasmic adaptor subunit
4(1) WP_382421353.1 WP_382421353.1 HlyD family type I secretion periplasmic adaptor subunit
4(1) WP_015336746.1 WP_015336746.1 HlyD family type I secretion periplasmic adaptor subunit
4(1) WP_425607927.1 WP_425607927.1 HlyD family type I secretion periplasmic adaptor subunit
4(1) WP_193371555.1 WP_193371555.1 HlyD family type I secretion periplasmic adaptor subunit
4(1) WP_167230616.1 WP_167230616.1 HlyD family type I secretion periplasmic adaptor subunit
4(1) WP_092163010.1 WP_092163010.1 HlyD family type I secretion periplasmic adaptor subunit
4(1) WP_161315809.1 WP_161315809.1 HlyD family type I secretion periplasmic adaptor subunit
4(1) WP_251935992.1 WP_251935992.1 HlyD family type I secretion periplasmic adaptor subunit
4(1) WP_147818646.1 WP_147818646.1 HlyD family type I secretion periplasmic adaptor subunit
4(1) WP_169543096.1 WP_169543096.1 HlyD family type I secretion periplasmic adaptor subunit
4(1) WP_027187800.1 WP_027187800.1 HlyD family type I secretion periplasmic adaptor subunit
4(1) WP_161338413.1 WP_161338413.1 HlyD family type I secretion periplasmic adaptor subunit
4(1) WP_281762968.1 WP_281762968.1 HlyD family type I secretion periplasmic adaptor subunit
4(1) WP_015751020.1 WP_015751020.1 HlyD family type I secretion periplasmic adaptor subunit
4(1) WP_174406922.1 WP_174406922.1 HlyD family type I secretion periplasmic adaptor subunit
4(1) WP_047765662.1 WP_047765662.1 HlyD family type I secretion periplasmic adaptor subunit
4(1) WP_097013786.1 WP_097013786.1 efflux RND transporter periplasmic adaptor subunit
4(1) WP_085901371.1 WP_085901371.1 HlyD family type I secretion periplasmic adaptor subunit
4(1) WP_229595162.1 WP_229595162.1 HlyD family type I secretion periplasmic adaptor subunit
4(1) WP_273522477.1 WP_273522477.1 HlyD family type I secretion periplasmic adaptor subunit
4(1) WP_071544368.1 WP_071544368.1 HlyD family type I secretion periplasmic adaptor subunit
4(1) WP_010938317.1 WP_010938317.1 HlyD family type I secretion periplasmic adaptor subunit
4(1) WP_022729638.1 WP_022729638.1 HlyD family type I secretion periplasmic adaptor subunit
4(1) WP_174408949.1 WP_174408949.1 HlyD family type I secretion periplasmic adaptor subunit
4(1) WP_012805219.1 WP_012805219.1 HlyD family type I secretion periplasmic adaptor subunit
4(1) WP_317312916.1 WP_317312916.1 HlyD family type I secretion periplasmic adaptor subunit
4(1) WP_142895223.1 WP_142895223.1 HlyD family type I secretion periplasmic adaptor subunit
4(1) WP_051307239.1 WP_051307239.1 HlyD family type I secretion periplasmic adaptor subunit
4(1) WP_072697229.1 WP_072697229.1 biotin/lipoyl-binding protein
4(1) WP_066802139.1 WP_066802139.1 HlyD family type I secretion periplasmic adaptor subunit
4(1) WP_066853460.1 WP_066853460.1 HlyD family type I secretion periplasmic adaptor subunit
4(1) WP_199899656.1 WP_199899656.1 HlyD family type I secretion periplasmic adaptor subunit
4(1) WP_006000678.1 WP_006000678.1 HlyD family type I secretion periplasmic adaptor subunit
4(1) WP_158947426.1 WP_158947426.1 HlyD family type I secretion periplasmic adaptor subunit
4(1) WP_154508521.1 WP_154508521.1 HlyD family type I secretion periplasmic adaptor subunit
4(1) WP_404823706.1 WP_404823706.1 hypothetical protein
4(1) WP_243544992.1 WP_243544992.1 HlyD family type I secretion periplasmic adaptor subunit
4(1) WP_015416031.1 WP_015416031.1 HlyD family type I secretion periplasmic adaptor subunit
4(1) WP_046508115.1 WP_046508115.1 HlyD family type I secretion periplasmic adaptor subunit

4(1)	WP_020591927.1	WP_020591927.1	HlyD family type I secretion periplasmic adaptor subunit
4(1)	WP_011367388.1	WP_011367388.1	HlyD family type I secretion periplasmic adaptor subunit
4(1)	WP_026986122.1	WP_026986122.1	HlyD family type I secretion periplasmic adaptor subunit
4(1)	WP_066603253.1	WP_066603253.1	efflux RND transporter periplasmic adaptor subunit

5(1)	WP_011367386.1	WP_011367386.1	type I secretion C-terminal target domain-containing protein
5(1)	WP_281762963.1	WP_281762963.1	VCBS domain-containing protein
5(1)	WP_229595150.1	WP_229595150.1	VCBS domain-containing protein
5(1)	WP_066603213.1	WP_066603213.1	Ig-like domain-containing protein
5(1)	WP_028578377.1	WP_028578377.1	VCBS domain-containing protein
5(1)	WP_129586475.1	WP_129586475.1	calcium-binding protein
5(1)	WP_066603220.1	WP_066603220.1	hypothetical protein
5(1)	WP_205944923.1	WP_205944923.1	hypothetical protein
5(1)	WP_174406924.1	WP_174406924.1	cadherin-like domain-containing protein, partial
5(1)	WP_211226299.1	WP_211226299.1	nidogen-like domain-containing protein, partial
5(1)	WP_011792585.1	WP_011792585.1	DVU1012 family biofilm structural adhesin
5(1)	WP_264983477.1	WP_264983477.1	hypothetical protein
5(1)	WP_143077825.1	WP_143077825.1	hypothetical protein
5(1)	WP_174408951.1	WP_174408951.1	VCBS domain-containing protein, partial
5(1)	WP_198015331.1	WP_198015331.1	beta strand repeat-containing protein
5(1)	WP_229595152.1	WP_229595152.1	hypothetical protein
5(1)	WP_285907335.1	WP_285907335.1	tandem-95 repeat protein, partial
5(1)	WP_170830385.1	WP_170830385.1	tandem-95 repeat protein, partial
5(1)	WP_015751018.1	WP_015751018.1	beta strand repeat-containing protein
5(1)	WP_193371564.1	WP_193371564.1	hypothetical protein
5(1)	WP_092189402.1	WP_092189402.1	DUF5801 repeats-in-toxin domain-containing protein
5(1)	WP_084559377.1	WP_084559377.1	Calx-beta domain-containing protein
5(1)	WP_020591922.1	WP_020591922.1	DUF5801 repeats-in-toxin domain-containing protein, partial
5(1)	WP_066802144.1	WP_066802144.1	VCBS domain-containing protein
5(1)	WP_284690588.1	WP_284690588.1	Ig-like domain-containing protein
5(1)	WP_092189408.1	WP_092189408.1	hypothetical protein
5(1)	WP_066802143.1	WP_066802143.1	VCBS domain-containing protein
5(1)	WP_161949024.1	WP_161949024.1	DUF5801 repeats-in-toxin domain-containing protein
5(1)	WP_012805221.1	WP_012805221.1	DUF5801 repeats-in-toxin domain-containing protein
5(1)	WP_338667772.1	WP_338667772.1	calcium-binding protein
5(1)	WP_006000684.1	WP_006000684.1	cadherin-like domain-containing protein
5(1)	WP_193371565.1	WP_193371565.1	type I secretion C-terminal target domain-containing protein
5(1)	WP_071544364.1	WP_071544364.1	hypothetical protein
5(1)	WP_371384787.1	WP_371384787.1	calcium-binding protein
5(1)	WP_133987048.1	WP_133987048.1	hypothetical protein
5(1)	WP_371384788.1	WP_371384788.1	VCBS domain-containing protein
5(1)	WP_047765660.1	WP_047765660.1	S-layer family protein, partial
5(1)	WP_071544363.1	WP_071544363.1	VCBS domain-containing protein
5(1)	WP_012805223.1	WP_012805223.1	DUF5801 repeats-in-toxin domain-containing protein
5(1)	WP_229595154.1	WP_229595154.1	VCBS domain-containing protein
5(1)	WP_013513851.1	WP_013513851.1	Ig-like domain-containing protein
5(1)	WP_265826331.1	WP_265826331.1	cadherin domain-containing protein
5(1)	WP_264983476.1	WP_264983476.1	VCBS domain-containing protein
5(1)	WP_148266955.1	WP_148266955.1	hypothetical protein
5(1)	WP_143077826.1	WP_143077826.1	hypothetical protein
5(1)	WP_167230646.1	WP_167230646.1	calcium-binding protein
5(1)	WP_193370324.1	WP_193370324.1	FecR domain-containing protein
5(1)	WP_371384789.1	WP_371384789.1	hypothetical protein
5(1)	WP_338667773.1	WP_338667773.1	hypothetical protein
5(1)	WP_043810172.1	WP_043810172.1	hypothetical protein
5(1)	WP_279521165.1	WP_279521165.1	hypothetical protein
5(1)	WP_279388675.1	WP_279388675.1	cadherin-like domain-containing protein
5(1)	WP_281762964.1	WP_281762964.1	hypothetical protein
5(1)	WP_155934529.1	WP_155934529.1	tandem-95 repeat protein, partial
5(1)	WP_193371563.1	WP_193371563.1	type I secretion C-terminal target domain-containing protein
5(1)	WP_147818632.1	WP_147818632.1	Ig-like domain-containing protein
5(1)	WP_279521164.1	WP_279521164.1	Ig-like domain-containing protein
5(1)	WP_018125293.1	WP_018125293.1	tandem-95 repeat protein, partial
5(1)	WP_380255411.1	WP_380255411.1	beta strand repeat-containing protein, partial
5(1)	WP_147818636.1	WP_147818636.1	cadherin-like domain-containing protein
5(1)	WP_085901366.1	WP_085901366.1	DUF5801 repeats-in-toxin domain-containing protein
5(1)	WP_014321913.1	WP_014321913.1	VCBS domain-containing protein
5(1)	WP_028578376.1	WP_028578376.1	hypothetical protein
5(1)	WP_143077827.1	WP_143077827.1	DUF5801 repeats-in-toxin domain-containing protein
5(1)	WP_167230643.1	WP_167230643.1	S-layer family protein

```
5(1)    WP_012805224.1 WP_012805224.1 hypothetical protein


6(1)    WP_161338415.1 WP_161338415.1 TolC family outer membrane protein
6(1)    WP_020000215.1 WP_020000215.1 TolC family outer membrane protein
6(1)    WP_066603251.1 WP_066603251.1 efflux transporter outer membrane subunit
6(1)    WP_012805225.1 WP_012805225.1 TolC family outer membrane protein
6(1)    WP_430616112.1 WP_430616112.1 TolC family outer membrane protein
6(1)    WP_051608776.1 WP_051608776.1 TolC family outer membrane protein
6(1)    WP_015336742.1 WP_015336742.1 TolC family protein
6(1)    WP_013513850.1 WP_013513850.1 MULTISPECIES: TolC family outer membrane protein
6(1)    WP_022729636.1 WP_022729636.1 TolC family outer membrane protein
6(1)    WP_006000682.1 WP_006000682.1 TolC family outer membrane protein
6(1)    WP_074216489.1 WP_074216489.1 TolC family outer membrane protein
6(1)    WP_085882218.1 WP_085882218.1 TolC family outer membrane protein
6(1)    WP_092162490.1 WP_092162490.1 TolC family outer membrane protein
6(1)    WP_288230539.1 WP_288230539.1 TolC family outer membrane protein
6(1)    WP_062252912.1 WP_062252912.1 TolC family outer membrane protein
6(1)    WP_183717063.1 WP_183717063.1 TolC family outer membrane protein
6(1)    WP_394026236.1 WP_394026236.1 TolC family outer membrane protein
6(1)    WP_238528360.1 WP_238528360.1 TolC family outer membrane protein
6(1)    WP_071544365.1 WP_071544365.1 TolC family outer membrane protein
6(1)    WP_382421351.1 WP_382421351.1 TolC family outer membrane protein
6(1)    WP_161949023.1 WP_161949023.1 TolC family outer membrane protein
6(1)    WP_371384790.1 WP_371384790.1 TolC family outer membrane protein
6(1)    WP_338667774.1 WP_338667774.1 TolC family outer membrane protein
6(1)    WP_281762965.1 WP_281762965.1 TolC family outer membrane protein
6(1)    WP_066853456.1 WP_066853456.1 TolC family outer membrane protein
6(1)    WP_066603210.1 WP_066603210.1 TolC family outer membrane protein
6(1)    WP_040372704.1 WP_040372704.1 TolC family outer membrane protein
6(1)    WP_265826332.1 WP_265826332.1 TolC family outer membrane protein
6(1)    WP_169570191.1 WP_169570191.1 TolC family outer membrane protein
6(1)    WP_207045704.1 WP_207045704.1 TolC family outer membrane protein
6(1)    WP_022659515.1 WP_022659515.1 TolC family protein
6(1)    WP_016361060.1 WP_016361060.1 TolC family outer membrane protein
6(1)    WP_251935994.1 WP_251935994.1 TolC family outer membrane protein
6(1)    WP_169543094.1 WP_169543094.1 TolC family outer membrane protein
6(1)    WP_285907336.1 WP_285907336.1 TolC family outer membrane protein
6(1)    WP_279521166.1 WP_279521166.1 TolC family outer membrane protein
6(1)    WP_066807093.1 WP_066807093.1 TolC family outer membrane protein
6(1)    WP_243544989.1 WP_243544989.1 TolC family outer membrane protein
6(1)    WP_264983478.1 WP_264983478.1 TolC family outer membrane protein
6(1)    WP_246118365.1 WP_246118365.1 TolC family outer membrane protein
6(1)    WP_142895227.1 WP_142895227.1 TolC family outer membrane protein
6(1)    WP_147818640.1 WP_147818640.1 TolC family outer membrane protein
6(1)    WP_025899686.1 WP_025899686.1 TolC family outer membrane protein
6(1)    WP_167230614.1 WP_167230614.1 TolC family outer membrane protein
6(1)    WP_155934528.1 WP_155934528.1 TolC family outer membrane protein
6(1)    WP_010938312.1 WP_010938312.1 TolC family outer membrane protein
6(1)    WP_161315808.1 WP_161315808.1 TolC family outer membrane protein
6(1)    WP_027187798.1 WP_027187798.1 TolC family outer membrane protein
6(1)    WP_077072031.1 WP_077072031.1 TolC family outer membrane protein
6(1)    WP_169560247.1 WP_169560247.1 TolC family outer membrane protein
6(1)    WP_015751017.1 WP_015751017.1 TolC family outer membrane protein
6(1)    WP_193371554.1 WP_193371554.1 TolC family outer membrane protein
6(1)    WP_229595156.1 WP_229595156.1 TolC family outer membrane protein


7(1)    WP_012624791.1 WP_012624791.1 MULTISPECIES: PLP-dependent aminotransferase family protein
7(1)    WP_085882210.1 WP_085882210.1 pyridoxal phosphate-dependent aminotransferase
7(1)    WP_183719726.1 WP_183719726.1 PLP-dependent aminotransferase family protein
7(1)    WP_026986114.1 WP_026986114.1 PLP-dependent aminotransferase family protein
7(1)    WP_273522489.1 WP_273522489.1 pyridoxal phosphate-dependent aminotransferase
7(1)    WP_169570201.1 WP_169570201.1 threonine-phosphate decarboxylase CobD
7(1)    WP_161338403.1 WP_161338403.1 threonine-phosphate decarboxylase CobD
7(1)    WP_207045676.1 WP_207045676.1 threonine-phosphate decarboxylase CobD
7(1)    WP_084264868.1 WP_084264868.1 aminotransferase class I/II-fold pyridoxal phosphate-dependent enzyme
7(1)    WP_169560235.1 WP_169560235.1 threonine-phosphate decarboxylase CobD
7(1)    WP_022729628.1 WP_022729628.1 PLP-dependent aminotransferase family protein
7(1)    WP_084264870.1 WP_084264870.1 hypothetical protein
7(1)    WP_072697215.1 WP_072697215.1 pyridoxal phosphate-dependent aminotransferase
```

7(1) WP_097013008.1 WP_097013008.1 FadR/GntR family transcriptional regulator
7(1) WP_011792575.1 WP_011792575.1 histidinol-phosphate transaminase
7(1) WP_183719700.1 WP_183719700.1 LL-diaminopimelate aminotransferase
7(1) WP_183719704.1 WP_183719704.1 PLP-dependent aminotransferase family protein
7(1) WP_161315819.1 WP_161315819.1 threonine-phosphate decarboxylase CobD
7(1) WP_251935979.1 WP_251935979.1 threonine-phosphate decarboxylase CobD
7(1) WP_012624780.1 WP_012624780.1 LL-diaminopimelate aminotransferase
7(1) WP_158947439.1 WP_158947439.1 FadR/GntR family transcriptional regulator

8(1) WP_338667768.1 WP_338667768.1 nitroreductase family protein
8(1) WP_169570185.1 WP_169570185.1 NAD(P)H nitroreductase
8(1) WP_169544294.1 WP_169544294.1 nitroreductase
8(1) WP_066853468.1 WP_066853468.1 nitroreductase family protein
8(1) WP_371384783.1 WP_371384783.1 nitroreductase family protein
8(1) WP_041915951.1 WP_041915951.1 nitroreductase family protein
8(1) WP_161338421.1 WP_161338421.1 NAD(P)H nitroreductase
8(1) WP_025899693.1 WP_025899693.1 nitroreductase
8(1) WP_161315802.1 WP_161315802.1 nitroreductase family protein
8(1) WP_020000222.1 WP_020000222.1 nitroreductase family protein
8(1) WP_169560253.1 WP_169560253.1 NAD(P)H nitroreductase
8(1) WP_183719720.1 WP_183719720.1 nitroreductase family protein
8(1) WP_074216483.1 WP_074216483.1 nitroreductase family protein
8(1) WP_071544361.1 WP_071544361.1 nitroreductase family protein
8(1) WP_207045717.1 WP_207045717.1 nitroreductase family protein
8(1) WP_251936002.1 WP_251936002.1 nitroreductase family protein
8(1) WP_012624787.1 WP_012624787.1 MULTISPECIES: nitroreductase family protein
8(1) WP_066802150.1 WP_066802150.1 nitroreductase family protein

9(1) WP_169543100.1 WP_169543100.1 serine protease
9(1) WP_161315813.1 WP_161315813.1 trypsin-like serine peptidase
9(1) WP_169560241.1 WP_169560241.1 serine protease
9(1) WP_167230625.1 WP_167230625.1 serine protease
9(1) WP_142895219.1 WP_142895219.1 trypsin-like serine peptidase
9(1) WP_240969638.1 WP_240969638.1 trypsin-like serine peptidase
9(1) WP_053006126.1 WP_053006126.1 serine protease
9(1) WP_085901368.1 WP_085901368.1 trypsin-like serine peptidase
9(1) WP_251935988.1 WP_251935988.1 trypsin-like serine peptidase
9(1) WP_085901364.1 WP_085901364.1 trypsin-like serine peptidase
9(1) WP_161338409.1 WP_161338409.1 trypsin-like serine peptidase
9(1) WP_025899680.1 WP_025899680.1 serine protease
9(1) WP_020591924.1 WP_020591924.1 trypsin-like serine peptidase
9(1) WP_207045689.1 WP_207045689.1 trypsin-like serine peptidase
9(1) WP_046508117.1 WP_046508117.1 serine protease
9(1) WP_179953937.1 WP_179953937.1 trypsin-like serine peptidase
9(1) WP_193371558.1 WP_193371558.1 serine protease
9(1) WP_380255407.1 WP_380255407.1 trypsin-like serine peptidase

10(1) WP_279521175.1 WP_279521175.1 nitrogenase component 1
10(1) WP_338667787.1 WP_338667787.1 nitrogenase iron-molybdenum cofactor biosynthesis protein NifE
10(1) WP_264983490.1 WP_264983490.1 nitrogenase iron-molybdenum cofactor biosynthesis protein NifE
10(1) WP_014321927.1 WP_014321927.1 nitrogenase iron-molybdenum cofactor biosynthesis protein NifE
10(1) WP_371384799.1 WP_371384799.1 nitrogenase component 1
10(1) WP_071544374.1 WP_071544374.1 nitrogenase iron-molybdenum cofactor biosynthesis protein NifE
10(1) WP_066802127.1 WP_066802127.1 nitrogenase component 1
10(1) WP_183717084.1 WP_183717084.1 Fe-only nitrogenase subunit beta
10(1) WP_264983489.1 WP_264983489.1 nitrogenase component 1
10(1) WP_338667786.1 WP_338667786.1 nitrogenase component 1
10(1) WP_071544373.1 WP_071544373.1 nitrogenase component 1
10(1) WP_371384800.1 WP_371384800.1 nitrogenase iron-molybdenum cofactor biosynthesis protein NifE
10(1) WP_014321926.1 WP_014321926.1 nitrogenase component 1
10(1) WP_066802126.1 WP_066802126.1 nitrogenase iron-molybdenum cofactor biosynthesis protein NifE
10(1) WP_404823709.1 WP_404823709.1 nitrogenase iron-molybdenum cofactor biosynthesis protein NifE
10(1) WP_183717088.1 WP_183717088.1 nitrogenase iron-iron protein, alpha chain
10(1) WP_229595215.1 WP_229595215.1 nitrogenase component 1

11(1) WP_232005629.1 WP_232005629.1 zinc-dependent alcohol dehydrogenase family protein
11(1) WP_229595184.1 WP_229595184.1 zinc-dependent alcohol dehydrogenase family protein

11(1) WP_014321920.1 WP_014321920.1 zinc-dependent alcohol dehydrogenase family protein
11(1) WP_066802135.1 WP_066802135.1 zinc-dependent alcohol dehydrogenase family protein
11(1) WP_338667779.1 WP_338667779.1 zinc-dependent alcohol dehydrogenase family protein
11(1) WP_071544369.1 WP_071544369.1 zinc-dependent alcohol dehydrogenase family protein
11(1) WP_371384794.1 WP_371384794.1 zinc-dependent alcohol dehydrogenase family protein
11(1) WP_264983484.1 WP_264983484.1 zinc-binding dehydrogenase
11(1) WP_155934524.1 WP_155934524.1 zinc-dependent alcohol dehydrogenase family protein
11(1) WP_015416029.1 WP_015416029.1 zinc-binding dehydrogenase
11(1) WP_285907342.1 WP_285907342.1 zinc-dependent alcohol dehydrogenase family protein
11(1) WP_279521170.1 WP_279521170.1 zinc-dependent alcohol dehydrogenase family protein
11(1) WP_158947425.1 WP_158947425.1 zinc-dependent alcohol dehydrogenase family protein
11(1) WP_013513844.1 WP_013513844.1 MULTISPECIES: zinc-binding dehydrogenase

12(1) WP_066603206.1 WP_066603206.1 methyl-accepting chemotaxis protein
12(1) WP_020000209.1 WP_020000209.1 methyl-accepting chemotaxis protein
12(1) WP_062252895.1 WP_062252895.1 HAMP domain-containing methyl-accepting chemotaxis protein
12(1) WP_074216494.1 WP_074216494.1 methyl-accepting chemotaxis protein
12(1) WP_026364642.1 WP_026364642.1 methyl-accepting chemotaxis protein
12(1) WP_231038957.1 WP_231038957.1 methyl-accepting chemotaxis protein, partial
12(1) WP_066853449.1 WP_066853449.1 methyl-accepting chemotaxis protein
12(1) WP_209819256.1 WP_209819256.1 methyl-accepting chemotaxis protein
12(1) WP_035066074.1 WP_035066074.1 methyl-accepting chemotaxis protein
12(1) WP_066853447.1 WP_066853447.1 methyl-accepting chemotaxis protein
12(1) WP_209819255.1 WP_209819255.1 cache domain-containing protein
12(1) WP_167122726.1 WP_167122726.1 methyl-accepting chemotaxis protein

13(1) WP_319633636.1 WP_319633636.1 adenylate/guanylate cyclase domain-containing protein
13(1) WP_229595148.1 WP_229595148.1 CHASE2 domain-containing protein
13(1) WP_281762962.1 WP_281762962.1 CHASE2 domain-containing protein
13(1) WP_066802146.1 WP_066802146.1 CHASE2 domain-containing protein
13(1) WP_264983475.1 WP_264983475.1 CHASE2 domain-containing protein
13(1) WP_154508511.1 WP_154508511.1 CHASE2 domain-containing protein
13(1) WP_371384786.1 WP_371384786.1 CHASE2 domain-containing protein
13(1) WP_062252902.1 WP_062252902.1 CHASE2 domain-containing protein
13(1) WP_338667771.1 WP_338667771.1 CHASE2 domain-containing protein
13(1) WP_014321912.1 WP_014321912.1 CHASE2 domain-containing protein

14(1) WP_085901373.1 WP_085901373.1 c-type cytochrome
14(1) WP_020591931.1 WP_020591931.1 c-type cytochrome
14(1) WP_046508114.1 WP_046508114.1 cytochrome c family protein
14(1) WP_380255397.1 WP_380255397.1 c-type cytochrome
14(1) WP_338066533.1 WP_338066533.1 cytochrome c family protein
14(1) WP_245629509.1 WP_245629509.1 cytochrome c family protein
14(1) WP_020591929.1 WP_020591929.1 c-type cytochrome
14(1) WP_380255395.1 WP_380255395.1 c-type cytochrome
14(1) WP_047765664.1 WP_047765664.1 cytochrome c family protein

15(1) WP_338667780.1 WP_338667780.1 YgiQ family radical SAM protein
15(1) WP_229596924.1 WP_229596924.1 YgiQ family radical SAM protein
15(1) WP_371384795.1 WP_371384795.1 YgiQ family radical SAM protein
15(1) WP_371912882.1 WP_371912882.1 YgiQ family radical SAM protein
15(1) WP_014321921.1 WP_014321921.1 YgiQ family radical SAM protein
15(1) WP_281762970.1 WP_281762970.1 YgiQ family radical SAM protein
15(1) WP_407681917.1 WP_407681917.1 YgiQ family radical SAM protein
15(1) WP_404823707.1 WP_404823707.1 YgiQ family radical SAM protein
15(1) WP_066802133.1 WP_066802133.1 YgiQ family radical SAM protein

16(1) WP_169560237.1 WP_169560237.1 hypothetical protein
16(1) WP_207045681.1 WP_207045681.1 hypothetical protein
16(1) WP_169570199.1 WP_169570199.1 hypothetical protein
16(1) WP_161338405.1 WP_161338405.1 hypothetical protein
16(1) WP_025899677.1 WP_025899677.1 hypothetical protein
16(1) WP_161315817.1 WP_161315817.1 hypothetical protein
16(1) WP_251935982.1 WP_251935982.1 hypothetical protein
16(1) WP_169543104.1 WP_169543104.1 hypothetical protein

17(1)   WP_207045685.1 WP_207045685.1 DUF924 family protein
17(1)   WP_169543102.1 WP_169543102.1 DUF924 family protein
17(1)   WP_251935985.1 WP_251935985.1 DUF924 family protein
17(1)   WP_161315815.1 WP_161315815.1 DUF924 family protein
17(1)   WP_169560239.1 WP_169560239.1 DUF924 family protein
17(1)   WP_169570198.1 WP_169570198.1 DUF924 family protein
17(1)   WP_161338407.1 WP_161338407.1 DUF924 family protein
17(1)   WP_025899678.1 WP_025899678.1 DUF924 family protein

18(1)   WP_085882226.1 WP_085882226.1 cation:proton antiporter
18(1)   WP_161338406.1 WP_161338406.1 cation:proton antiporter
18(1)   WP_118230621.1 WP_118230621.1 cation:proton antiporter
18(1)   WP_167230592.1 WP_167230592.1 monovalent cation:proton antiporter-2 (CPA2) family protein
18(1)   WP_207045683.1 WP_207045683.1 cation:proton antiporter
18(1)   WP_251935987.1 WP_251935987.1 cation:proton antiporter
18(1)   WP_193371547.1 WP_193371547.1 monovalent cation:proton antiporter-2 (CPA2) family protein
18(1)   WP_161315816.1 WP_161315816.1 cation:proton antiporter

19(1)   WP_161338410.1 WP_161338410.1 hypothetical protein
19(1)   WP_251935989.1 WP_251935989.1 hypothetical protein
19(1)   WP_169560242.1 WP_169560242.1 hypothetical protein
19(1)   WP_207045691.1 WP_207045691.1 hypothetical protein
19(1)   WP_161315812.1 WP_161315812.1 hypothetical protein
19(1)   WP_169570195.1 WP_169570195.1 hypothetical protein
19(1)   WP_025899681.1 WP_025899681.1 hypothetical protein
19(1)   WP_169543099.1 WP_169543099.1 hypothetical protein

20(1)   WP_161338419.1 WP_161338419.1 pseudouridine synthase
20(1)   WP_181017831.1 WP_181017831.1 pseudouridine synthase
20(1)   WP_025899691.1 WP_025899691.1 pseudouridine synthase
20(1)   WP_169560251.1 WP_169560251.1 pseudouridine synthase
20(1)   WP_251936000.1 WP_251936000.1 pseudouridine synthase
20(1)   WP_161315804.1 WP_161315804.1 pseudouridine synthase
20(1)   WP_169570187.1 WP_169570187.1 pseudouridine synthase
20(1)   WP_207045713.1 WP_207045713.1 pseudouridine synthase

21(1)   WP_161315803.1 WP_161315803.1 universal stress protein
21(1)   WP_207045715.1 WP_207045715.1 universal stress protein
21(1)   WP_084264874.1 WP_084264874.1 universal stress protein
21(1)   WP_169560252.1 WP_169560252.1 universal stress protein
21(1)   WP_251936001.1 WP_251936001.1 universal stress protein
21(1)   WP_161338420.1 WP_161338420.1 universal stress protein
21(1)   WP_169543090.1 WP_169543090.1 universal stress protein
21(1)   WP_169570186.1 WP_169570186.1 universal stress protein

22(2)   WP_066802129.1 WP_066802129.1 MULTISPECIES: hypothetical protein
22(1)   WP_338667784.1 WP_338667784.1 hypothetical protein
22(1)   WP_242652854.1 WP_242652854.1 hypothetical protein
22(1)   WP_014321924.1 WP_014321924.1 hypothetical protein
22(1)   WP_264983486.1 WP_264983486.1 hypothetical protein
22(1)   WP_229595203.1 WP_229595203.1 hypothetical protein
22(1)   WP_279521173.1 WP_279521173.1 hypothetical protein

23(1)   WP_014321911.1 WP_014321911.1 M48 family metalloprotease
23(1)   WP_229595146.1 WP_229595146.1 M48 family metalloprotease
23(1)   WP_338667770.1 WP_338667770.1 M48 family metalloprotease
23(1)   WP_281762961.1 WP_281762961.1 M48 family metalloprotease
23(1)   WP_066802148.1 WP_066802148.1 M48 family metalloprotease
23(1)   WP_371384785.1 WP_371384785.1 M48 family metalloprotease
23(1)   WP_066603248.1 WP_066603248.1 M48 family metalloprotease
23(1)   WP_264983474.1 WP_264983474.1 M48 family metalloprotease

24(1)   WP_338667785.1 WP_338667785.1 radical SAM protein

24(1) WP_014321925.1 WP_014321925.1 radical SAM protein
24(1) WP_371384798.1 WP_371384798.1 radical SAM protein
24(1) WP_229595213.1 WP_229595213.1 radical SAM protein
24(1) WP_264983488.1 WP_264983488.1 NifB/NifX family molybdenum-iron cluster-binding protein
24(1) WP_279521174.1 WP_279521174.1 radical SAM protein
24(1) WP_066802128.1 WP_066802128.1 radical SAM protein
24(1) WP_071544372.1 WP_071544372.1 radical SAM protein

25(1) WP_169570205.1 WP_169570205.1 cob(I)yrinic acid a,c-diamide adenosyltransferase
25(1) WP_169560231.1 WP_169560231.1 cob(I)yrinic acid a,c-diamide adenosyltransferase
25(1) WP_161338400.1 WP_161338400.1 cob(I)yrinic acid a,c-diamide adenosyltransferase
25(1) WP_161315822.1 WP_161315822.1 cob(I)yrinic acid a,c-diamide adenosyltransferase
25(1) WP_207045670.1 WP_207045670.1 cob(I)yrinic acid a,c-diamide adenosyltransferase
25(1) WP_037493717.1 WP_037493717.1 cob(I)yrinic acid a,c-diamide adenosyltransferase
25(1) WP_251935976.1 WP_251935976.1 cob(I)yrinic acid a,c-diamide adenosyltransferase

26(1) WP_251935977.1 WP_251935977.1 cobaltochelatase subunit CobN
26(1) WP_207045672.1 WP_207045672.1 cobaltochelatase subunit CobN
26(1) WP_161338401.1 WP_161338401.1 cobaltochelatase subunit CobN
26(1) WP_025899673.1 WP_025899673.1 cobaltochelatase subunit CobN
26(1) WP_169570204.1 WP_169570204.1 cobaltochelatase subunit CobN
26(1) WP_161315821.1 WP_161315821.1 cobaltochelatase subunit CobN
26(1) WP_169560232.1 WP_169560232.1 cobaltochelatase subunit CobN

27(1) WP_161338402.1 WP_161338402.1 cobalamin biosynthesis protein CobW
27(1) WP_207045674.1 WP_207045674.1 cobalamin biosynthesis protein CobW
27(1) WP_161315820.1 WP_161315820.1 cobalamin biosynthesis protein CobW
27(1) WP_025899674.1 WP_025899674.1 cobalamin biosynthesis protein CobW
27(1) WP_169570203.1 WP_169570203.1 cobalamin biosynthesis protein CobW
27(1) WP_169560233.1 WP_169560233.1 cobalamin biosynthesis protein CobW
27(1) WP_251935978.1 WP_251935978.1 cobalamin biosynthesis protein CobW

28(1) WP_169543089.1 WP_169543089.1 OmpH family outer membrane protein
28(1) WP_207045729.1 WP_207045729.1 OmpH family outer membrane protein
28(1) WP_169570184.1 WP_169570184.1 OmpH family outer membrane protein
28(1) WP_169560254.1 WP_169560254.1 OmpH family outer membrane protein
28(1) WP_161315801.1 WP_161315801.1 OmpH family outer membrane protein
28(1) WP_251936004.1 WP_251936004.1 OmpH family outer membrane protein
28(1) WP_025899694.1 WP_025899694.1 OmpH family outer membrane protein

29(1) WP_066807094.1 WP_066807094.1 SH3 domain-containing protein
29(1) WP_281762960.1 WP_281762960.1 SH3 domain-containing protein
29(1) WP_371384784.1 WP_371384784.1 SH3 domain-containing protein
29(1) WP_014321910.1 WP_014321910.1 SH3 domain-containing protein
29(1) WP_229595144.1 WP_229595144.1 SH3 domain-containing protein
29(1) WP_264983473.1 WP_264983473.1 SH3 domain-containing protein
29(1) WP_338667769.1 WP_338667769.1 SH3 domain-containing protein

30(1) WP_046508119.1 WP_046508119.1 hypothetical protein
30(1) WP_085901367.1 WP_085901367.1 hypothetical protein
30(1) WP_193371559.1 WP_193371559.1 hypothetical protein
30(1) WP_047765661.1 WP_047765661.1 hypothetical protein
30(1) WP_020591923.1 WP_020591923.1 hypothetical protein
30(1) WP_380255409.1 WP_380255409.1 hypothetical protein
30(1) WP_167230628.1 WP_167230628.1 hypothetical protein

31(1) WP_193370328.1 WP_193370328.1 NAD-dependent epimerase/dehydratase family protein
31(1) WP_193370330.1 WP_193370330.1 NAD-dependent epimerase/dehydratase family protein
31(1) WP_193370329.1 WP_193370329.1 NAD-dependent epimerase/dehydratase family protein
31(1) WP_062252222.1 WP_062252222.1 NAD-dependent 4,6-dehydratase LegB
31(1) WP_154510607.1 WP_154510607.1 UDP-N-acetylglucosamine 4,6-dehydratase (inverting)
31(1) WP_083521993.1 WP_083521993.1 NAD-dependent epimerase/dehydratase family protein

32(1) WP_161315814.1 WP_161315814.1 SDR family NAD(P)-dependent oxidoreductase
32(1) WP_161338408.1 WP_161338408.1 SDR family NAD(P)-dependent oxidoreductase
32(1) WP_169543103.1 WP_169543103.1 SDR family NAD(P)-dependent oxidoreductase
32(1) WP_169560238.1 WP_169560238.1 SDR family NAD(P)-dependent oxidoreductase
32(1) WP_207045687.1 WP_207045687.1 SDR family NAD(P)-dependent oxidoreductase
32(1) WP_251935984.1 WP_251935984.1 SDR family NAD(P)-dependent oxidoreductase

33(1) WP_071544375.1 WP_071544375.1 GNAT family N-acetyltransferase
33(1) WP_014321928.1 WP_014321928.1 GNAT family N-acetyltransferase
33(1) WP_066802124.1 WP_066802124.1 GNAT family N-acetyltransferase
33(1) WP_371384801.1 WP_371384801.1 GNAT family N-acetyltransferase
33(1) WP_264983491.1 WP_264983491.1 GNAT family N-acetyltransferase
33(1) WP_338667788.1 WP_338667788.1 GNAT family N-acetyltransferase

34(1) WP_382421345.1 WP_382421345.1 TRAP transporter large permease
34(1) WP_015751014.1 WP_015751014.1 TRAP transporter large permease
34(1) WP_022729630.1 WP_022729630.1 TRAP transporter large permease
34(1) WP_006000688.1 WP_006000688.1 TRAP transporter large permease
34(1) WP_016361058.1 WP_016361058.1 TRAP transporter permease
34(1) WP_026986116.1 WP_026986116.1 TRAP transporter large permease

35(1) WP_167230608.1 WP_167230608.1 ribbon-helix-helix domain-containing protein
35(1) WP_142895229.1 WP_142895229.1 ribbon-helix-helix domain-containing protein
35(1) WP_193371552.1 WP_193371552.1 ribbon-helix-helix domain-containing protein
35(1) WP_382421356.1 WP_382421356.1 ribbon-helix-helix domain-containing protein
35(1) WP_022729640.1 WP_022729640.1 ribbon-helix-helix domain-containing protein
35(1) WP_081816360.1 WP_081816360.1 ribbon-helix-helix domain-containing protein

36(1) WP_388050267.1 WP_388050267.1 pyridoxal-phosphate dependent enzyme
36(1) WP_022729632.1 WP_022729632.1 hydroxyectoine utilization dehydratase EutB
36(1) WP_154508537.1 WP_154508537.1 threonine ammonia-lyase, biosynthetic
36(1) WP_245602279.1 WP_245602279.1 hydroxyectoine utilization dehydratase EutB
36(1) WP_388050264.1 WP_388050264.1 hypothetical protein
36(1) WP_018125286.1 WP_018125286.1 cysteine synthase A

37(1) WP_020000220.1 WP_020000220.1 class II fumarate hydratase
37(1) WP_005027436.1 WP_005027436.1 lyase family protein
37(1) WP_066853462.1 WP_066853462.1 aspartate ammonia-lyase
37(1) WP_005027437.1 WP_005027437.1 lyase family protein
37(1) WP_074216484.1 WP_074216484.1 class II fumarate hydratase

38(1) WP_011367399.1 WP_011367399.1 amino acid ABC transporter permease
38(1) WP_097012990.1 WP_097012990.1 amino acid ABC transporter permease
38(1) WP_118229991.1 WP_118229991.1 methionine ABC transporter permease
38(1) WP_011367400.1 WP_011367400.1 amino acid ABC transporter permease
38(1) WP_097012991.1 WP_097012991.1 amino acid ABC transporter permease

39(1) WP_026986125.1 WP_026986125.1 hypothetical protein
39(1) WP_142895228.1 WP_142895228.1 hypothetical protein
39(1) WP_167230611.1 WP_167230611.1 hypothetical protein
39(1) WP_193371553.1 WP_193371553.1 hypothetical protein
39(1) WP_022729641.1 WP_022729641.1 hypothetical protein

40(1) WP_193370317.1 WP_193370317.1 glycolate oxidase subunit GlcF
40(1) WP_074216495.1 WP_074216495.1 L-lactate dehydrogenase (quinone) large subunit LdhH
40(1) WP_020000208.1 WP_020000208.1 L-lactate dehydrogenase (quinone) large subunit LdhH
40(1) WP_066853445.1 WP_066853445.1 L-lactate dehydrogenase (quinone) large subunit LdhH
40(1) WP_209819258.1 WP_209819258.1 (Fe-S)-binding protein

41(1) WP_071544357.1 WP_071544357.1 LysR family transcriptional regulator
41(1) WP_147818664.1 WP_147818664.1 LysR family transcriptional regulator
41(1) WP_243545001.1 WP_243545001.1 LysR family transcriptional regulator

41(1)   WP_085882208.1 WP_085882208.1 LysR family transcriptional regulator
41(1)   WP_035066067.1 WP_035066067.1 LysR family transcriptional regulator

42(1)   WP_279137103.1 WP_279137103.1 NAD(P)-dependent oxidoreductase
42(1)   WP_265826334.1 WP_265826334.1 NAD(P)-dependent oxidoreductase
42(1)   WP_074216493.1 WP_074216493.1 NAD(P)-dependent oxidoreductase
42(1)   WP_020000211.1 WP_020000211.1 NAD(P)-dependent oxidoreductase
42(1)   WP_066853451.1 WP_066853451.1 NAD(P)-dependent oxidoreductase

43(1)   WP_288957099.1 WP_288957099.1 PEP/pyruvate-binding domain-containing protein
43(1)   WP_158947423.1 WP_158947423.1 PEP/pyruvate-binding domain-containing protein
43(1)   WP_281762972.1 WP_281762972.1 PEP/pyruvate-binding domain-containing protein
43(1)   WP_097012987.1 WP_097012987.1 PEP/pyruvate-binding domain-containing protein
43(1)   WP_154510631.1 WP_154510631.1 PEP/pyruvate-binding domain-containing protein

44(1)   WP_264983471.1 WP_264983471.1 site-specific integrase
44(1)   WP_338667767.1 WP_338667767.1 integrase
44(1)   WP_066802152.1 WP_066802152.1 integrase
44(1)   WP_281762958.1 WP_281762958.1 tyrosine-type recombinase/integrase
44(1)   WP_371384782.1 WP_371384782.1 tyrosine-type recombinase/integrase

45(1)   WP_204247788.1 WP_204247788.1 N-acetyltransferase
45(1)   WP_380255387.1 WP_380255387.1 GNAT family N-acetyltransferase
45(1)   WP_204248242.1 WP_204248242.1 N-acetyltransferase
45(1)   WP_198946718.1 WP_198946718.1 GNAT family N-acetyltransferase
45(1)   WP_047765669.1 WP_047765669.1 N-acetyltransferase

46(1)   WP_020591934.1 WP_020591934.1 glutamine--fructose-6-phosphate transaminase (isomerizing)
46(1)   WP_380255389.1 WP_380255389.1 glutamine--fructose-6-phosphate transaminase (isomerizing)
46(1)   WP_047765667.1 WP_047765667.1 glutamine--fructose-6-phosphate transaminase (isomerizing)
46(1)   WP_085901377.1 WP_085901377.1 glutamine--fructose-6-phosphate transaminase (isomerizing)
46(1)   WP_046508107.1 WP_046508107.1 glutamine--fructose-6-phosphate transaminase (isomerizing)

47(1)   WP_085901376.1 WP_085901376.1 bifunctional UDP-N-acetylglucosamine diphosphorylase/glucosamine-1-phosphate N-acetyltransferase GlmU
47(1)   WP_380255391.1 WP_380255391.1 bifunctional UDP-N-acetylglucosamine diphosphorylase/glucosamine-1-phosphate N-acetyltransferase GlmU
47(1)   WP_020591933.1 WP_020591933.1 bifunctional UDP-N-acetylglucosamine diphosphorylase/glucosamine-1-phosphate N-acetyltransferase GlmU
47(1)   WP_047765666.1 WP_047765666.1 bifunctional UDP-N-acetylglucosamine diphosphorylase/glucosamine-1-phosphate N-acetyltransferase GlmU
47(1)   WP_046508110.1 WP_046508110.1 bifunctional UDP-N-acetylglucosamine diphosphorylase/glucosamine-1-phosphate N-acetyltransferase GlmU

48(1)   WP_083921843.1 WP_083921843.1 HAD-IA family hydrolase
48(1)   WP_085901375.1 WP_085901375.1 HAD-IA family hydrolase
48(1)   WP_380255393.1 WP_380255393.1 HAD-IA family hydrolase
48(1)   WP_046508111.1 WP_046508111.1 HAD family hydrolase
48(1)   WP_047765665.1 WP_047765665.1 HAD family hydrolase

49(1)   WP_078063679.1 WP_078063679.1 helix-turn-helix domain-containing protein
49(1)   WP_264983470.1 WP_264983470.1 helix-turn-helix domain-containing protein
49(1)   WP_353618293.1 WP_353618293.1 helix-turn-helix domain-containing protein
49(1)   WP_422394377.1 WP_422394377.1 helix-turn-helix domain-containing protein
49(1)   WP_371384781.1 WP_371384781.1 helix-turn-helix domain-containing protein

50(1)   WP_012805216.1 WP_012805216.1 chromosomal replication initiator protein DnaA
50(1)   WP_028578380.1 WP_028578380.1 chromosomal replication initiator protein DnaA
50(1)   WP_092379238.1 WP_092379238.1 chromosomal replication initiator protein DnaA
50(1)   WP_092189420.1 WP_092189420.1 MULTISPECIES: chromosomal replication initiator protein DnaA

51(1) WP_097012999.1 WP_097012999.1 efflux RND transporter permease subunit
51(1) WP_072697226.1 WP_072697226.1 efflux RND transporter permease subunit
51(1) WP_015416034.1 WP_015416034.1 efflux RND transporter permease subunit
51(1) WP_158947428.1 WP_158947428.1 efflux RND transporter permease subunit

52(1) WP_169560255.1 WP_169560255.1 adenosine deaminase
52(1) WP_161315800.1 WP_161315800.1 adenosine deaminase
52(1) WP_169543088.1 WP_169543088.1 adenosine deaminase
52(1) WP_169570183.1 WP_169570183.1 adenosine deaminase

53(1) WP_193371561.1 WP_193371561.1 glycosyltransferase
53(1) WP_193371562.1 WP_193371562.1 glycosyltransferase
53(1) WP_167230637.1 WP_167230637.1 glycosyltransferase family 1 protein
53(1) WP_167230640.1 WP_167230640.1 glycosyltransferase family 1 protein

54(1) WP_243544987.1 WP_243544987.1 tetratricopeptide repeat protein
54(1) WP_118230614.1 WP_118230614.1 tetratricopeptide repeat protein
54(1) WP_243544988.1 WP_243544988.1 tetratricopeptide repeat protein
54(1) WP_072697210.1 WP_072697210.1 tetratricopeptide repeat protein

55(1) WP_043776192.1 WP_043776192.1 glycosyltransferase family protein
55(1) WP_092379256.1 WP_092379256.1 glycosyltransferase family protein
55(1) WP_245170856.1 WP_245170856.1 CgeB family protein
55(1) WP_161949026.1 WP_161949026.1 glycosyltransferase family protein

56(1) WP_207045668.1 WP_207045668.1 cobyric acid synthase
56(1) WP_161315823.1 WP_161315823.1 cobyric acid synthase
56(1) WP_251935975.1 WP_251935975.1 cobyric acid synthase
56(1) WP_161338399.1 WP_161338399.1 cobyric acid synthase

57(1) WP_027187803.1 WP_027187803.1 glutamate-1-semialdehyde 2,1-aminomutase
57(1) WP_062252232.1 WP_062252232.1 adenosylmethionine--8-amino-7-oxononanoate transaminase
57(1) WP_011367395.1 WP_011367395.1 aspartate aminotransferase family protein
57(1) WP_273522472.1 WP_273522472.1 glutamate-1-semialdehyde 2,1-aminomutase

58(1) WP_169570197.1 WP_169570197.1 hypothetical protein
58(1) WP_284347813.1 WP_284347813.1 hypothetical protein
58(1) WP_025899679.1 WP_025899679.1 hypothetical protein
58(1) WP_169543101.1 WP_169543101.1 hypothetical protein

59(1) WP_015416028.1 WP_015416028.1 GAK system CofD-like protein
59(1) WP_285907343.1 WP_285907343.1 GAK system CofD-like protein
59(1) WP_155934523.1 WP_155934523.1 GAK system CofD-like protein
59(1) WP_013513843.1 WP_013513843.1 MULTISPECIES: GAK system CofD-like protein

60(1) WP_285907346.1 WP_285907346.1 carboxynorspermidine decarboxylase
60(1) WP_367614072.1 WP_367614072.1 carboxynorspermidine decarboxylase
60(1) WP_013513840.1 WP_013513840.1 MULTISPECIES: carboxynorspermidine decarboxylase
60(1) WP_118230611.1 WP_118230611.1 carboxynorspermidine decarboxylase

61(1) WP_367614075.1 WP_367614075.1 MATE family efflux transporter
61(1) WP_242621180.1 WP_242621180.1 MATE family efflux transporter
61(1) WP_285907337.1 WP_285907337.1 MATE family efflux transporter
61(1) WP_013513849.1 WP_013513849.1 MULTISPECIES: MATE family efflux transporter

62(1) WP_264983492.1 WP_264983492.1 ferredoxin
62(1) WP_371384802.1 WP_371384802.1 ferredoxin
62(1) WP_066802122.1 WP_066802122.1 (2Fe-2S) ferredoxin domain-containing protein
62(1) WP_269943600.1 WP_269943600.1 ferredoxin

63(1) WP_155957878.1 WP_155957878.1 cytochrome P460 family protein
63(1) WP_388050248.1 WP_388050248.1 cytochrome P460 family protein
63(1) WP_388050250.1 WP_388050250.1 hypothetical protein
63(1) WP_155992129.1 WP_155992129.1 cytochrome P460 family protein

64(1) WP_022729633.1 WP_022729633.1 cyclodeaminase
64(1) WP_005027441.1 WP_005027441.1 ornithine cyclodeaminase family protein
64(1) WP_026986118.1 WP_026986118.1 cyclodeaminase
64(1) WP_388050261.1 WP_388050261.1 cyclodeaminase

65(1) WP_183719697.1 WP_183719697.1 glutamine synthetase III
65(1) WP_407844444.1 WP_407844444.1 glutamine synthetase III
65(1) WP_118230616.1 WP_118230616.1 glutamine synthetase III
65(1) WP_118229993.1 WP_118229993.1 glutamine synthetase III

66(1) WP_074216481.1 WP_074216481.1 3-dehydroquinate synthase II family protein
66(1) WP_020000225.1 WP_020000225.1 3-dehydroquinate synthase II family protein
66(1) WP_066853474.1 WP_066853474.1 3-dehydroquinate synthase II family protein

67(1) WP_066853472.1 WP_066853472.1 2-amino-3,7-dideoxy-D-threo-hept-6-ulosonate synthase
67(1) WP_020000224.1 WP_020000224.1 2-amino-3,7-dideoxy-D-threo-hept-6-ulosonate synthase
67(1) WP_074216482.1 WP_074216482.1 2-amino-3,7-dideoxy-D-threo-hept-6-ulosonate synthase

68(1) WP_074216496.1 WP_074216496.1 lactate utilization protein
68(1) WP_066853443.1 WP_066853443.1 lactate utilization protein
68(1) WP_026364641.1 WP_026364641.1 lactate utilization protein

69(1) WP_015336749.1 WP_015336749.1 L-lactate permease
69(1) WP_077072029.1 WP_077072029.1 L-lactate permease
69(1) WP_035066063.1 WP_035066063.1 L-lactate permease

70(1) WP_047765668.1 WP_047765668.1 NUDIX domain-containing protein
70(1) WP_085901378.1 WP_085901378.1 NUDIX domain-containing protein
70(1) WP_046508106.1 WP_046508106.1 NUDIX domain-containing protein

71(2) WP_092189424.1 WP_092189424.1 MULTISPECIES: Holliday junction branch migration DNA helicase RuvB
71(1) WP_028578382.1 WP_028578382.1 Holliday junction branch migration DNA helicase RuvB

72(1) WP_092379241.1 WP_092379241.1 FAD-dependent thymidylate synthase
72(1) WP_028578381.1 WP_028578381.1 FAD-dependent thymidylate synthase
72(1) WP_092189422.1 WP_092189422.1 MULTISPECIES: FAD-dependent thymidylate synthase

73(1) WP_092379244.1 WP_092379244.1 Holliday junction branch migration protein RuvA
73(1) WP_092189426.1 WP_092189426.1 MULTISPECIES: Holliday junction branch migration protein RuvA
73(1) WP_028578383.1 WP_028578383.1 Holliday junction branch migration protein RuvA

74(1) WP_028578384.1 WP_028578384.1 crossover junction endodeoxyribonuclease RuvC
74(1) WP_092189428.1 WP_092189428.1 MULTISPECIES: crossover junction endodeoxyribonuclease RuvC
74(1) WP_092379247.1 WP_092379247.1 crossover junction endodeoxyribonuclease RuvC

75(1) WP_028578385.1 WP_028578385.1 YebC/PmpR family DNA-binding transcriptional regulator
75(1) WP_092379250.1 WP_092379250.1 YebC/PmpR family DNA-binding transcriptional regulator
75(1) WP_092189430.1 WP_092189430.1 MULTISPECIES: YebC/PmpR family DNA-binding transcriptional regulator

76(1) WP_028578386.1 WP_028578386.1 RlmE family RNA methyltransferase
76(1) WP_092189432.1 WP_092189432.1 MULTISPECIES: RlmE family RNA methyltransferase
76(1) WP_092379253.1 WP_092379253.1 RlmE family RNA methyltransferase

```
77(1)   WP_092379264.1 WP_092379264.1 flavin reductase family protein
77(1)   WP_092189440.1 WP_092189440.1 MULTISPECIES: flavin reductase family protein
77(1)   WP_028578387.1 WP_028578387.1 flavin reductase family protein


78(1)   WP_072697220.1 WP_072697220.1 ATP-dependent zinc metalloprotease FtsH
78(1)   WP_062252893.1 WP_062252893.1 ATP-dependent zinc metalloprotease FtsH
78(1)   WP_154508507.1 WP_154508507.1 ATP-dependent zinc metalloprotease FtsH


79(1)   WP_015416036.1 WP_015416036.1 DUF1844 domain-containing protein
79(1)   WP_097013000.1 WP_097013000.1 DUF1844 domain-containing protein
79(1)   WP_158947430.1 WP_158947430.1 DUF1844 domain-containing protein


80(1)   WP_097013001.1 WP_097013001.1 N-acetyl-gamma-glutamyl-phosphate reductase
80(1)   WP_158947431.1 WP_158947431.1 N-acetyl-gamma-glutamyl-phosphate reductase
80(1)   WP_015416037.1 WP_015416037.1 N-acetyl-gamma-glutamyl-phosphate reductase


81(1)   WP_025899675.1 WP_025899675.1 VOC family protein
81(1)   WP_169570202.1 WP_169570202.1 VOC family protein
81(1)   WP_169560234.1 WP_169560234.1 VOC family protein


82(1)   WP_183717057.1 WP_183717057.1 phosphomethylpyrimidine synthase ThiC
82(1)   WP_022659507.1 WP_022659507.1 phosphomethylpyrimidine synthase ThiC
82(1)   WP_012624264.1 WP_012624264.1 MULTISPECIES: phosphomethylpyrimidine synthase ThiC


83(1)   WP_183717059.1 WP_183717059.1 potassium channel family protein
83(1)   WP_012624266.1 WP_012624266.1 TrkA family potassium uptake protein
83(1)   WP_022659509.1 WP_022659509.1 potassium channel family protein


84(1)   WP_183717072.1 WP_183717072.1 pyridoxal-phosphate-dependent aminotransferase family protein
84(1)   WP_012624277.1 WP_012624277.1 MULTISPECIES: alanine--glyoxylate aminotransferase family protein
84(1)   WP_022659520.1 WP_022659520.1 pyridoxal-phosphate-dependent aminotransferase family protein


85(1)   WP_183717869.1 WP_183717869.1 rubrerythrin
85(1)   WP_022659514.1 WP_022659514.1 rubrerythrin
85(1)   WP_012624269.1 WP_012624269.1 MULTISPECIES: rubrerythrin


86(1)   WP_118230617.1 WP_118230617.1 P-II family nitrogen regulator
86(1)   WP_183719695.1 WP_183719695.1 P-II family nitrogen regulator
86(1)   WP_012624777.1 WP_012624777.1 MULTISPECIES: P-II family nitrogen regulator


87(1)   WP_022659529.1 WP_022659529.1 pyridine nucleotide-disulfide oxidoreductase/dicluster-binding protein
87(1)   WP_147818650.1 WP_147818650.1 FAD-dependent oxidoreductase
87(1)   WP_183719718.1 WP_183719718.1 FAD-dependent oxidoreductase


88(1)   WP_193371546.1 WP_193371546.1 acetyl-CoA carboxylase biotin carboxylase subunit
88(1)   WP_167230588.1 WP_167230588.1 acetyl-CoA carboxylase biotin carboxylase subunit
88(1)   WP_014321908.1 WP_014321908.1 formate-dependent phosphoribosylglycinamide formyltransferase


89(1)   WP_142895230.1 WP_142895230.1 hypothetical protein
89(1)   WP_193371551.1 WP_193371551.1 hypothetical protein
89(1)   WP_167230605.1 WP_167230605.1 hypothetical protein


90(1)   WP_062252891.1 WP_062252891.1 dihydropteroate synthase
90(1)   WP_195840916.1 WP_195840916.1 dihydropteroate synthase
90(1)   WP_072697221.1 WP_072697221.1 dihydropteroate synthase
```

91(1)   WP_046508168.1 WP_046508168.1 shikimate kinase
91(1)   WP_204248243.1 WP_204248243.1 hypothetical protein
91(1)   WP_198946719.1 WP_198946719.1 shikimate kinase

92(1)   WP_193370319.1 WP_193370319.1 FAD-linked oxidase C-terminal domain-containing protein
92(1)   WP_209819257.1 WP_209819257.1 FAD-binding oxidoreductase
92(1)   WP_193370318.1 WP_193370318.1 glycolate oxidase subunit GlcE

93(1)   WP_034637254.1 WP_034637254.1 precorrin-3B C(17)-methyltransferase
93(1)   WP_219821550.1 WP_219821550.1 uroporphyrinogen-III C-methyltransferase
93(1)   WP_034637257.1 WP_034637257.1 precorrin-2 C(20)-methyltransferase

94(1)   WP_221277864.1 WP_221277864.1 bifunctional transcriptional activator/DNA repair enzyme AdaA
94(1)   WP_012624790.1 WP_012624790.1 MULTISPECIES: bifunctional transcriptional activator/DNA repair enzyme AdaA
94(1)   WP_183719712.1 WP_183719712.1 AraC family transcriptional regulator

95(1)   WP_236884814.1 WP_236884814.1 IS5 family transposase
95(1)   WP_236884815.1 WP_236884815.1 IS5 family transposase
95(1)   WP_211259002.1 WP_211259002.1 IS5 family transposase

96(1)   WP_243544999.1 WP_243544999.1 metallophosphoesterase
96(1)   WP_158947436.1 WP_158947436.1 metallophosphoesterase
96(1)   WP_097013006.1 WP_097013006.1 metallophosphoesterase

97(1)   WP_245590753.1 WP_245590753.1 arsenate reductase ArsC
97(1)   WP_231038958.1 WP_231038958.1 arsenate reductase ArsC
97(1)   WP_243838183.1 WP_243838183.1 arsenate reductase ArsC

98(1)   WP_022659508.1 WP_022659508.1 TrkH family potassium uptake protein
98(1)   WP_246387907.1 WP_246387907.1 TrkH family potassium uptake protein
98(1)   WP_041724677.1 WP_041724677.1 TrkH family potassium uptake protein

99(1)   WP_143142690.1 WP_143142690.1 MULTISPECIES: pyridoxamine 5'-phosphate oxidase family protein
99(1)   WP_183717080.1 WP_183717080.1 pyridoxamine 5'-phosphate oxidase family protein
99(1)   WP_246388082.1 WP_246388082.1 pyridoxamine 5'-phosphate oxidase family protein

100(1) WP_207045719.1 WP_207045719.1 glutathione S-transferase family protein
100(1) WP_251936003.1 WP_251936003.1 glutathione S-transferase family protein
100(1) WP_161338422.1 WP_161338422.1 glutathione S-transferase family protein

101(1) WP_174406911.1 WP_174406911.1 adenine deaminase
101(1) WP_265826316.1 WP_265826316.1 adenine deaminase
101(1) WP_174408939.1 WP_174408939.1 adenine deaminase

102(1) WP_265826317.1 WP_265826317.1 ABC transporter permease
102(1) WP_243451264.1 WP_243451264.1 ABC transporter permease
102(1) WP_174406912.1 WP_174406912.1 ABC transporter permease

103(1) WP_265826318.1 WP_265826318.1 ABC transporter permease
103(1) WP_174406913.1 WP_174406913.1 ABC transporter permease
103(1) WP_174408940.1 WP_174408940.1 ABC transporter permease

104(1) WP_174406915.1 WP_174406915.1 phosphoribosyltransferase family protein
104(1) WP_174408942.1 WP_174408942.1 phosphoribosyltransferase family protein
104(1) WP_265826321.1 WP_265826321.1 phosphoribosyltransferase family protein

105(1) WP_174406916.1 WP_174406916.1 BMP family ABC transporter substrate-binding protein
105(1) WP_265826322.1 WP_265826322.1 BMP family ABC transporter substrate-binding protein
105(1) WP_174408945.1 WP_174408945.1 BMP family ABC transporter substrate-binding protein

106(1) WP_174408946.1 WP_174408946.1 hypothetical protein
106(1) WP_174406917.1 WP_174406917.1 hypothetical protein
106(1) WP_265826323.1 WP_265826323.1 hypothetical protein

107(1) WP_273522463.1 WP_273522463.1 molecular chaperone DnaJ
107(1) WP_011792590.1 WP_011792590.1 DnaJ domain-containing protein
107(1) WP_130957890.1 WP_130957890.1 J domain-containing protein

108(1) WP_285907345.1 WP_285907345.1 saccharopine dehydrogenase family protein
108(1) WP_155934522.1 WP_155934522.1 saccharopine dehydrogenase family protein
108(1) WP_013513841.1 WP_013513841.1 MULTISPECIES: saccharopine dehydrogenase family protein

109(1) WP_285907347.1 WP_285907347.1 agmatinase
109(1) WP_155934518.1 WP_155934518.1 agmatinase
109(1) WP_013513839.1 WP_013513839.1 MULTISPECIES: agmatinase

110(1) WP_285907349.1 WP_285907349.1 SLC13 family permease
110(1) WP_097013007.1 WP_097013007.1 SLC13 family permease
110(1) WP_155934514.1 WP_155934514.1 DASS family sodium-coupled anion symporter

111(1) WP_155934512.1 WP_155934512.1 iron ABC transporter substrate-binding protein
111(1) WP_285907350.1 WP_285907350.1 iron ABC transporter substrate-binding protein
111(1) WP_013513838.1 WP_013513838.1 MULTISPECIES: iron ABC transporter substrate-binding protein

112(1) WP_223299998.1 WP_223299998.1 hypothetical protein
112(1) WP_245170855.1 WP_245170855.1 hypothetical protein
112(1) WP_288229161.1 WP_288229161.1 hypothetical protein

113(1) WP_229772574.1 WP_229772574.1 pseudaminic acid synthase
113(1) WP_288229170.1 WP_288229170.1 N-acetylneuraminate synthase family protein
113(1) WP_062252224.1 WP_062252224.1 N-acetylneuraminate synthase family protein

114(1) WP_296984990.1 WP_296984990.1 M48 family metallopeptidase
114(1) WP_246387908.1 WP_246387908.1 M48 family metallopeptidase
114(1) WP_022659512.1 WP_022659512.1 M48 family metallopeptidase

115(1) WP_338667783.1 WP_338667783.1 hypothetical protein
115(1) WP_014321923.1 WP_014321923.1 hypothetical protein
115(1) WP_278248037.1 WP_278248037.1 hypothetical protein

116(1) WP_367614073.1 WP_367614073.1 biosynthetic arginine decarboxylase
116(1) WP_013513842.1 WP_013513842.1 MULTISPECIES: biosynthetic arginine decarboxylase
116(1) WP_285907344.1 WP_285907344.1 biosynthetic arginine decarboxylase

117(1) WP_085901379.1 WP_085901379.1 endonuclease/exonuclease/phosphatase family protein
117(1) WP_380255385.1 WP_380255385.1 endonuclease/exonuclease/phosphatase family protein
117(1) WP_046508104.1 WP_046508104.1 endonuclease/exonuclease/phosphatase family protein

118(1) WP_026986115.1 WP_026986115.1 TRAP transporter small permease
118(1) WP_382421344.1 WP_382421344.1 TRAP transporter small permease
118(1) WP_245560780.1 WP_245560780.1 TRAP transporter small permease

119(1) WP_022729634.1 WP_022729634.1 ectoine hydrolase DoeA
119(1) WP_026986119.1 WP_026986119.1 ectoine hydrolase DoeA
119(1) WP_382421349.1 WP_382421349.1 ectoine hydrolase DoeA

120(1) WP_026986126.1 WP_026986126.1 lysylphosphatidylglycerol synthase transmembrane domain-containing protein
120(1) WP_211217849.1 WP_211217849.1 lysylphosphatidylglycerol synthase transmembrane domain-containing protein
120(1) WP_382421358.1 WP_382421358.1 YbhN family protein

121(1) WP_026986128.1 WP_026986128.1 DMT family transporter
121(1) WP_022729644.1 WP_022729644.1 DMT family transporter
121(1) WP_382421360.1 WP_382421360.1 DMT family transporter

122(1) WP_382421362.1 WP_382421362.1 Flp family type IVb pilin
122(1) WP_026989573.1 WP_026989573.1 Flp family type IVb pilin
122(1) WP_035723055.1 WP_035723055.1 Flp family type IVb pilin

123(1) WP_382421363.1 WP_382421363.1 pilus assembly protein TadG-related protein
123(1) WP_022729647.1 WP_022729647.1 TadE/TadG family type IV pilus assembly protein
123(1) WP_081816361.1 WP_081816361.1 pilus assembly protein

124(1) WP_382421364.1 WP_382421364.1 prepilin peptidase
124(1) WP_022729648.1 WP_022729648.1 prepilin peptidase
124(1) WP_051608778.1 WP_051608778.1 prepilin peptidase

125(1) WP_081649843.1 WP_081649843.1 Flp pilus assembly protein CpaB
125(1) WP_026986132.1 WP_026986132.1 Flp pilus assembly protein CpaB
125(1) WP_382421365.1 WP_382421365.1 Flp pilus assembly protein CpaB

126(1) WP_155957877.1 WP_155957877.1 Asp/Glu racemase
126(1) WP_155992127.1 WP_155992127.1 Asp/Glu racemase
126(1) WP_388050270.1 WP_388050270.1 hypothetical protein

127(1) WP_394026239.1 WP_394026239.1 FlxA-like family protein
127(1) WP_183717061.1 WP_183717061.1 FlxA-like family protein
127(1) WP_051135444.1 WP_051135444.1 FlxA-like family protein

128(1) WP_118230618.1 WP_118230618.1 ammonium transporter
128(1) WP_407844445.1 WP_407844445.1 ammonium transporter
128(1) WP_183719693.1 WP_183719693.1 ammonium transporter

129(1) WP_012805228.1 WP_012805228.1 FeoA family protein
129(1) WP_012624281.1 WP_012624281.1 MULTISPECIES: ferrous iron transport protein A

130(1) WP_018125279.1 WP_018125279.1 glycine betaine uptake BCCT transporter
130(1) WP_005027428.1 WP_005027428.1 BCCT family transporter

131(1) WP_015751031.1 WP_015751031.1 flagellar hook assembly protein FlgD
131(1) WP_022657431.1 WP_022657431.1 flagellar hook assembly protein FlgD

132(1) WP_005027471.1 WP_005027471.1 iron-containing alcohol dehydrogenase
132(1) WP_022659505.1 WP_022659505.1 iron-containing alcohol dehydrogenase

133(1) WP_035723052.1 WP_035723052.1 N(2)-acetyl-L-2,4-diaminobutanoate deacetylase DoeB
133(1) WP_022729635.1 WP_022729635.1 N(2)-acetyl-L-2,4-diaminobutanoate deacetylase DoeB

134(1) WP_062252237.1 WP_062252237.1 cupin domain-containing protein
134(1) WP_022659523.1 WP_022659523.1 cupin domain-containing protein

135(1) WP_020000221.1 WP_020000221.1 winged helix-turn-helix transcriptional regulator
135(1) WP_066853464.1 WP_066853464.1 helix-turn-helix domain-containing protein

136(1) WP_074216477.1 WP_074216477.1 aminodeoxychorismate/anthranilate synthase component II
136(1) WP_026364646.1 WP_026364646.1 anthranilate synthase component II

137(1) WP_074216478.1 WP_074216478.1 anthranilate synthase component I family protein
137(1) WP_026364645.1 WP_026364645.1 anthranilate synthase component I family protein

138(1) WP_074216479.1 WP_074216479.1 prephenate dehydrogenase
138(1) WP_020000228.1 WP_020000228.1 prephenate dehydrogenase/arogenate dehydrogenase family protein

139(1) WP_074216480.1 WP_074216480.1 3-phosphoshikimate 1-carboxyvinyltransferase
139(1) WP_020000227.1 WP_020000227.1 3-phosphoshikimate 1-carboxyvinyltransferase

140(1) WP_074216490.1 WP_074216490.1 hypothetical protein
140(1) WP_020000214.1 WP_020000214.1 hypothetical protein

141(1) WP_074216497.1 WP_074216497.1 DRTGG domain-containing protein
141(1) WP_066853441.1 WP_066853441.1 DRTGG domain-containing protein

142(1) WP_081816362.1 WP_081816362.1 type II and III secretion system protein family protein
142(1) WP_051152313.1 WP_051152313.1 type II and III secretion system protein family protein

143(1) WP_015336744.1 WP_015336744.1 hypothetical protein
143(1) WP_092163008.1 WP_092163008.1 hypothetical protein

144(1) WP_092189436.1 WP_092189436.1 glycosyltransferase family 2 protein
144(1) WP_092379258.1 WP_092379258.1 glycosyltransferase

145(1) WP_097012992.1 WP_097012992.1 GAK system XXXCH domain-containing protein
145(1) WP_015416027.1 WP_015416027.1 GAK system XXXCH domain-containing protein

146(1) WP_097012998.1 WP_097012998.1 sodium:proton antiporter
146(1) WP_054649405.1 WP_054649405.1 sodium:proton antiporter

147(1) WP_015751011.1 WP_015751011.1 ATP-dependent protease ATPase subunit HslU
147(1) WP_118229979.1 WP_118229979.1 ATP-dependent protease ATPase subunit HslU

148(1) WP_027187801.1 WP_027187801.1 thiol peroxidase
148(1) WP_118229980.1 WP_118229980.1 thiol peroxidase

149(1) WP_011367401.1 WP_011367401.1 50S ribosomal protein L11 methyltransferase
149(1) WP_118229982.1 WP_118229982.1 50S ribosomal protein L11 methyltransferase

150(1) WP_139296783.1 WP_139296783.1 prephenate dehydratase
150(1) WP_020000226.1 WP_020000226.1 prephenate dehydratase

151(1) WP_139839514.1 WP_139839514.1 TetR/AcrR family transcriptional regulator
151(1) WP_071544360.1 WP_071544360.1 TetR/AcrR family transcriptional regulator

152(1) WP_022657424.1 WP_022657424.1 long-chain-fatty-acid--CoA ligase
152(1) WP_142895225.1 WP_142895225.1 acyl-

153(1) WP_147818652.1 WP_147818652.1 RnfABCDGE type electron transport complex subunit A
153(1) WP_147818654.1 WP_147818654.1 electron transport complex subunit E

154(1) WP_062252898.1 WP_062252898.1 tetratricopeptide repeat protein
154(1) WP_154508509.1 WP_154508509.1 lipopolysaccharide assembly protein LapB

155(1) WP_154508527.1 WP_154508527.1 helix-turn-helix transcriptional regulator
155(1) WP_062252917.1 WP_062252917.1 helix-turn-helix domain-containing protein

156(1) WP_154508529.1 WP_154508529.1 OsmC family protein
156(1) WP_062252921.1 WP_062252921.1 OsmC family protein

157(1) WP_154508531.1 WP_154508531.1 manganese efflux pump MntP family protein
157(1) WP_062252923.1 WP_062252923.1 manganese efflux pump MntP family protein

158(1) WP_062252927.1 WP_062252927.1 hypothetical protein
158(1) WP_154508535.1 WP_154508535.1 protoporphyrinogen oxidase

159(1) WP_148214003.1 WP_148214003.1 flagellar motor switch protein FliG
159(1) WP_154510642.1 WP_154510642.1 flagellar motor switch protein FliG

160(1) WP_015416025.1 WP_015416025.1 acetate--CoA ligase family protein
160(1) WP_158947417.1 WP_158947417.1 acetate--CoA ligase family protein

161(1) WP_097013003.1 WP_097013003.1 ribonucleoside triphosphate reductase
161(1) WP_158947432.1 WP_158947432.1 ribonucleoside triphosphate reductase

162(1) WP_158947433.1 WP_158947433.1 anaerobic ribonucleoside-triphosphate reductase activating protein
162(1) WP_097013004.1 WP_097013004.1 anaerobic ribonucleoside-triphosphate reductase activating protein

163(1) WP_158947434.1 WP_158947434.1 DUF6765 family protein
163(1) WP_097013005.1 WP_097013005.1 DUF6765 family protein

164(1) WP_162140997.1 WP_162140997.1 CpaD family pilus assembly lipoprotein
164(1) WP_026986134.1 WP_026986134.1 CpaD family pilus assembly lipoprotein

165(1) WP_051384344.1 WP_051384344.1 RNA methyltransferase
165(1) WP_167122724.1 WP_167122724.1 RNA methyltransferase, partial

166(1) WP_169543107.1 WP_169543107.1 RND family transporter
166(1) WP_006001716.1 WP_006001716.1 efflux RND transporter permease subunit

167(1) WP_176290237.1 WP_176290237.1 TRAP transporter substrate-binding protein
167(1) WP_006000692.1 WP_006000692.1 TRAP transporter substrate-binding protein

168(1) WP_176290238.1 WP_176290238.1 flavocytochrome c
168(1) WP_006000659.1 WP_006000659.1 flavocytochrome c

169(1) WP_012624262.1 WP_012624262.1 MULTISPECIES: TSUP family transporter

169(1) WP_183717052.1 WP_183717052.1 sulfite exporter TauE/SafE family protein

170(1) WP_041724676.1 WP_041724676.1 MULTISPECIES: DMT family protein
170(1) WP_183717055.1 WP_183717055.1 DMT family protein

171(1) WP_012624276.1 WP_012624276.1 MULTISPECIES: helix-turn-helix domain-containing protein
171(1) WP_183717070.1 WP_183717070.1 helix-turn-helix domain-containing protein

172(1) WP_012624278.1 WP_012624278.1 MULTISPECIES: amino acid permease
172(1) WP_183717074.1 WP_183717074.1 amino acid permease

173(1) WP_012624279.1 WP_012624279.1 MULTISPECIES: DMT family transporter
173(1) WP_183717076.1 WP_183717076.1 DMT family transporter

174(1) WP_012624774.1 WP_012624774.1 MULTISPECIES: UDP-glucose dehydrogenase family protein
174(1) WP_183719690.1 WP_183719690.1 UDP-glucose dehydrogenase family protein

175(1) WP_183719716.1 WP_183719716.1 glutamate synthase-related protein
175(1) WP_072312477.1 WP_072312477.1 MULTISPECIES: glutamate synthase-related protein

176(1) WP_083577987.1 WP_083577987.1 MULTISPECIES: DNA-3-methyladenine glycosylase family protein
176(1) WP_183719722.1 WP_183719722.1 DNA-3-methyladenine glycosylase family protein

177(1) WP_183719724.1 WP_183719724.1 methylated-DNA--
177(1) WP_072312474.1 WP_072312474.1 MULTISPECIES: methylated-DNA--

178(1) WP_183720107.1 WP_183720107.1 NADP-specific glutamate dehydrogenase
178(1) WP_012624781.1 WP_012624781.1 MULTISPECIES: NADP-specific glutamate dehydrogenase

179(1) WP_193370332.1 WP_193370332.1 asparagine synthase (glutamine-hydrolyzing)
179(1) WP_193370325.1 WP_193370325.1 asparagine synthase (glutamine-hydrolyzing)

180(1) WP_167230595.1 WP_167230595.1 ion channel
180(1) WP_193371548.1 WP_193371548.1 potassium channel family protein

181(1) WP_193371549.1 WP_193371549.1 enoyl-CoA hydratase/isomerase family protein
181(1) WP_167230600.1 WP_167230600.1 enoyl-CoA hydratase/isomerase family protein

182(1) WP_193371550.1 WP_193371550.1 carboxyl transferase domain-containing protein
182(1) WP_167230602.1 WP_167230602.1 carboxyl transferase domain-containing protein

183(1) WP_085901365.1 WP_085901365.1 OmpA family protein
183(1) WP_193371566.1 WP_193371566.1 OmpA family protein

184(1) WP_011367398.1 WP_011367398.1 ABC transporter substrate-binding protein
184(1) WP_197706479.1 WP_197706479.1 ABC transporter substrate-binding protein

185(1) WP_012624779.1 WP_012624779.1 MULTISPECIES: diaminopimelate epimerase
185(1) WP_221277863.1 WP_221277863.1 diaminopimelate epimerase

186(1) WP_154508533.1 WP_154508533.1 spidroin-2
186(1) WP_223299901.1 WP_223299901.1 spidroin-2

187(1) WP_181017832.1 WP_181017832.1 ATP-dependent helicase HrpB
187(1) WP_223300000.1 WP_223300000.1 ATP-dependent helicase HrpB

188(1) WP_229595137.1 WP_229595137.1 hypothetical protein
188(1) WP_071544518.1 WP_071544518.1 hypothetical protein

189(1) WP_229595196.1 WP_229595196.1 hypothetical protein
189(1) WP_229595198.1 WP_229595198.1 hypothetical protein

190(1) WP_229772441.1 WP_229772441.1 hypothetical protein
190(1) WP_062252925.1 WP_062252925.1 hypothetical protein

191(1) WP_011792589.1 WP_011792589.1 DMT family transporter
191(1) WP_246388080.1 WP_246388080.1 DMT family transporter

192(1) WP_174406910.1 WP_174406910.1 hypothetical protein
192(1) WP_265826315.1 WP_265826315.1 hypothetical protein

193(1) WP_174406918.1 WP_174406918.1 VOC family protein
193(1) WP_265826324.1 WP_265826324.1 VOC family protein

194(1) WP_279137080.1 WP_279137080.1 alpha-glucan family phosphorylase
194(1) WP_011367393.1 WP_011367393.1 alpha-glucan family phosphorylase

195(1) WP_279137101.1 WP_279137101.1 DUF4851 domain-containing protein
195(1) WP_009301501.1 WP_009301501.1 MULTISPECIES: DUF4851 domain-containing protein

196(1) WP_281762959.1 WP_281762959.1 GNAT family N-acetyltransferase
196(1) WP_264983472.1 WP_264983472.1 GNAT family N-acetyltransferase

197(1) WP_285907348.1 WP_285907348.1 malic enzyme-like NAD(P)-binding protein
197(1) WP_155934516.1 WP_155934516.1 malic enzyme-like NAD(P)-binding protein

198(1) WP_083521996.1 WP_083521996.1 PIG-L deacetylase family protein
198(1) WP_288229165.1 WP_288229165.1 PIG-L deacetylase family protein

199(1) WP_407844440.1 WP_407844440.1 glutamate synthase
199(1) WP_183719714.1 WP_183719714.1 glutamate synthase

200(1) WP_027187802.1 WP_027187802.1 siroheme decarboxylase subunit beta
200(1) WP_420538654.1 WP_420538654.1 Lrp/AsnC family transcriptional regulator
