# Supplementary material for: Structural analyses uncover protease-adhesin interactions and c-di-GMP receptor regulation in sulfate-reducing bacteria
Source: Nat Commun. 2026 Apr 17;17:3564. doi: 10.1038/s41467-026-71936-5 (PMC13090378; doi:10.1038/s41467-026-71936-5)

Structural analyses uncover protease-adhesin interactions and c-di-GMP receptor regulation in sulfate-reducing bacteria

Maria E. Font, Amruta A. Karbelkar, Justin D. Lormand, Sofia Mortensen, Maria J. Garcia-Garcia, George A. O'Toole, Holger Sondermann

Supplementary Dataset 2

AlphaFold model summary

i. Modeled sequence
ii. Software
iii. Predicted aligned error (PAE) plots
iv. pLDDT score plots
v. AF models in two viewing angles (rotation by 180° around the y axis), colored according to pLDDT scores (gradient blue to red, from high to low confidence scores per residue)

Fig. 1

>DvhG

PGDTEAGGASPKAAPYEPRDGEAVTRGESEQRASSRHVIGQAPDPQQAPDARVIASEAPASPAHTDVRGERKVPGTT
GEDASSNRPEVSPVLEVAADAPLRHVRPEDMRGGAQQSPVQPSGQRGHRAGATSDGTPDGGEHSGNAATAEDGEGQG
VEAARPSRDAPARGQSSASSTAATGVRLFGTIEFRGQLKALPKWSRVVETERKKPGLYLDRALGGKGGQVWRELRGE
WQGLPLMERLKKVNTFFNQWPYRLDSENYGLPDYWATPDEFLRKSGDCEDYSIIKYFALKQLGVSADSMRIVVLLDK
IRGIAHAVLAVYDGNTAYILDNLSGLVLAHDFYKHYVPQYSVNESYRWAHIPLGKKAGRK

Software/Server: ColabFold version 1.5.5 (without template)

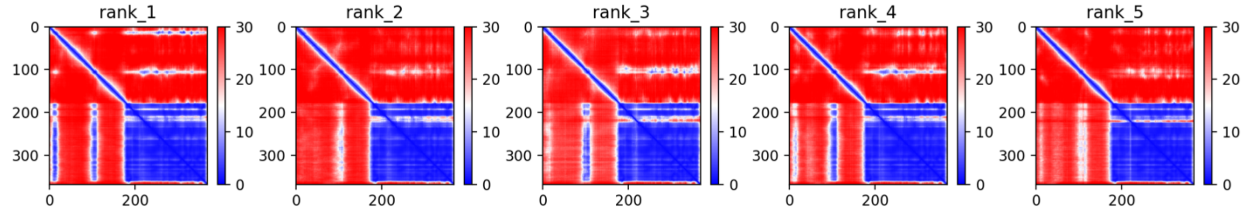

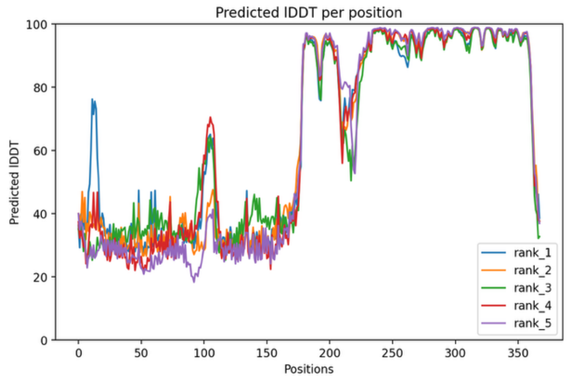

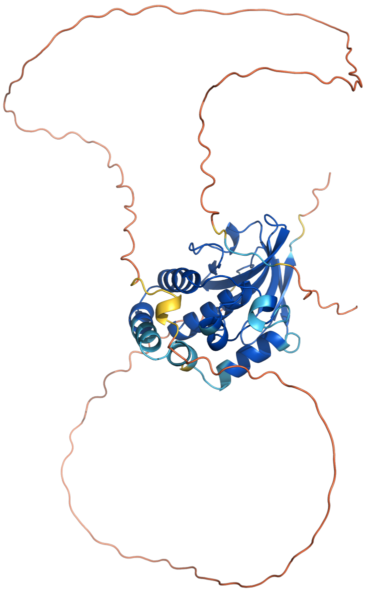

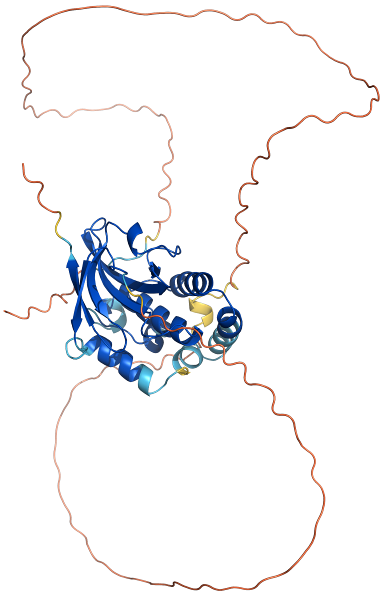

Fig. 2

>DvhA_part1

MPLNRTITPQQATAGTIRLPAPAVDEVITIQNAAGLKLALEFAPDAATTEKSGNDLVFTFPEGGQVIVSDFFAQLEG

GNVPTFVIEGQELPGDAFLTAFNAELLPAAGPGAGGGAGSGGVGDYTDDPGNLVDSVDRLGTLDPLVFERGTEPLPL

TDVGIVDNGVTITNIAPGGPDIGAGEGIVDETALASGSAPDAAGRTLTGDFT

Software/Server: ColabFold version 1.5.5 (without template)

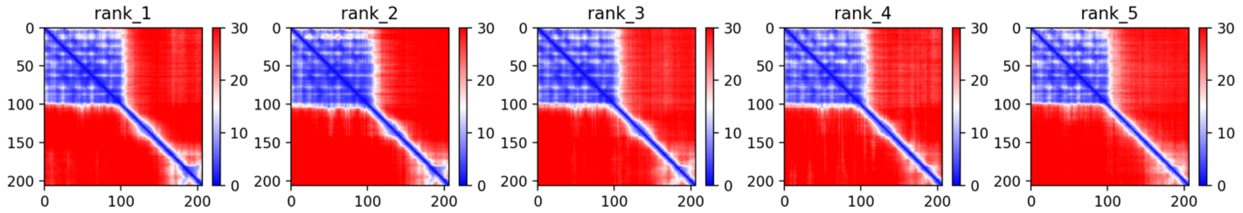

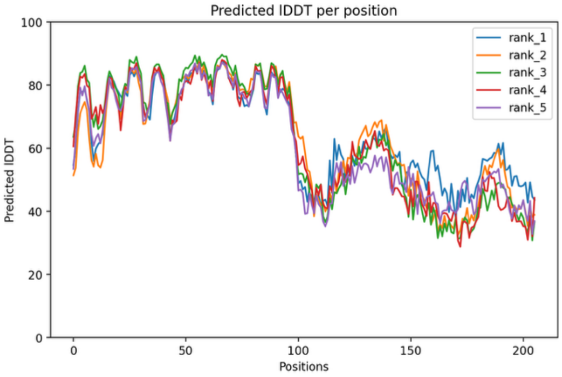

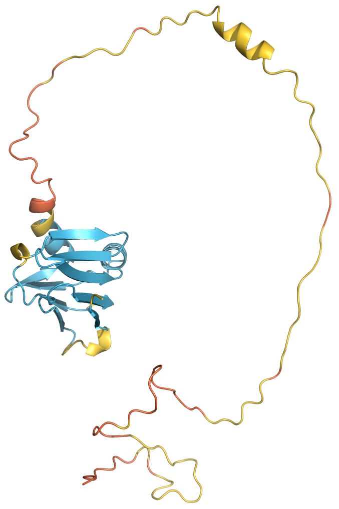

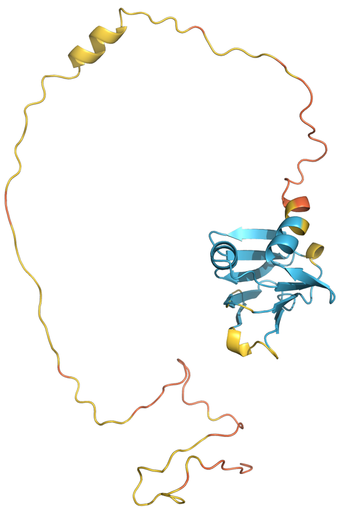

>DvhA_part2

ITAPDGLATVIIGGETVISGGALVNSTVTGDYGTLTITGYNPATGVITYSYELTGNADHTAGDDTIFETFPITATDT
DGDLASDSLNIQILDDAPVIIGGQAEVDETNFREGNTSGDVTPLLVSDTGDNPADPWGDGVIETQGTLNFSAGADGV
QSLVFDATIETNGPALTSQGAQVLFDVSDDGKTLRGYIVAEGDDAAPNGLTVFTMTIDNTGKYTYTQERPLDHSVDG
DALPGDPASTDHPHNDALQFNVGVVLTDTDTDTATGNITVVVRDDGPRVYGSEEFIERADYAAASDAPHASVADDAL
THSFNFTSGSTLADLISASTDANTGFVDIRMDGGTPYNGGANGLGGTYDGVQVGAADGPDRENELGYRDDKTSHEQE
ALVFKLNGVADHFEMDASGALSGSEGSGERGVVQFFLGDQLVGARTITSSTAGSIEFTGLFDRVVVTATDRTWLSTS
DNSDFYIKNVSFHEADAASVDIHSGMLYFDYGADRPGSFELVFDGIASGQQPEAESVEYRAAAPSDGADITDASIWT
LDGRRVSIEPERDSEGNPIAGTMVGHIINDDGTDSGQVAFTLTVNTATGEWTLAQRVPLDLPENGGKLGFSYTITDT
DGDTASGSFTVTVNEADRAPDIDMPPVAHVVDENGLVAGNDASLAAVEGAFRIDTNGEDLTSVSIAGVSFSVSNGVE
TINDLLGKVFNIGMNGKDVTVDGYLGVPSHATLEIIGVSGDATSGYSVQYRYTLKDNVRHGQDLDDMKTGDVIPVSV
TTTGADGHAQSASASLTVTIVDDTPTIDLAPAAGPVTEGQSIAGTWTHAMGADNDLGSSIKVVIGGNEYSINQNIYT
SKGILRVNGDGEWTFAAKNNLDHDYKQELKFALRITDSDGDTKLTDTITVAINDGEG

Software/Server: ColabFold version 1.5.5 (without template)

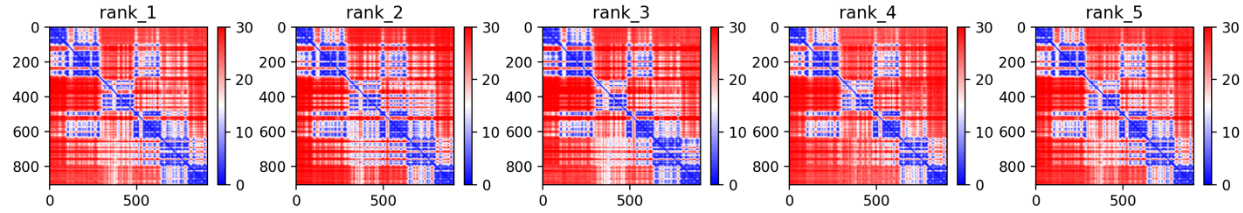

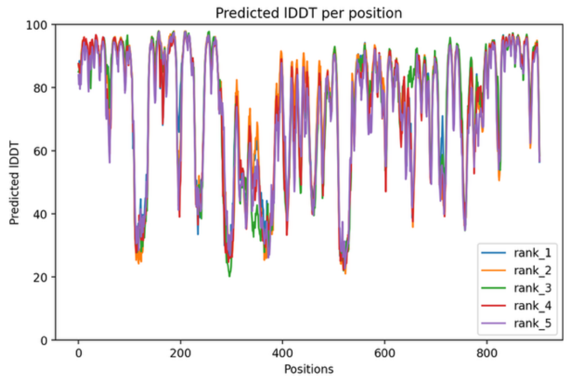

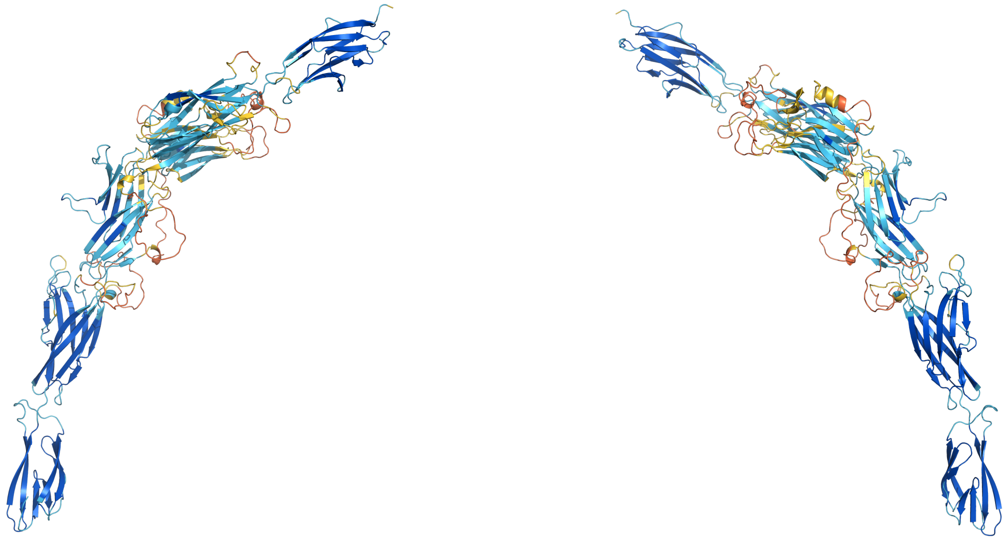

>DvhA_part3

PARAGTLTLTLDDDAASMLTRPDGYLDTDKTSGSLSFTAGSDDFESFKFVDASGVQMEGAGAALTWAVESGKLVGRY
SNGDKAVILSLSGGNIDAGETGNVTVHAELIDPLLHANGSDLVTITGIKVRGTDIDGDYVDGSVTLKVTDDMPVAQM
DADWVREDGKLIASGNVLSNDTAPGYDLSADGNRVTHIAKGDGAATDFSENGSTEIQGEYGTLTIYADGSYSYSLNN
DDPRVQHLNSKTDRLTETFKYTVTDGDGDASTAPLVITIVGTDDGVSIKGLEGSEGELYEKYLPGGTEDGLGSLVTA
EREFTIDVKDGLGSLTMSYGGQTVDIAEGAVLDTPYGNLTITGFDAVTGKVTYTFELDRNAPHTTPATDETFDLDIT
VTLTDRDNDSTSATINIDIKDDAPTLTVSNGAVTDDFGQSALVHVGFGADTHPGDGDMIAPGVQLQVNGGTPNFTSN
GLGVTYDGKPVAGSDGPDRENELGYRDDKTSGEQEGVVFELDGVANRFNMDAPGSFYTGGEGERGQVLFFLGNVQVA
SQAIPANGQFSYTGVLFDRVVVQAVDNSDGTTSSDNSDFYIKGVDVGFVDSIGKASGTVDMDYGADGEATSGALTFG
VGTTGIYTYDGHAVTVTPSADGKTLTGTYDGGKTAFTMSLDTTDGEWHFDQKVPLDLPGTTHKLGFNVGITDADGDT
TPAATIEVTVNEVERAPDIDTGLTPHVVHESGLNTADAAGSGLFPGGYDSASDPDGSPVVTEGTFRINANGESITSV
GIAGETISDLSTLVGRTFNIGANGQHVASGGHATLAITGYDASTGVVSYTYTLTDNVAHSTNVGGVLFDNEKTGDVI
PVSVTTGTGADASTATATITVTIVDDVPKAELNTTASADDSATLNVGQHTEGQFNMVPGADGVGQLLVNGVAFDATK
AGSDGYQSFAGDHGTLYVNADGEYRYDGTSVGNDSFTFTLVDGDGDRVADTLSLNVERGNTEPVLTMGDPVSVSEAA
LPDGSSPALSETTTGTFTFDDKGEGLQSLTVAGQTIDLGAFSTSGHTQTVVDDATGKLVITGYNSATGEVSYEYTLK
DNTLEGDNVQRTFEVKVTDTSGDEAKANIVIN

Software/Server: ColabFold version 1.5.5 (without template)

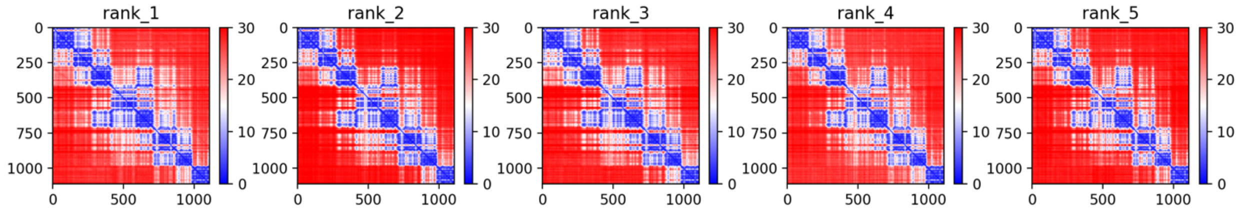

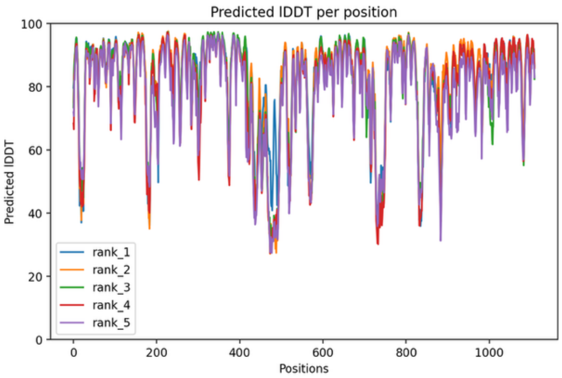

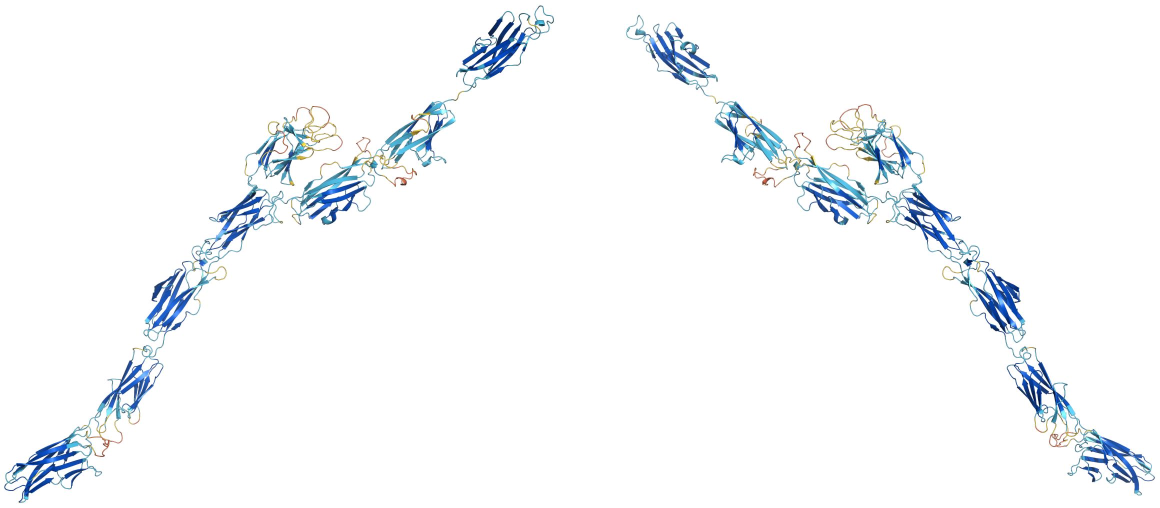

>DvhA_part4

VTDDAPTAHDDFATFPQVNTTTNLVIVLDTSGSMAWDSGVDGKSRMELAQEAIAKLMHAYDDMGHVNIKIVDFYSDA
DASKPWFEGNDAVAKATQYLTTDNKFVPGGGTDYDDATAATASALQTGMPNADRTVLYFFSDGAPDPISEALNATEE
KAWVDALKGVDNLDIAYAIGIGSQVSTTHLLPVGWERNPTDPDNPDAHSTKVLTDLSQLESTLLNTVPIEGNLLNND
VKGADGAYIEKVSFTSEDGTVYTATWNTSTGKLVFTTDLGGDGTIDNSVVQPGSRVTFSLGADRGTVEFDFSDGDFR
YVPGANPTGTPTFDYTIRDGDGDPSSAHLYLQLKGAAVDAKDNFASGIDEPRYIYDNDNDWWANDSHVHNVGAASEP
TFELSLGKNMVNDSVVASKEFSVNANDEVAFNWKAIIDGKVTNSPSSYDTDTFTIRIINLDTGKKEYEEQLFDGNPN
DIRSDSFSYNFKDTARYKVELIASDNDYDISSTKHSLDVQVSQFAIMAGYLAGNLIGDSPADALAGMTAHVTEVNGQ
LIAQDGSYTEITHEQYGSLKVNMYGDYEYRPAVGANGHDEAFVYKIATPTGQEDHATLNIHIGAAGDHTAQTTYSYA
YDDHNSATDTSMVYIGGSGDDTIVGTAGDDIILGGLGNDTLGGSGGRDYIDGGLGNDTIIIQDNSGDHRITDADFKG
LHGGAGTDTLEIHGDNVVLDFNNIADGKVSGIEHIDLGTDTGAQTVRLTAADLFDLAGTSEATAAGNKAVHISGDNA
DKVELVGSDWTHSTNAAGQDVYSVTVGSETRDLIIDHTIQQQIINSGG

Software/Server: ColabFold version 1.5.5 (without template)

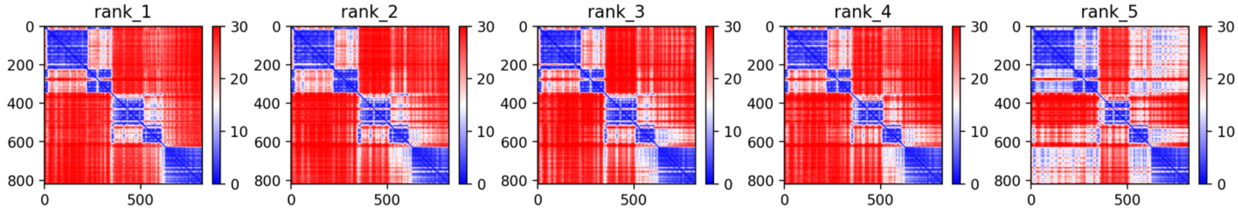

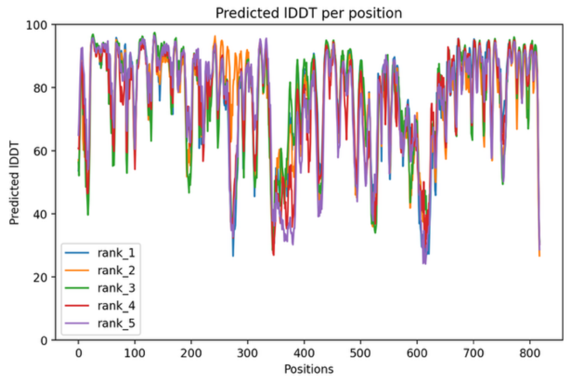

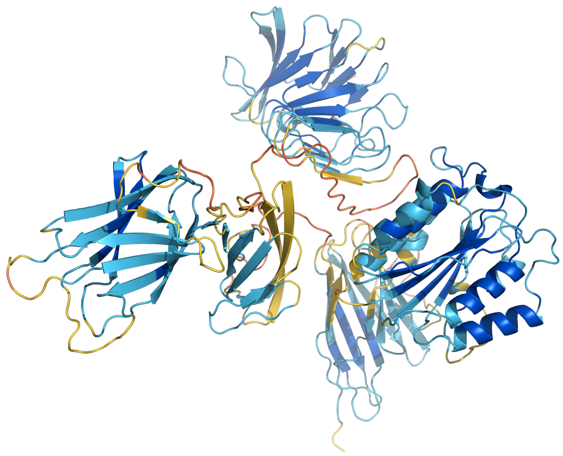

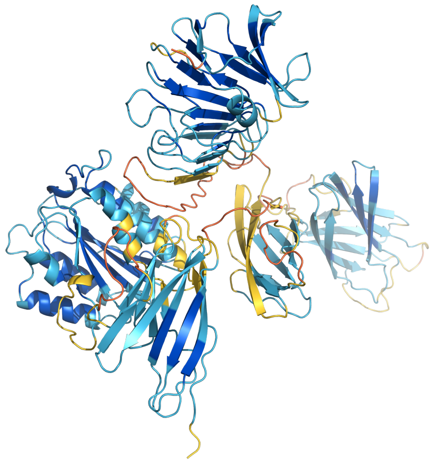

>DvhA_vWF

NTEPVLTMGDPVSVSEAALPDGSSPALSETTTGTFTFDDKGEGLQSLTVAGQTIDLGAFSTSGHTQTVVDDATGKLV
ITGYNSATGEVSYEYTLKDNTLEGDNVQRTFEVKVTDTSGDEAKANIVINVTDDAPTAHDDFATFPQVNTTTNLVIV
LDTSGSMAWDSGVDGKSRMELAQEAIAKLMHAYDDMGHVNIKIVDFYSDADASKPWFEGNDAVAKATQYLTTDNKFV
PGGGTDYDDATAATASALQTGMPNADRTVLYFFSDGAPDPISEALNATEEKAWVDALKGVDNLDIAYAIGIGSQVST
THLLPVGWERNPTDPDNPDAHSTKVLTDLSQLESTLLNTVPIEGNLLNNDVKGADGAYIEKVSFTSEDGTVYTATWN
TSTGKLVFTTDLGGDGTIDNSVVQPGSRVTFSLGADRGTVEFDFSDGDFRYVPGANPTGTPTFDYTIRDGDGDPSSA
HLYLQLKGAAVDAKDNFASGIDEPRYIYDNDNDWWANDSHVHNVGAASEPTFELSLGKNMVNDSVVASKEFSVNAND
EVAFNWKAIIDGKVTNSPSSYDTDTFTIRIINLDTGKKEYEEQLFDGNPNDIRSDSFSYNFKDTARYKVELIASDND
YDISSTKHSLDVQVSQFAIMAGYLAGNLIGDSPADALAGMTAHVTEVNGQLIAQDGSYTEITHEQYGSLKVNMYGDY
EYRPAVGANGHDEAFVYKIATPTGQEDHATLNIHIGAA

Software/Server: ColabFold version 1.5.5 (without template)

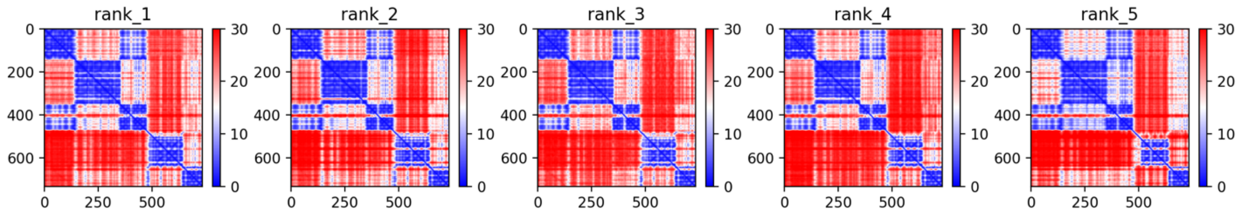

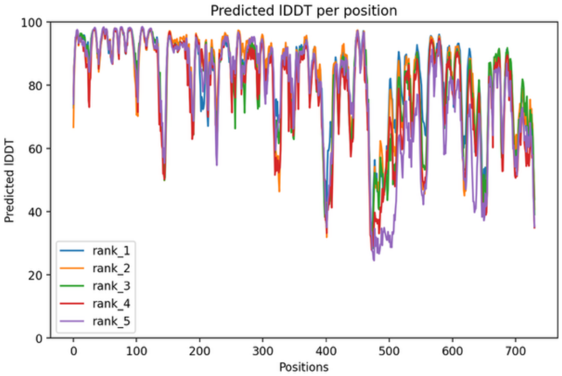

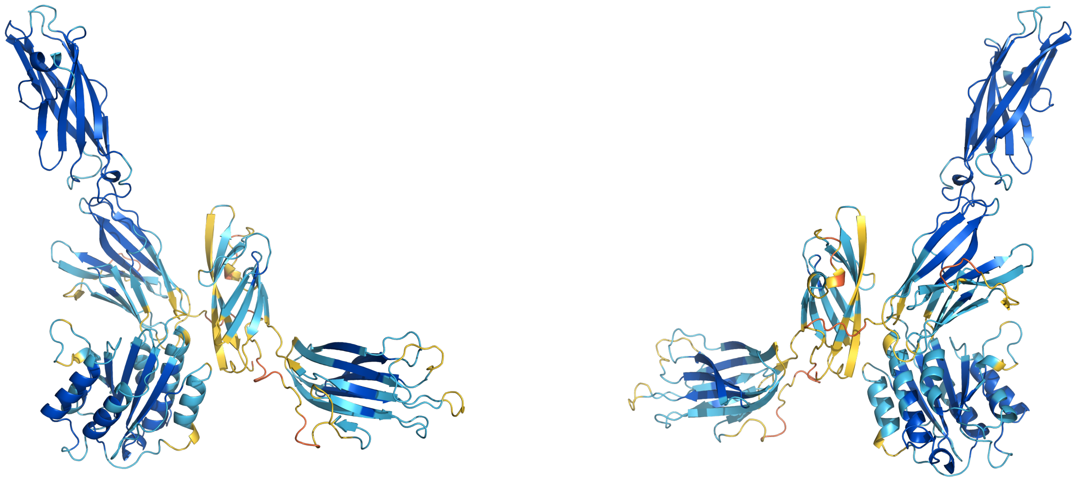

>DVU1545_part1

MPARTVEQHHSVAGAARILLDFPTDAATIEREGDALVFNFPDGARLVLDGFYTVTDGAELPDFILPDGTSFPGYDFL

AAIDAELLPAAGPGAGGGSGAGGGVGEYDDDAGRLVDSVDRLDPLTTTYWDRTTEPRIEDEGVIDPAGGALSITINT

TLPDGTISGTLGGFEDNQPGQHLGDATTTPIQLDVAFTPADNEELVSLTLSG

Software/Server: ColabFold version 1.5.5 (without template)

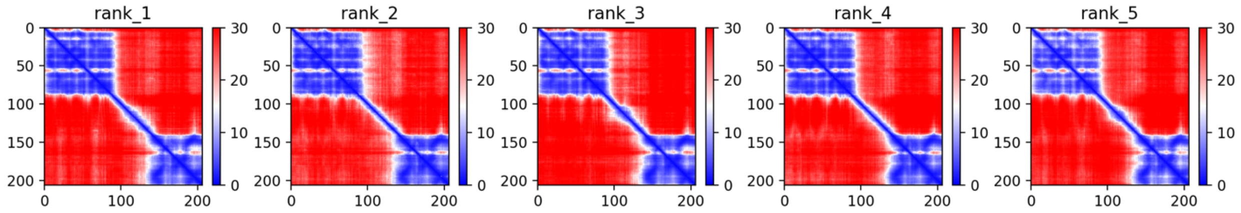

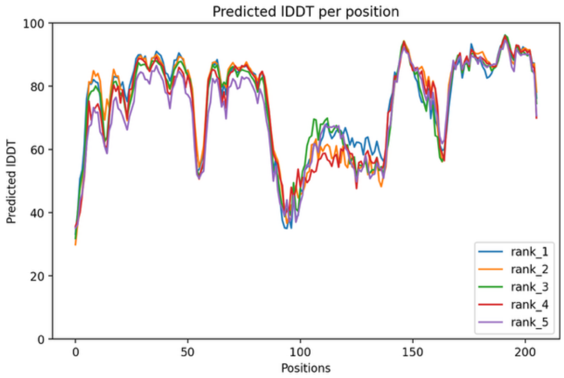

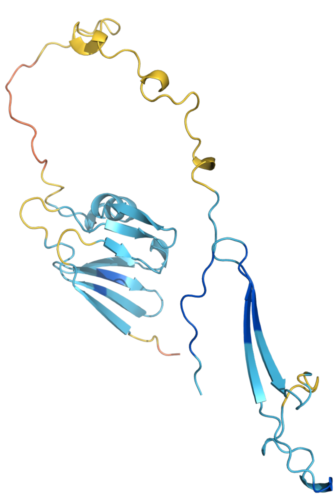

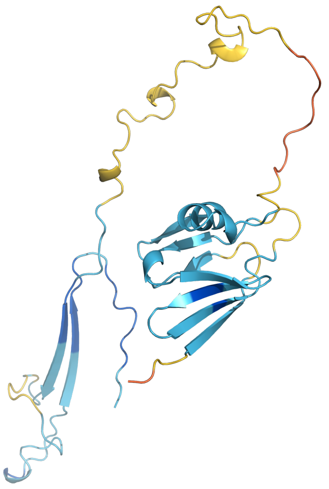

>DVU1545_part2

AGGALSITINTTLPDGTISGTLGGFEDNQPGQHLGDATTTPIQLDVAFTPADNEELVSLTLSGFDVGTSLSIGDPSF
GGALIVITAPDQSVTLTPGQLAEGIFIRPPEDSSRDMTLTANAVITDPDSGLTDTLSTTVQLVVDAVADRPVLEAET
PTPVATEETPVAIHVSGTFTDLDGSERHFIEVRDIPTDWVLGGTLPQGWIIVDPATHLPIATPAHALGENTTYTLCI
EVTGDTTAHAGGVNSGDSWAVEADLTFATRDWSDTRHGDGTPRPDGASGTTITVAAIAEESSATDGELTTGNNRAET
TTTITVSRTEDRPVIETSADVPGHAAHLEVDETPGLQTGDVAAAGLSATVLGIMTGFGLSADDALSATTGQVRFDLH
SDGVNDATPVASPLAGIAWDASQPLGGATLSTSRGGHPVTVGVVTSADGHSVLQGTYVDDAGTTRVAFIATLVADDL
AGSGSATVSFIQFEALAHPDGSSADEALNLPFRFTVTDDEGDTASSSVLLTLHDDGPTTAPDSVVFDEARTTGVDGN
VLANDSAGSDGYAEGGGVVGFSVTAGGITHSGLVPGDSIVVMNPGTGVPAGTFTLNADGSYVFTRAPGQDIDGTFTV
RVDYTVKDGDGDTATGRLDIALKPAPALHLSLTGDTQVYEDAAHGQRVNQAGNAGGSDGWNVSNDGSHDIATYLVQW
NNANGAPVAGMTTSGAFSFDVTITGVRQVGSSVGASGVFFDGDTTDNDTMAKTGDVTWAVPDGNGGWRPMHLDLTTD
AGKAALKSALNEALDDIYGGKLKVTDVSADGRLTFTVSDGCPLDAPLPIHVAAIDDRLGDSGERYSLQLGNLRPAEG
TPSSFDVAISGNRNVSTSIIDEGNSSESDGFRIGLESPTSAQESDGAARITLVLYDRDGNVYEGNEPPIQNIGVYLA
SSDGTAHVDADYFAATSRWVTPGEWIRVQGQDGHWRWEA

Software/Server: ColabFold version 1.5.5 (without template)

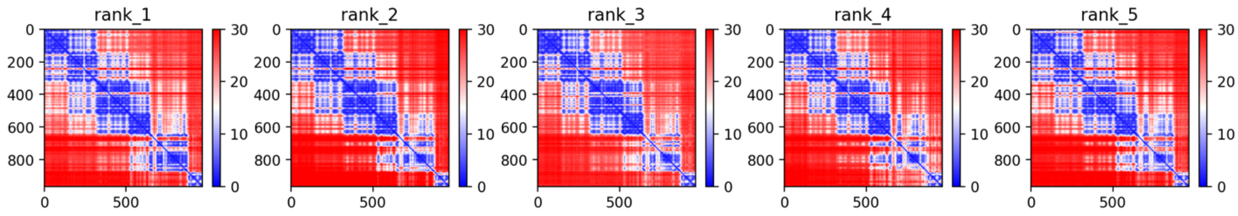

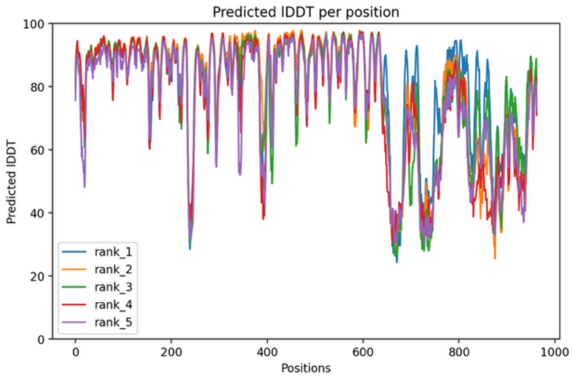

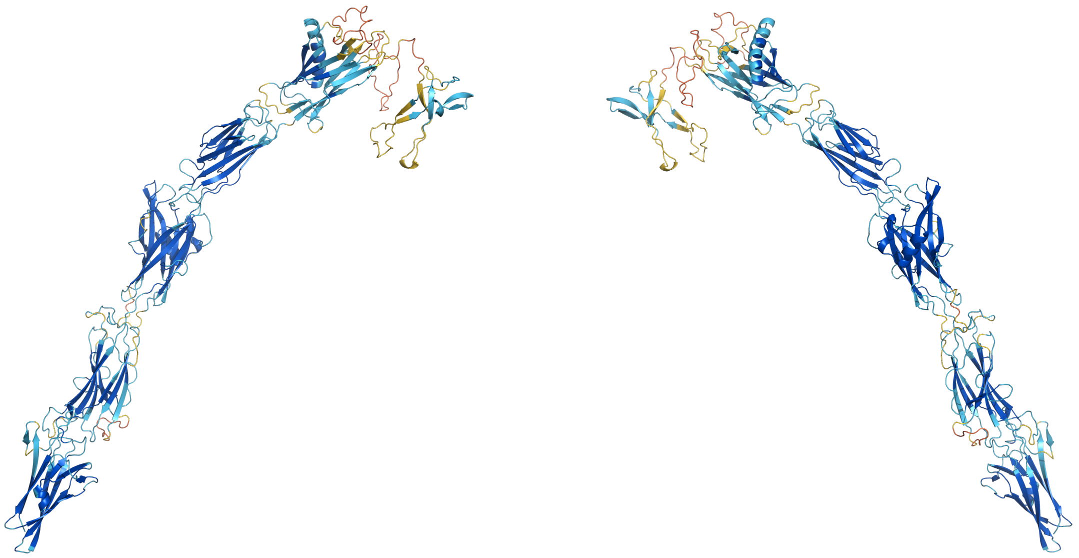

>DVU1545_part3

DGPAVYLDTKGDYATAPEGGEAAVHLGMTKTAVADFDVTLKVLDPSVLVPGSHAVLMIKTPGGEFVAHTTGVDIGQD
GRLTLTIPAGTSDAKVVFRTADDGIVGPHAPLEVGLVTVSGGEATQRGGVSVSSTFAEETYGSITYQTTGNAVPDGK
ATTTYTLNGVSFADVRSVRVDGAVVSISQNAAGKTIFTVTSNAGSSISDRITVTFVENANLTSNKANLSVSGVVTVS
GLVEIVDTTAHITAADVSLFALAGDGGDTSASGAIHHDALLAGAVDPSPHAQGIGFNADTTSASGIGHAGSGWVVGN
DGTLSFSGLEDGDRFTFKTVDLGNTADHDTAHLDVHVVQGVVFDGSSGEHGTGVVVSGSAQDDTITGTDGDDHLYGN
AGDDILRGGNGHDVLDGGTGNDTLHVHDTTGDGRITGDDFAGLHGGEGVDTILVEGDGTIIDFTAFAAGIVTGVENI
DLTAAGKQEVYLSANDVLHMGGELIISGTEADAVHLADAGTWTAEGTQTIDGMTYNVFAATVNAAGHEEQVHLMVQT
IVSTS

Software/Server: ColabFold version 1.5.5 (without template)

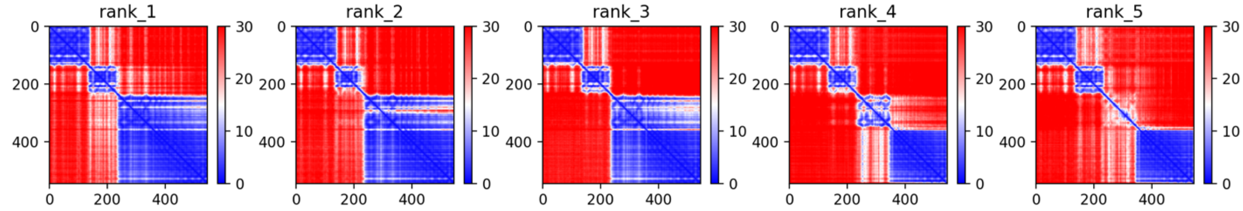

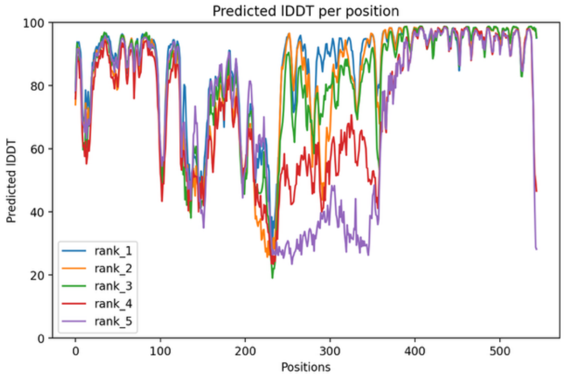

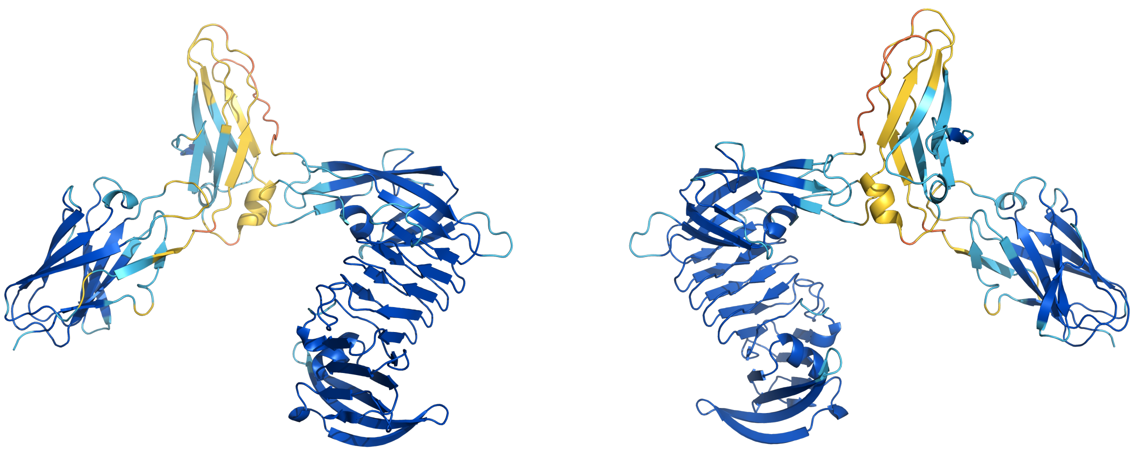

Fig. 3

>LapG_LapA

ADWDFSAISRKATALYGPLGAGQQRIDAWQNLLATQKQVSEMEKLKVVNLFFNKQMRYVEDIDLWHEVDYWETPIEA
LWKGAGDCEDYAIAKYFSLRHLGVASDKLRITYVKALRQNRAHMVLTYYSSPDAMPLVLDSLIDPIKPAAERTDLLP
VYSFNAEGLYLPGAKGNKKVGDTKRLSRWQDVLKKMQAEGFPVETTN:AQAAPSVEELQQAIAAGVDPTTALESTAA
GPSAAGTGGAAG

Software/Server: ColabFold version 1.5.5 (without template)

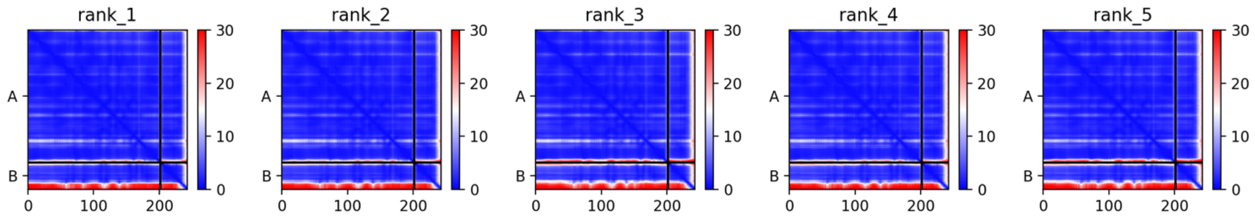

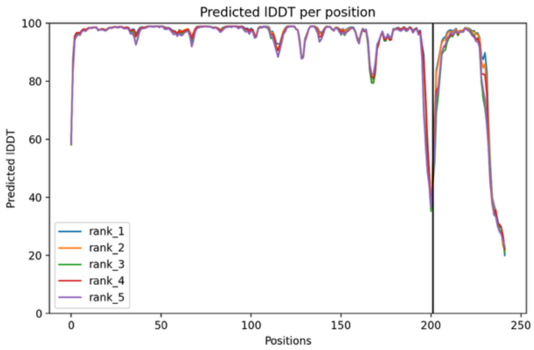

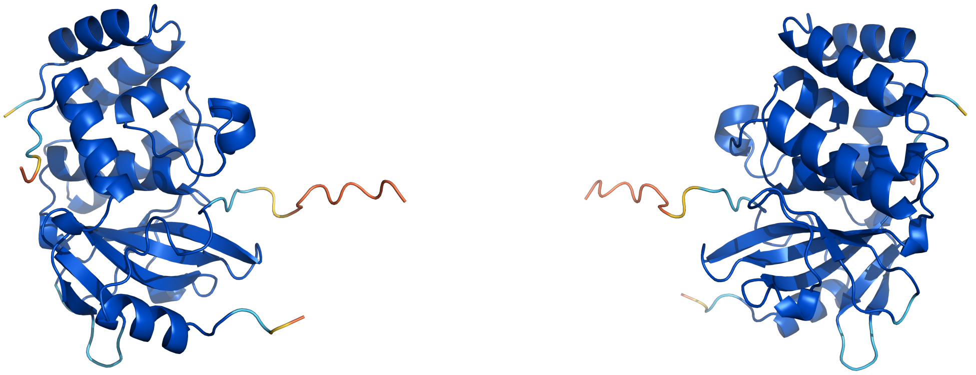

>DvhG_DvhA

ASSTAATGVRLFGTIEFRGQLKALPKWSRVVETERKKPGLYLDRALGGKGGQVWRELRGEWQGLPLMERLKKVNTFF
NQWPYRLDSENYGLPDYWATPDEFLRKSGDCEDYSIIKYFALKQLGVSADSMRIVVLLDKIRGIAHAVLAVYDGNTA
YILDNLSGLVLAHDFYKHYVPQYSVNESYRWAHIPL:MPLNRTITPQQATAGTIRLPAPAVDEVITIQNAAGLKLAL
EFAPDAATTEKSGNDLVFTFPEGGQVIVSDFFAQLEGGNVPTFVIEGQELPGDAFLTAFNAELLPAAGPG

Software/Server: ColabFold version 1.5.5 (without template)

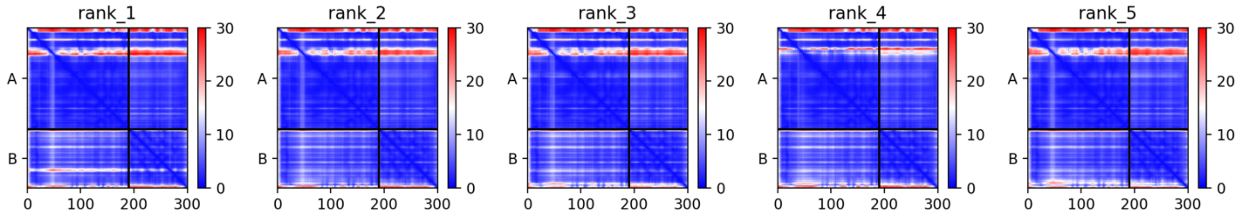

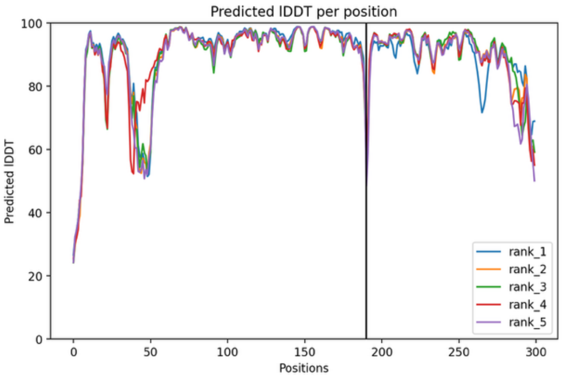

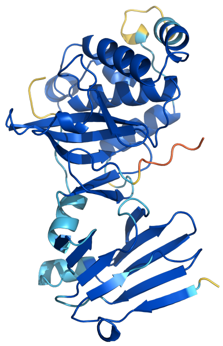

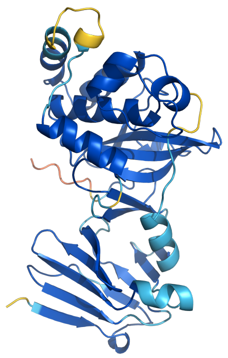

>DvhG_DVU1545

ASSTAATGVRLFGTIEFRGQLKALPKWSRVVETERKKPGLYLDRALGGKGGQVWRELRGEWQGLPLMERLKKVNTFF
NQWPYRLDSENYGLPDYWATPDEFLRKSGDCEDYSIIKYFALKQLGVSADSMRIVVLLDKIRGIAHAVLAVYDGNTA
YILDNLSGLVLAHDFYKHYVPQYSVNESYRWAHIPL:MPARTVEQHHSVAGAARILLDFPTDAATIEREGDALVFNF
PDGARLVLDGFYTVTDGAELPDFILPDGTSFPGYDFLAAIDAELLPAAGPG

Software/Server: ColabFold version 1.5.5 (without template)

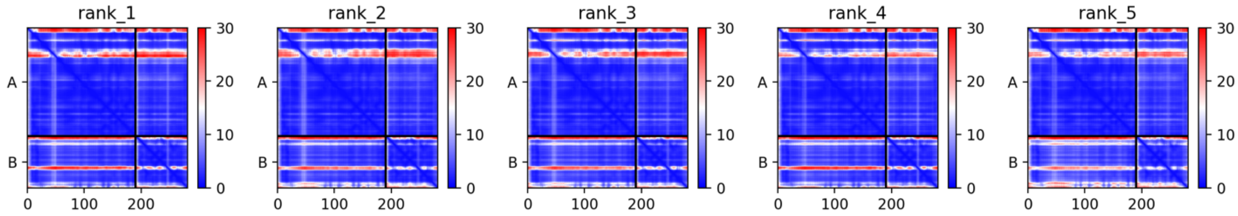

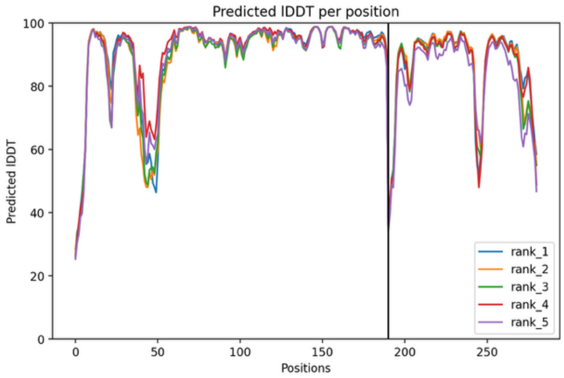

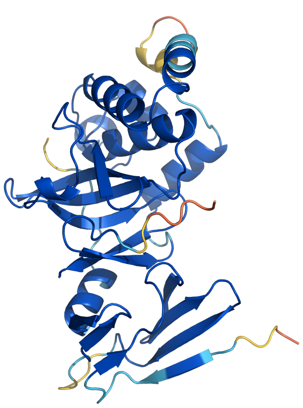

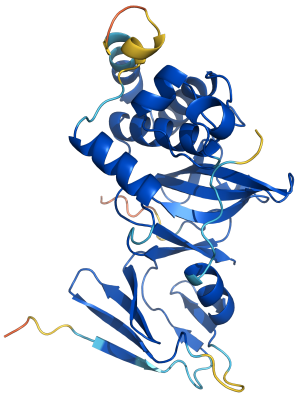

Fig. 5

>DvhD_dimer

MTQEWTTEEKTTPPMRDRRNIWRSRVLLFGLLVMVLTVTVAAIGAYSRIGAARAELEDDLARRQEVRLTGKVNAANL
WLDALKEQGDRLINADLFRLFAAEVDSLEGDVSLLFGASESGKAGSQTGQLASQLPLMRNLLREFVTYSDFIYGRIV
NARVQTYMTTDSTPTPMTADQQALAKRVLEEGKVVFAPLRNTTNGLVLDMFMPIFAPDFEQGEKAARPVAVLVLSKL
ASAKAGEMVGSAPLALEGARLHLVQKGAKGYQEVLPSTQEGLHPIDMPEGLADGDLAFGIRMGVGGKERVYSRAGVV
PAAGWWVFDEVPADVALASLKDHDRAVVVGAVLFSAVLLLVLAVLWWWLAGKEQREIADDFKRLFTVIDEQKHLLDS
INAAMRDPVSLTDASGVYHFVNHAFGEAVGRKPEDVIGLDVAAVFGFDTARRLTRSDATVIGEGKGQVEQETVFLQS
RRHVFQIVKTPMFTSESEHPRGVVSAFRDITELVDAQERSRKLVQQTIDAFITAIETKAPYLAGHSRGMSQFATAIA
RQMGLGERDVATVETAANLSQVGKIYVPSRLLTKPGALTAEEKAIVEEHVLHARRTLEHIEFDLPILDAIVQMNEHP
DGTGYPEHLKGDAIGIHARILAVANAFCAMVRPRSYRPALGVDAVIGVLRKEGGSFDAGVVDALARLLASPAGERLL
ESLDVRQG:MTQEWTTEEKTTPPMRDRRNIWRSRVLLFGLLVMVLTVTVAAIGAYSRIGAARAELEDDLARRQEVRL
TGKVNAANLWLDALKEQGDRLINADLFRLFAAEVDSLEGDVSLLFGASESGKAGSQTGQLASQLPLMRNLLREFVTY
SDFIYGRIVNARVQTYMTTDSTPTPMTADQQALAKRVLEEGKVVFAPLRNTTNGLVLDMFMPIFAPDFEQGEKAARP
VAVLVLSKLASAKAGEMVGSAPLALEGARLHLVQKGAKGYQEVLPSTQEGLHPIDMPEGLADGDLAFGIRMGVGGKE
RVYSRAGVVPAAGWWVFDEVPADVALASLKDHDRAVVVGAVLFSAVLLLVLAVLWWWLAGKEQREIADDFKRLFTVI
DEQKHLLDSINAAMRDPVSLTDASGVYHFVNHAFGEAVGRKPEDVIGLDVAAVFGFDTARRLTRSDATVIGEGKGQV
EQETVFLQSRRHVFQIVKTPMFTSESEHPRGVVSAFRDITELVDAQERSRKLVQQTIDAFITAIETKAPYLAGHSRG
MSQFATAIARQMGLGERDVATVETAANLSQVGKIYVPSRLLTKPGALTAEEKAIVEEHVLHARRTLEHIEFDLPILD
AIVQMNEHPDGTGYPEHLKGDAIGIHARILAVANAFCAMVRPRSYRPALGVDAVIGVLRKEGGSFDAGVVDALARLL
ASPAGERLLESLDVRQG

Software/Server: ColabFold version 1.5.5 (without template)

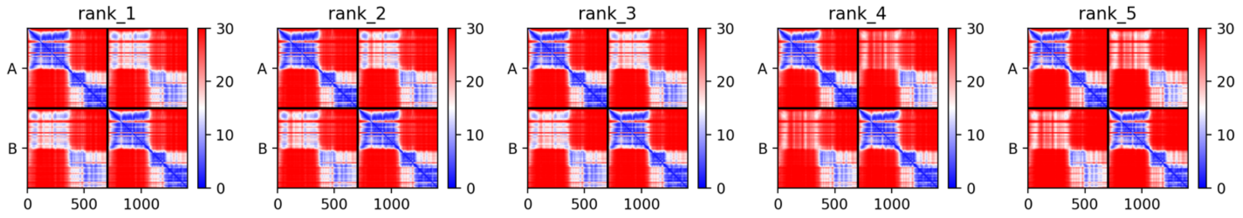

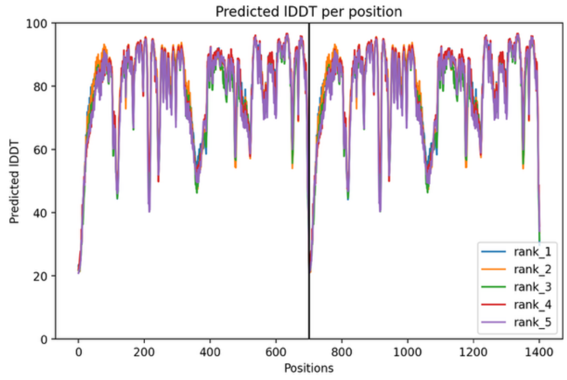

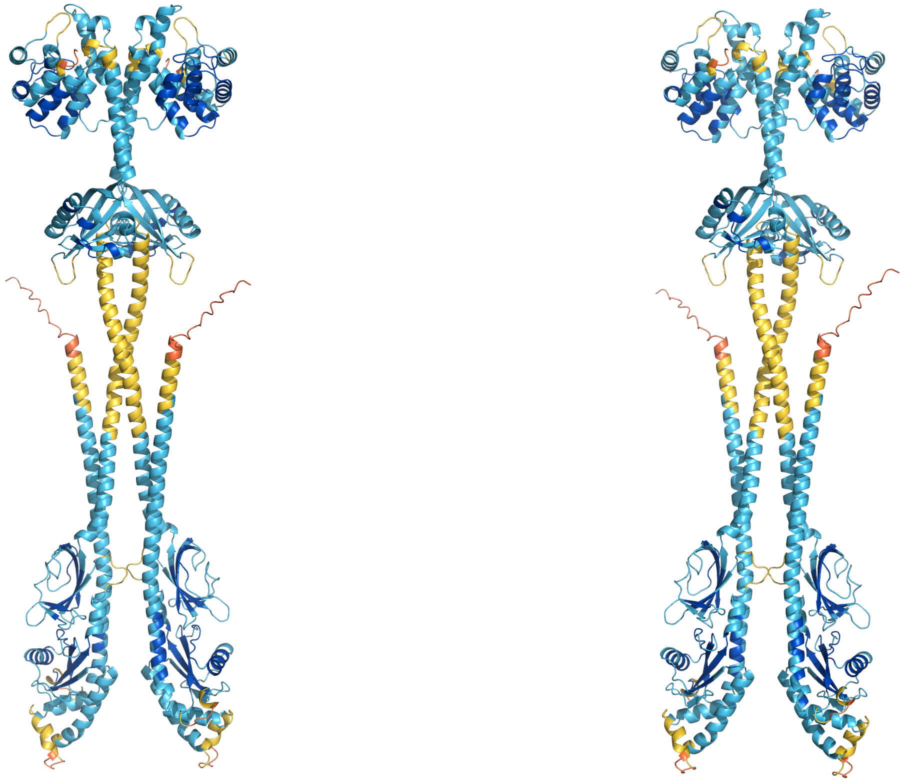

Fig. 10

>DvhD_DvhG

EDDLARRQEVRLTGKVNAANLWLDALKEQGDRLINADLFRLFAAEVDSLEGDVSLLFGASESGKAGSQTGQLASQLP
LMRNLLREFVTYSDFIYGRIVNARVQTYMTTDSTPTPMTADQQALAKRVLEEGKVVFAPLRNTTNGLVLDMFMPIFA
PDFEQGEKAARPVAVLVLSKLASAKAGEMVGSAPLALEGARLHLVQKGAKGYQEVLPSTQEGLHPIDMPEGLADGDL
AFGIRMGVGGKERVYSRAGVVPAAGWWVFDEVPADVALAS:STAATGVRLFGTIEFRGQLKALPKWSRVVETERKKP
GLYLDRALGGKGGQVWRELRGEWQGLPLMERLKKVNTFFNQWPYRLDSENYGLPDYWATPDEFLRKSGDCEDYSIIK
YFALKQLGVSADSMRIVVLLDKIRGIAHAVLAVYDGNTAYILDNLSGLVLAHDFYKHYVPQYSVNESYRWAHIPL

Software/Server: Alphafold3

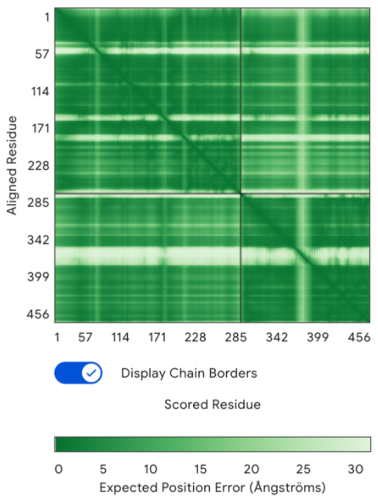

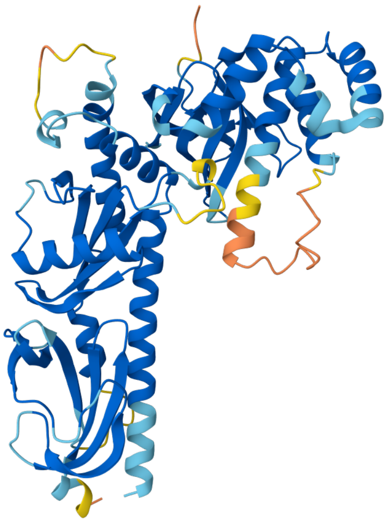

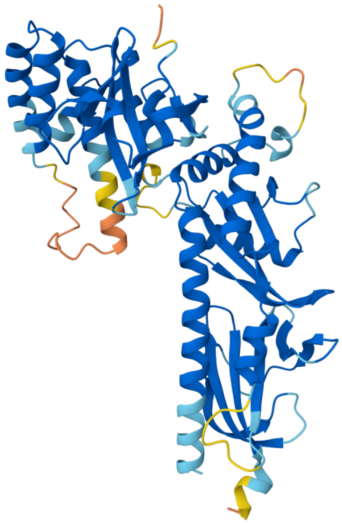

Supplementary Fig. 3

>LapG_MapA

ADWDFSAISRKATALYGPLGAGQQRIDAWQNLLATQKQVSEMEKLKVVNLFFNKQMRYVEDIDLWHEVDYWETPIEA

LWKGAGDCEDYAIAKYFSLRHLGVASDKLRITYVKALRQNRAHMVLTYYSSPDAMPLVLDSLIDPIKPAAERTDLLP

VYSFNAEGLYLPGAKGNKKVGDTKRLSRWQDVLKKMQAEGFPVETTN:APEAVTPSESQLTDVEQIQKAIAAGDDPT

KTAEATAAGPGATGGAPGAL

Software/Server: ColabFold version 1.5.5 (without template)

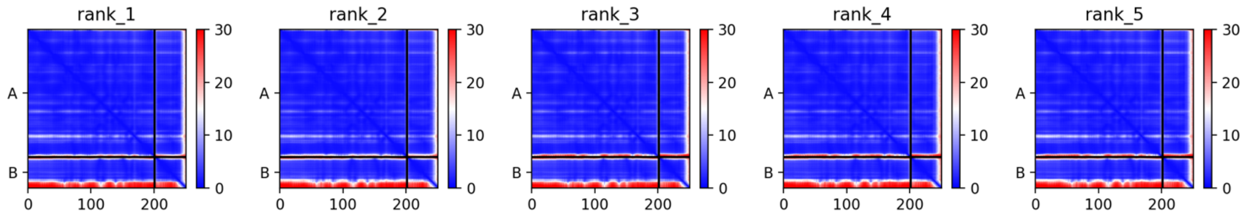

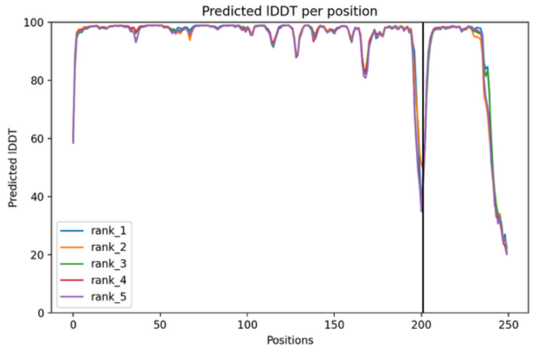

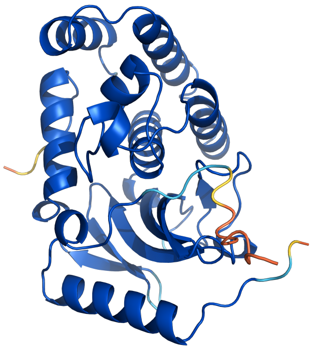

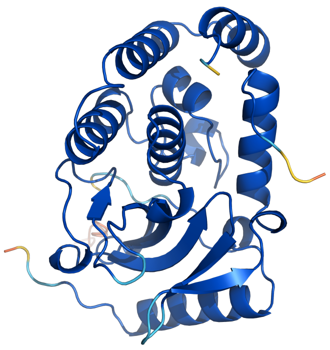

>LapG_CraA

LTSKEQQWVDAVKATYGQRAGKRVETWRRKMAELAPASEQEQLKEVNAFFNQLNFVNDIHLWGSKDYWATPLEFLGS
NAGDCEDFTIAKYFSLLELGVSDKKLRLVYVKAIELNQFHMVLAYYSTPSAEPVILDNINPQIKPASKRKDLLPIYS
FNGKNLWLMKSNNGQLAGDSSRLSLWNDLRARERSLKLNKPLVSYDE:ANFDEQAIAAIQQAILDGVDPTTALEAAA
AGAGAGGSANGGA

Software/Server: ColabFold version 1.5.5 (without template)

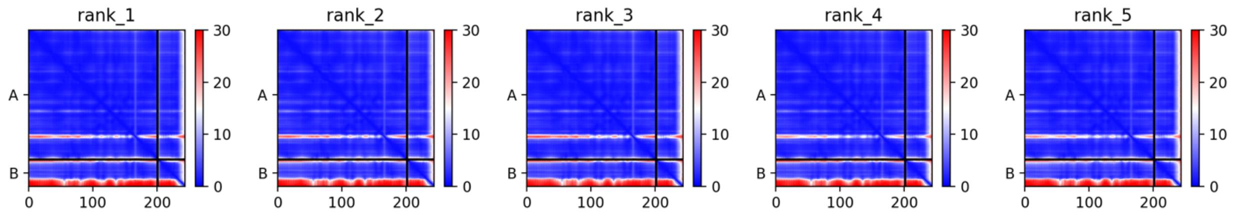

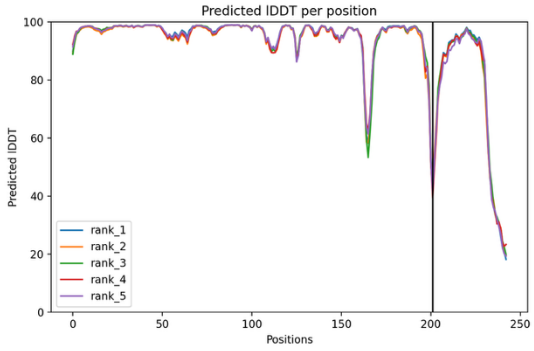

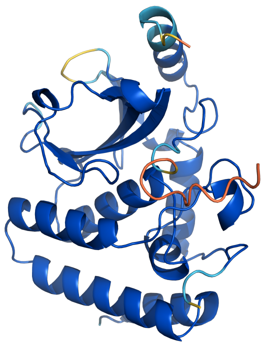

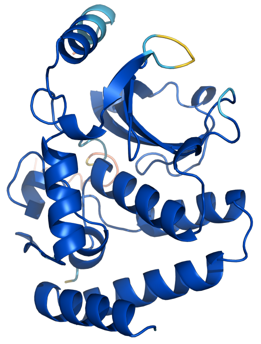

>LapG_FrhA

LTSKEQQWVDAVKATYGQRAGKRVETWRRKMAELAPASEQEQLKEVNAFFNQLNFVNDIHLWGSKDYWATPLEFLGS
NAGDCEDFTIAKYFSLLELGVSDKKLRLVYVKAIELNQFHMVLAYYSTPSAEPVILDNINPQIKPASKRKDLLPIYS
FNGKNLWLMKSNNGQLAGDSSRLSLWNDLRARERSLKLNKPLVSYDE:AEGNVQPITDDIEQILAALEEGADPTALD
DLAPAAGGLQGSSITGSA

Software/Server: ColabFold version 1.5.5 (without template)

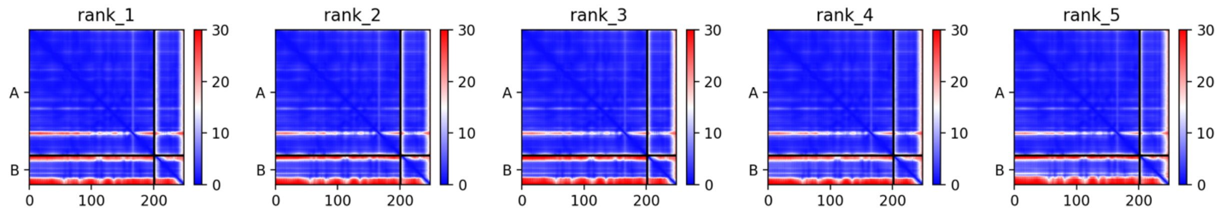

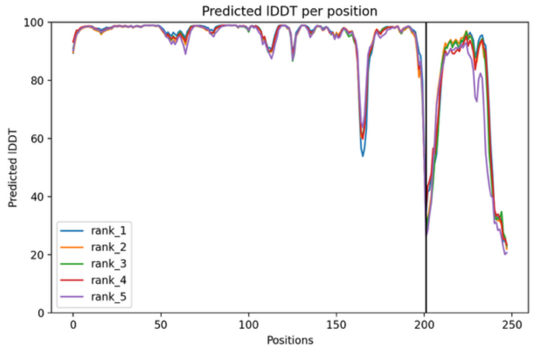

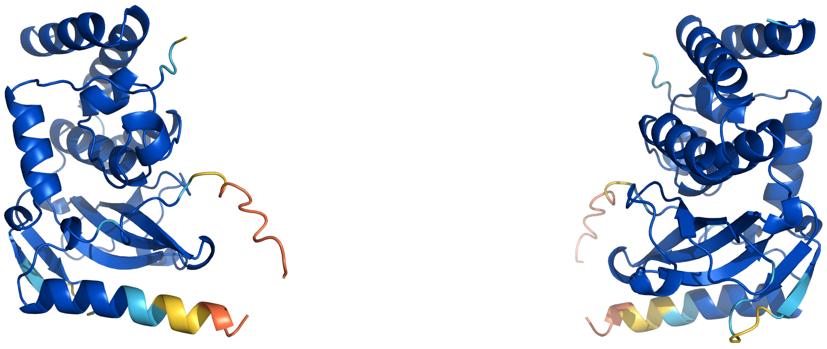

Supplement: Supplementary file 4 — Supplementary Data 2 [file 41467_2026_71936_MOESM4_ESM.pdf]
